# Structural Comparison Between MHC Classes I and II; in Evolution, a Class-II-Like Molecule Probably Came First

**DOI:** 10.3389/fimmu.2021.621153

**Published:** 2021-06-14

**Authors:** Yanan Wu, Nianzhi Zhang, Keiichiro Hashimoto, Chun Xia, Johannes M. Dijkstra

**Affiliations:** ^1^ Department of Microbiology and Immunology, College of Veterinary Medicine, China Agricultural University, Beijing, China; ^2^ Institute for Comprehensive Medical Science, Fujita Health University, Toyoake, Japan

**Keywords:** MHC, evolution, structure, class II, class I, b2-microglobulin, peptide

## Abstract

Structures of peptide-loaded major histocompatibility complex class I (pMHC-I) and class II (pMHC-II) complexes are similar. However, whereas pMHC-II complexes include similar-sized IIα and IIβ chains, pMHC-I complexes include a heavy chain (HC) and a single domain molecule β_2_-microglobulin (β_2_-m). Recently, we elucidated several pMHC-I and pMHC-II structures of primitive vertebrate species. In the present study, a comprehensive comparison of pMHC-I and pMHC-II structures helps to understand pMHC structural evolution and supports the earlier proposed—though debated—direction of MHC evolution from class II-type to class I. Extant pMHC-II structures share major functional characteristics with a deduced MHC-II-type homodimer ancestor. Evolutionary establishment of pMHC-I presumably involved important new functions such as (i) increased peptide selectivity by binding the peptides in a closed groove (ii), structural amplification of peptide ligand sequence differences by binding in a non-relaxed fashion, and (iii) increased peptide selectivity by syngeneic heterotrimer complex formation between peptide, HC, and β_2_-m. These new functions were associated with structures that since their establishment in early pMHC-I have been very well conserved, including a shifted and reorganized P1 pocket (aka A pocket), and insertion of a β_2_-m hydrophobic knob into the peptide binding domain β-sheet floor. A comparison between divergent species indicates better sequence conservation of peptide binding domains among MHC-I than among MHC-II, agreeing with more demanding interactions within pMHC-I complexes. In lungfishes, genes encoding fusions of all MHC-IIα and MHC-IIβ extracellular domains were identified, and although these lungfish genes presumably derived from classical MHC-II, they provide an alternative mechanistic hypothesis for how evolution from class II-type to class I may have occurred.

## Introduction

Major histocompatibility complex (MHC) molecules present peptide fragments of intracellularly digested protein antigens to T cells (1, 2). MHC class I (MHC-I) is expressed by most nucleated cell types and important for presentation of peptides from intracellular antigens to CD8^+^ cytotoxic T cells, which can kill the presenting cell in case of infection or cancerous mutations. MHC-II is expressed by professional antigen presenting cells and important for presentation of peptide fragments from digested endocytosed/phagocytosed antigens to CD4^+^ T cells, which help to decide on how to proceed with a possible immune response. Bjorkman et al. (3, 4) and Brown et al. (5) were the first to determine the structures of the ectodomains of pMHC-I and pMHC-II, respectively, namely of human pHLA-A2 and pHLA-DR1. The similarity between MHC-I and MHC-II, already evident from the sequences (6), was even more impressively observed in the structures (5). The ectodomain structures formed by both MHC classes consist of two membrane-distal domains which each constitute a similar half of a pseudo-symmetric unit consisting of a curved β-sheet topped by two antiparallel α-helix structures which leave a groove in between, and two membrane-proximal domains of the immunoglobulin superfamily (IgSF) C1 set. In MHC-I structures, a heavy chain (HC) comprising the two membrane-distal domains (I-α1 and I-α2), a membrane-proximal IgSF domain (I-α3), and a connecting peptide/transmembrane/cytoplasmic tail (CP/TM/CY) region, binds a free single IgSF domain molecule, β_2_-microglobulin (β_2_-m). On the other hand, MHC-II structures consist of two similarly sized molecules, the IIα and IIβ chains, which each possess a membrane-distal domain (II-α1 or II-β1), a membrane-proximal IgSF domain (II-α2 or II-β2), and a CP/TM/CY region. MHC-I molecules, within the groove—formed by their membrane-distal domains—which is closed at both ends, typically bind peptides of 8-11 amino acids (aa), most commonly of 9 aa, whereas MHC-II molecules typically bind peptides of 12-25 aa that extend beyond the ends of their open groove (7–9).

The above described structures and functions concern “classical” MHC-I and MHC-II and they are—except a few cases that represent gene losses [e.g (10, 11)]—found throughout jawed vertebrate species. The wide distribution and their conservation of ancestral traits suggest that these classical types are the evolutionary oldest types among known extant MHC-I and MHC-II types (12–15). However, at various times during evolution, classical MHC-I and MHC-II genes were duplicated and modified for encoding “nonclassical” MHC molecules which exhibit diverged features, a diversity of functions, and a more restricted distribution among species (12–18). In the present article, if not specified, “MHC-I” and “MHC-II” refer to the classical type molecules, with MHC-I referring to both HC and β_2_-m, and MHC-II referring to both MHC-IIα and MHC-IIβ.

**Figure 1:**
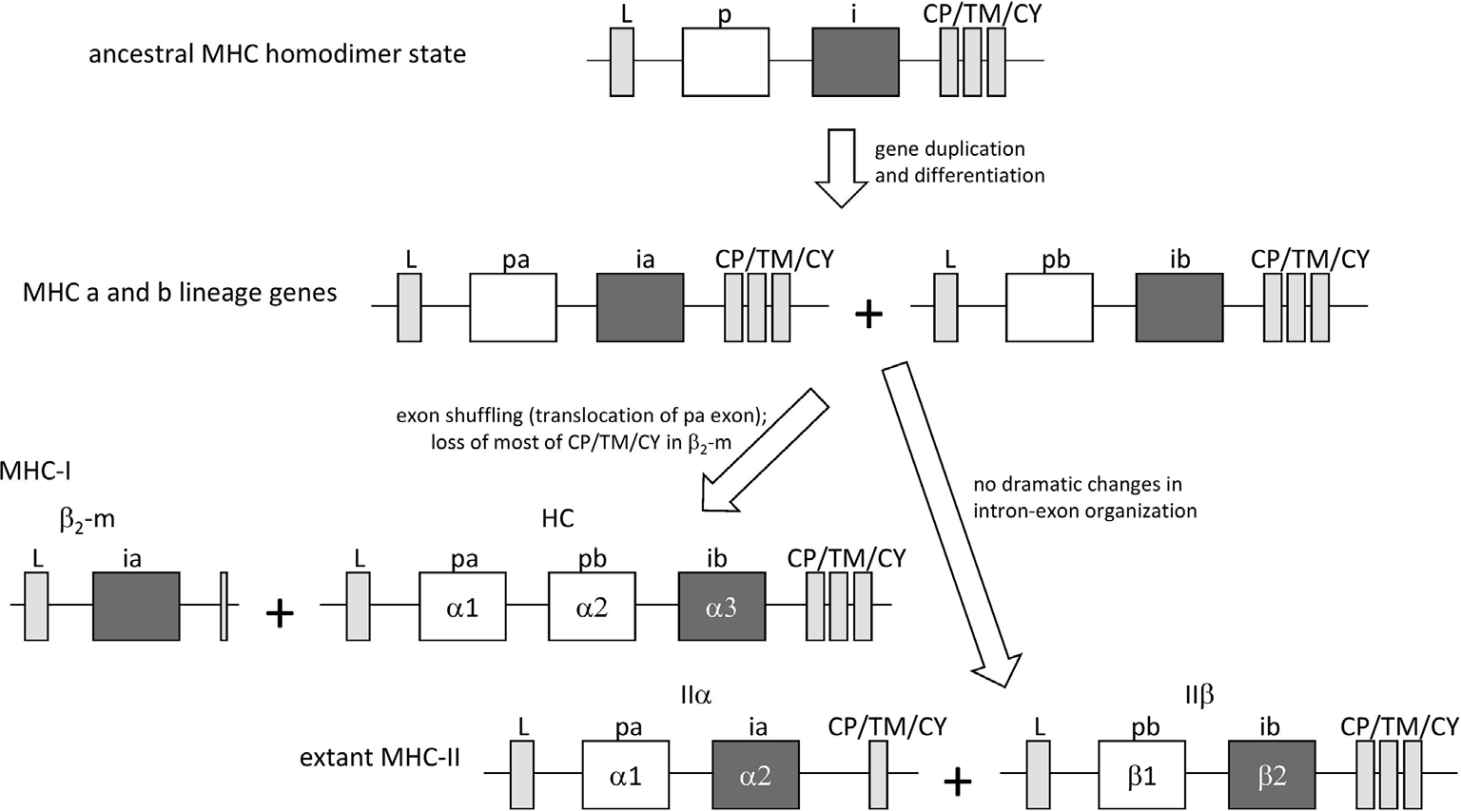
The “II-to-I” evolution model, at the gene level, for the creation of MHC-I and MHC-II from an MHC-II-like ancestor, similar to as proposed by Kaufman et al. (20, 21). In the model, MHC-I (HC + β2-m) and MHC-II (IIα + IIβ) gene sets were derived from an ancestral MHC-II-like gene that encoded a homodimer-forming molecule with a peptide binding domain (p) and an IgSF domain (i). The ancestral gene duplicated, followed by differentiation into lineages “a” (with domains pa and ia) and “b” (with domains pb and ib) that together encoded a heterodimeric structure. After this, an exon shuffling event involving translocation of the pa domain exon was part of the creation of the MHC-I genes HC and β2-m.

In jawless fish and invertebrates, MHC genes or MHC-homologous genes have not been found, and MHC features as well as the distinctions between MHC-I and MHC-II probably were established during the early evolution of jawed vertebrates (19). The first MHC molecule in evolution probably was a homodimer with class II-like features, as postulated by Kaufman et al. (20) based on sequence comparisons [see also (6)] and considerations of parsimony. The model was further explored and supported in later studies [e.g., (21–23)]. In the model, after gene duplication and differentiation, the homodimer evolved into a class II-type heterodimer molecule that was ancestral to both extant MHC-I and MHC-II [**Figure 1**, the model shown here is essentially as proposed by Kaufman et al. (20, 21)]. The generation of MHC-I genes HC and β_2_-m from MHC-II-type genes involved exon shuffling events (**Figure 1**). The model implies that the I-α1+ β_2_-m and II-α1+II-α2 domain sequences form a phylogenetic lineage, in the present study called the “a” lineage, while the I-α2+ I-α3 and II-β1+II-β2 sequences form the “b” lineage (**Figure 1**). The model is consistent with sequence-based computerized phylogenetic tree analyses (22, 24) and with structural analysis of pMHC-I and pMHC-II complexes (4, 5). The present article refers to this model of MHC class evolution as the “II-to-I” model. Some scientists do not subscribe to this model and proposed that MHC-I organization came before MHC-II organization (25, 26), but such I-to-II model cannot explain the close phylogenetic relatedness between the I-α1/II-α1 and I-α2/II-β1 sequences and between the β_2_-m/II-α2 and I-α3/II-β2 sequences.

Structures of pMHC-I have now been determined throughout the major clades of jawed vertebrates including cartilaginous fish (27), bony fish (28, 29), amphibians (30), the birds chicken [e.g., (31–33)] and duck (34), and a variety of mammals [e.g., (4, 35–38)], whereas pMHC-II structures only have been determined for chicken (39), mouse [e.g., (40)], and human [e.g., (5)]; most of the here listed studies in primitive vertebrates were performed by our group. This recent accumulation of MHC structural information across widely divergent species invites a deeper analysis of MHC evolution.

Here, we present a comprehensive analysis of MHC sequences and structures, and a deduction of major evolutionary developments within the course of pMHC structural evolution. Our study supports the II-to-I model and is the first to analyze pMHC structural evolution beyond the issue of covalent domain organization. It concludes that pMHC-II structures share important similarities with the deduced ancestral MHC homodimer, whereas pMHC-I structures acquired a significantly new peptide binding mode in which β_2_-m plays a pivotal role.

## Materials and Methods

### Alignment of MHC Sequences and Phylogenetic Tree Analysis

The sequence alignment between various MHC-I and MHC-II sequences was made with the intention to, as good as possible, align evolutionarily corresponding residues. MHC sequences and structures were retrieved from databases at the National Center for Biotechnology Information (NCBI; https://www.ncbi.nlm.nih.gov/). Representative MHC sequences were aligned by hand [see also (12–14, 41)] based on similarities between sequences, considerations of likely evolutionary events, and structural comparisons of various pMHC-I and pMHC-II (**Supplementary File 1B**). For most parts of the β-strands and helices the alignments are unambiguous, but in many of the loop regions, and in some β-strand and helical regions with insertions or deletions, the best possible alignment is uncertain. That superimposing of structures does not provide definite clues for all parts of the alignment can be seen in **Supplementary File 1B**. Secondary structures as indicated in the sequence alignment figures were determined by DSSP software [https://swift.cmbi.umcn.nl/gv/dssp/ (42)]. Leader sequences were predicted using SignalP software [http://www.cbs.dtu.dk/services/SignalP/ (43)]. For alignment of lungfish MHC-IIabSol sequences only, we used CLUSTAL 2.1 software (https://www.genome.jp/tools-bin/clustalw). For phylogenetic tree analysis of lungfish MHC-IIabSol and classical MHC-II sequences the neighbor joining method was applied using MEGA7 software (44).

### Calculations and Generation of Illustrations

Peptide-contacting residues were identified using the program CONTACT and were defined as residues containing an atom within 4.0 Å of the target partner (45). Structural illustrations and the electron density-related figures were generated using the PyMOL molecular graphics system (http://www.pymol.org/), and the same software system was used for making structural superposition figures by using the program “super” and to calculate dihedral angles of the pep56 backbone using the program “Measurement - Dihedrals”. PDBePISA software [http://www.ebi.ac.uk/msd-srv/prot_int/cgi-bin/piserver (46)] was used for interdomain contact analysis and for measuring the exposed surface areas (=Accessible Surface Area minus Buried Surface Area) of the peptide ligands. Unless mentioned otherwise, the PDB accessions of the pMHC structures shown as representative structures are: Shark UAA, nurse shark (*Ginglymostoma cirratum*) Gici-UAA*01, 6LUP; Carp UAA, grass carp (*Ctenopharyngodon idella*) UAA, 5Y91; Frog UAA, African clawed frog (*Xenopus laevis*) UAAg, 6A2B; Chicken (*Gallus gallus*) BF2*0401, 4E0R; HLA-A2, 3PWN; Chicken (*Gallus gallus*) BL2*01901, 6KVM; Mouse (*Mus musculus*) H2-Ag7, 1F3J; HLA-DR1, 1AQD. For comparisons of a large number of pMHC-Is, structures with the following PDB accession numbers were analyzed: HLA-A*0101 (4NQV), HLA-A*0201 (3PWN), HLA-A*0203 (3OX8), HLA-A*0206 (3OXR), HLA-A* A0207 (3OXS), HLA-A*0301 (3RL1), HLA-A*1101 (1X7Q), HLA-A*2402 (2BCK), HLA-A*6801 (4HX1), HLA-A*6802 (4HWZ), HLA-B*0701 (3VCL), HLA-B*0801 (1M05), HLA-B*1402 (3BVN), HLA-B*1501 (1XR8), HLA-B*1801 (4JQV), HLA-B*2705 (1HSA), HLA-B*2709 (1UXW), HLA-B*3501 (1A9E), HLA-B*3505 (4JRX), HLA-B*3508 (3VFR), HLA-B*3901 (4O2C), HLA-B*4103 (3LN4), HLA-B*4402 (1M6O), HLA-B*4403 (1N2R), HLA-B*4405 (1SYV), HLA-B*4601 (4LCY), HLA-B*5101 (1E27), HLA-B*5201 (3W39), HLA-B*5301 (1A1O), HLA-B*5701 (2RFX), HLA-B*5703 (2BVO), HLA-C*0801 (4NT6), HLA-C*CW3 (1EFX), HLA-C* CW4 (1IM9), H-2*Db (1WBX), H-2*Dd (3E6H), H-2* Kb (3TID), H-2*Kd (1VGK), H-2*Kk (1ZT1), H-2*Kw_M7 (3FOL), H-2*Ld (1LD9), RT1-Aa (1ED3), RT1-Ac (1KJV), Mamu-A1 (1ZVS), Mamu-B17 (3RWC), SLA-1*0401 (3QQ3), BoLA-N*1801 (3PWU), BF2*0401 (4E0R), BF2*2101 (3BEV), Xela-UAA (6A2B), Ctid-UAA (5Y91), Gici-UAA (6LUP). For comparisons of a large number of pMHC-IIs, those were: HLA-DR1 (1AQD), HLA-DR2 (1YMM), HLA-DR3 (1A6A), HLA-DR4 (1D5M), HLA-DR5 (2Q6W), HLA-DR14 (6ATZ), HLA-DR15 (5V4M), HLA-DQ2.3 (4D8P), HLA-DQ8 (1JK8), HLA-DQ0602 (1UVQ), HLA-DM (1HDM), HLA-DM-DE (4FQX), HLA-DM-DO (4I0P), HLA-DP2 (3LQZ), HLA-DP5 (3WEX), HLA-DP-TCR (4P4K), HLA-DQ1-TCR (3PL6), HLA-DQ2-TCR (4OZH), HLA-DQ2.5-TCR (4OZF), I-Ab (1LNU), I-Ad (1IAO), I-Ak (1IAK), I-Ag (1ES0), I-Au (1K2d), I-EK (1FNG), Chicken-B-LA (6KVM).

The PDB accession numbers and residue positions of the non-MHC C1 set IgSF domains compared in **Figure 10B** were as follows: Ig-L, 1A4J A/113-217; Ig-H-C1, 1MCP L/119-220; IgNAR-C1, 4Q97 A/139-241; IgNAR-C2, 4Q9B A/243-343; TCR-beta, 1BD2 E/124-247; HFE, 1A6Z C/181-275; MIC, 1B3J A/181-274; M144-A3, 1PQZ A/142-242; ZAG-A3, 1T7V A/186-277; FcRn, 1EXU A/180-267; SIRPa-C1, 2WNG A/116-222.

### Amplification and Sequencing of *MHC-IIabSol* for Slender Lungfish (*Protopterus dolloi*)

A slender lungfish (*Protopterus dolloi*) was obtained from Meitosuien Co. Ltd. (Nagakute, Aichi, Japan). The animal was handled according to the Guidelines for the Management of Laboratory Animals in Fujita Health University. Total RNA was isolated from kidney by use of “TRIzol” (Gibco) following the instructions of the manufacturer, except that the protocol was repeated for additional removal of DNA and proteins. SuperScript III reverse transcriptase kit (Invitrogen) with random hexamer primers was used for the construction of cDNA. PCR was conducted using ExTaq DNA polymerase (Takara) with 40 PCR cycles and primers *MHC-IIabSol-F*, 5’-AGAACCGTTTGGCACTGGGATC, and *MHC-IIabSol-R*, 5’-TTCTGAAGCACATCAGTAATACTGCCTG. The amplified fragment was inserted into plasmid vector, followed by transformation to *E.coli*, and sequence analysis of multiple clones (to minimize the chance of PCR or sequencing artefacts) by using BigDye Terminator v3.1 Sequencing Standard kit (Applied Biosystems) and 3100Avant/3130xl Genetic Analyzer (Applied Biosystems Life Technologies, Foster City, CA, USA). The purified *MHC-IIabSol* sequence was confirmed by its identity with SRA reports, and was deposited to GenBank as accession MT909553.

## Results

### Overall pMHC-I and pMHC-II Structures, and Introduction of a Nomenclature for Similar Domains

**Figure 2:**
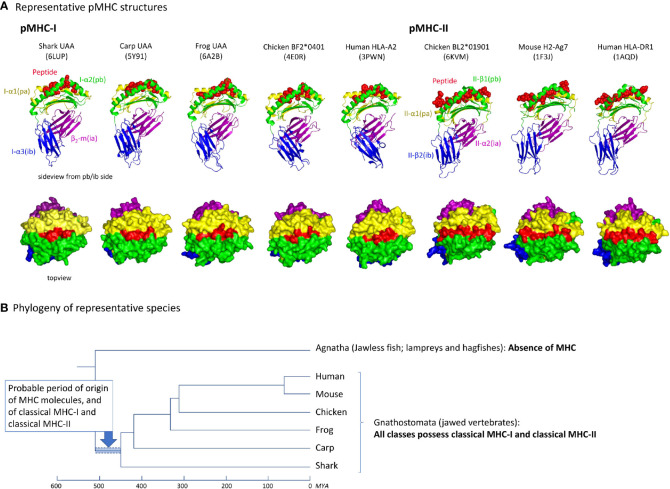
**(A)** Representative pMHC-I and pMHC-II structures of different species. PDB accession numbers are between brackets. The top row shows side views, with the MHC molecules in cartoon format and the peptide in spheres format. The bottom row shows top views, in surface format. **(B)** Cladogram showing the phylogeny of the species shown in **(A)** (47, 48). Because in Agnatha no MHC genes are found and all classes of Gnathostomata possess both classical MHC-I and MHC-II, it can be assumed that MHC genes emerged and differentiated into classical MHC-I and MHC-II in the period highlighted by the blue bar. MYA, million years ago.

A comparison of five and three representative pMHC-I and MHC-II structures, respectively, of different species, reveals an overall similarity in organization (**Figure 2A**). Their most readily observed distinction is the confinement of the peptide within a closed groove in the case of pMHC-I (4, 5). For the phylogeny of the species whose pMHC structures are shown in **Figure 2A**, see **Figure 2B**. As reported previously [e.g., (19)], the absence of MHC genes in jawless vertebrates and invertebrates, combined with the presence of classical MHC-I and classical MHC-II in all extant classes of jawed vertebrates, concludes a relatively rapid emergence and differentiation of MHC molecules early in jawed vertebrate evolution (**Figure 2B**).

**Figure 3:**
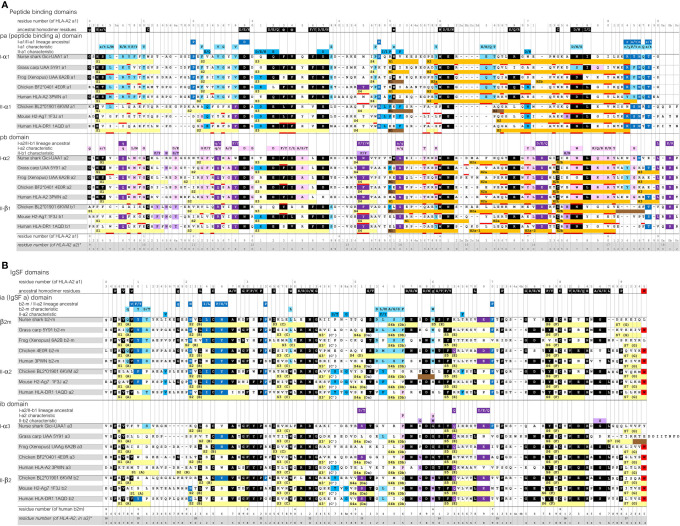
Sequence alignment of representative MHC-I and MHC-II molecules for which the structure is known. **(A)**, pa and pb domains; **(B)**, ia and ib domains. Yellow, light orange, orange, and brown bars represent β-strands, π-helices, α-helices, and 3_10_-helices, respectively. Residues that are within 4.0 Å distance of the peptide ligand are red underlined. The β-strands are numbered S1, S2, and so on, but for the IgSF domains also the alternative A-to-G names are shown. Uninterrupted stretches of helical structures (α-helices and/or 3_10_-helices) in the pab domain that are separated by kinks were named H1 and H2 for the pa domain, and H1, H2a (or H2a-1 and H2a-2), H2b and H3, for the pb domain (49, 50). In the shark UAA I-α2 domain the “kink” between the H2a and H2b helices is in the shape of a π-helix. Residue coloring refers to conservation of (sets of) residues indicated above the alignments: black, inherited from the MHC homodimer ancestor; dark blue, ancestral to the I-α1+β_2_-m/IIα lineage; purple, ancestral to the I-α2+I-α3/IIβ lineage; light blue, characteristic for the I-α1+β_2_-m lineage; pink, characteristic for the I-α2+I-α3 lineage. Small font of color-shaded letters above the alignment indicates uncertainty about the evolution pattern. PDB accessions for structures of the depicted sequences are: Nurse shark UAA*01, 6LUP (note: recombinant 6LUP β_2_-m possesses residue iaS3 and not the natural iaG3); Grass carp UAA, 5Y91; Frog (Xenopus) UAA, 6A2B; Chicken BF2*0401, 4E0R; Human HLA-A2, 3PWN; Chicken BL2*01901, 6KVM; Mouse H2-Ag7, 1F3J; Human HLA-DR1, 1AQD.

To allow convenient comparisons between similar type domains of pMHC-I and pMHC-II structures, we introduced a nomenclature denoting I-α1 and II-α1 as “pa” domains (MHC peptide binding domain a), I-α2 and II-β1 as “pb” domains, β_2_-m and II-α2 as “ia” domains (MHC IgSF domain a), and I-α3 and II-β2 as “ib” domains, respectively (**Figures 1**, **2A**). The combination of a pa and a pb domain, commonly referred to as the peptide binding domain or PBD—which would be a confusing term in the present study—is denoted as “pab” domain. Residue numbering in the present study follows the sequence alignments in **Figure 3** and **Supplementary File 1A**, and is based on the residue numbering by Saper et al. (49) for HLA-A2 α1 domain and human β_2_-m.

### Sequence Comparison of MHC-I and MHC-II Molecules

Sequence similarities are readily found between MHC-I and MHC-II, and also between the pa and pb domains, and between the ia and ib domains (**Figure 3** and **Supplementary File 1A**). Some sequence features are well conserved in both MHC-I and MHC-II, while others are characteristic for either class. A relatively large set of residues are characteristic for MHC-I.

The sequences of the representative pMHC-I and pMHC-II structures compared in **Figure 2A** are aligned per domain-type in **Figure 3** with indications of their secondary structures. In **Supplementary File 1A**, additional representative MHC sequences throughout jawed vertebrates are compared. Most parts of the sequence alignments readily agree with structural superimpositions (**Supplementary File 1B**), but in some highly variable—mostly loop—regions, the best possible alignments are not certain.

The **Figure 3** and **Supplementary File 1A** sequence alignments show that, arguably, the most dramatic consistent difference between the MHC-I and MHC-II sequences maps to the N-terminal part of the pa-domain helical region, where II-α1 domains exhibit deletions of a few residues compared to the MHC p-domain consensus situation (5). Moreover, compared to I-α2 and II-β1, the I-α1 sequences have insertions of one or few residues around position p55 plus, in most cases, deletions of a few residues around position p62b, which are coincident with the presence and absence of helix interruptions (kinks), respectively. Among the l-α3 domain sequences, length variation is observed in the S1S2-loop regions, and, in teleost fish, in the C-terminal regions [**Supplementary File 1A**(b) and see also (51)].

The color shadings of residues in **Figure 3** and **Supplementary File 1A** are based on, partially subjective, comparisons of sequences, and help to estimate when in evolution certain residues or “residue-types” were established or fixated. “Residue-types,” here, refers to the sets of residues indicated above the alignment, which were chosen based on observed MHC sequence conservation patterns [see also (12–14)], and on similarities between amino acids. In **Figure 3**, residues or residue-types that, by deduction, were probably present at the respective position in the assumed ancestral homodimer, are shaded black. Residues or residue-types that probably were present in early members of, and are characteristic for, the a lineage (I-α1+β_2_-m/IIα) and the b lineage (I-α2+I-α3/IIβ), are shaded dark blue and dark purple, respectively. Residues or residue-types that are characteristic for the I-α1+β_2_-m, IIα, I-α2+I-α3, and IIβ lineages are shaded light blue, blue, pink, and purple, respectively.

In **Supplementary File 1A**(b), also some IgSF C1 set sequences of non-MHC molecules are compared, which shows that the IgSF residues shaded black in **Figure 3** are common among IgSF C1 sequences. However, the tryptophan at position 95 is quite characteristic for MHC, and is shaded red.

Importantly, **Figure 3A** and **Supplementary File 1A**(a) show that the MHC-I pa and pb domain sequences show many more specifically conserved residues than found in MHC-II. This is reflected in the overall similarity levels per domain if comparing across wide species borders, as summarized for a set of representative sequences in **Supplementary File 1C**. For example, nurse shark UAA*01 and β_2_-m compared with human HLA-A2 and β_2_-m show the following percentages of aa identities per domain (alignment as in **Figure 3**): pa, 45%; pb, 38%; ia, 40%; ib, 27%, whereas between nurse shark MHC-II (the IIα and IIβ sequences shown in **Supplementary File 1A**) and HLA-DR1 the identity percentages are: pa, 31%; pb, 21%; ia, 44%; ib, 44% (**Supplementary File 1C**).

As for variation among MHC-I sequences, the better conservation across wide species borders of I-α1, I-α2, and β_2_-m compared to I-α3 sequences (**Supplementary File 1C**) was already noticed by Kaufman et al. (52). Furthermore, the lower levels of similarity among I-α3 domain sequences compared to among β_2_-m, II-α2 and II-β2 domain sequences (**Supplementary File 1C**) was also reflected in published phylogenetic tree analysis results [e.g., (53)]. The relatively poor conservation of I-α3 sequences probably is related to this domain participating in only few interdomain interactions and an apparent absence of stringent evolutionary pressure to conserve MHC-IgSF-typical intradomain features (see below).

### Comparison Between pMHC-I and pMHC-II Structures

#### Similarity in Overall Domain Organization but With Differences in ib Domain Orientations

**Figure 4:**
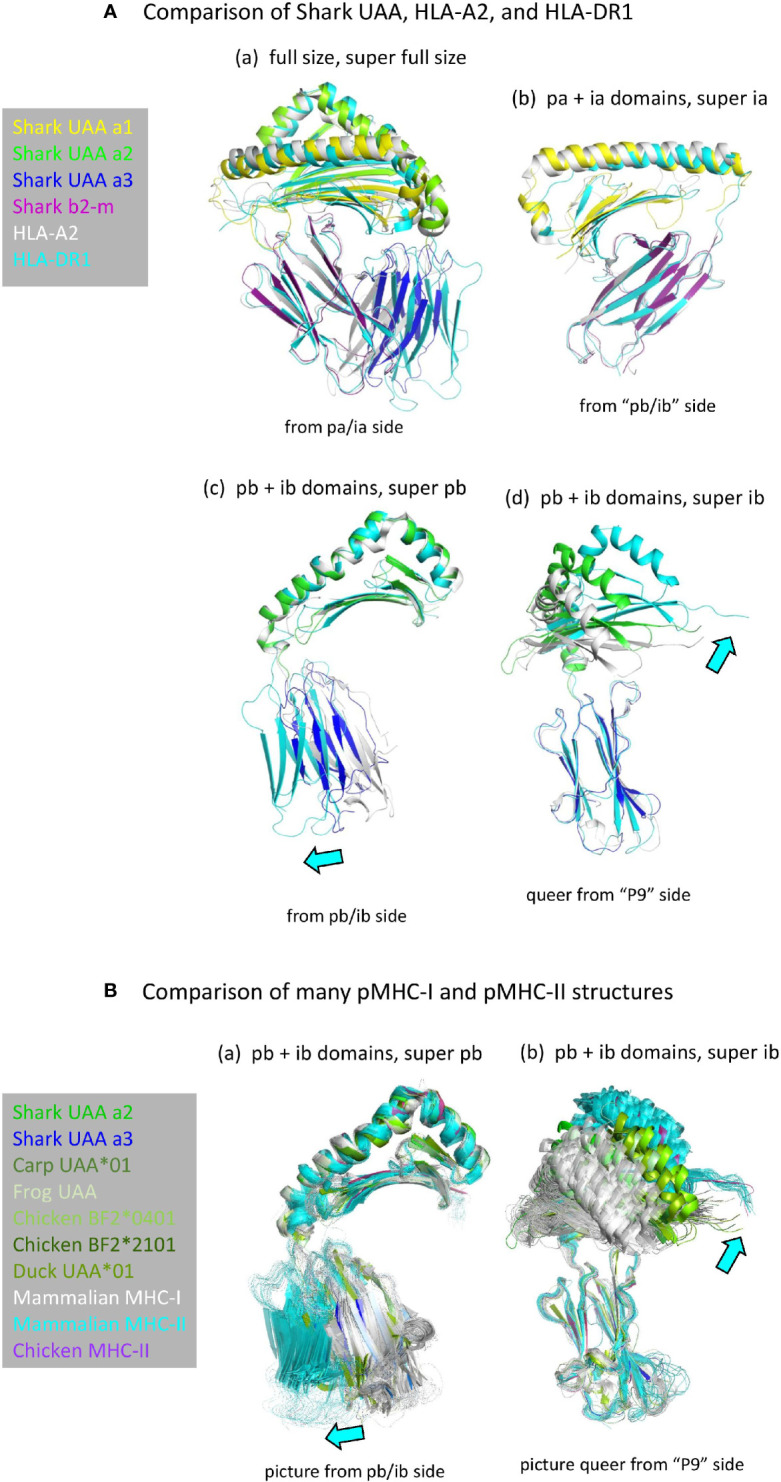
**(A)** Comparison of domain orientations in pMHC-I and pMHC-II. The description “super” relates to the super function of PyMOL software that was used for superimposing the indicated domain. **(A**a**)** Superposition of the full size pMHC ectodomain structures (shown without peptide) of shark pUAA (colored per domain), pHLA-A2 (gray), and pHLA-DR1 (cyan) reveals the largest orientation differences in the ib (I-α3 and II-β2) domains, with shark pUAA ib taking an intermediate position. By separate analysis of the pa+ia and pb+ib domains, it is easily seen that the pa+ia domain orientations are very similar between the different pMHC molecules (**A**b), whereas there are considerable differences in the ib to pb angles (**A**c, **A**d). In (**A**c, **A**d), cyan arrows highlight the directions in which respective pHLA-DR1 domains differ from the corresponding pHLA-A2 domains. **(B)** Figures (**B**a, **B**b**)** are as (**A**c, **A**d), except that they compare many more pMHC-Is (gray for mammals, green or dark blue for primitive vertebrates) and pMHC-IIs (cyan for mammals, purple for chicken), revealing an overlap between the range of ib domain orientations in pMHC-Is of primitive vertebrates and pMHC-IIs.

Structures of pMHC-I and pMHC-II are very similar (**Figure 2A**) (5). Apart from the peptide lengths, the biggest differences in their overall structures are the orientations of the ib domains (I-α3 versus II-β2; **Figure 4**) (5). The orientations of ib domains do not only differ between pMHC-I and pMHC-II, but also among pMHC-Is (28, 31) and among pMHC-IIs (54) (**Figure 4B**). Compared to α3 domains in mammalian pMHC-Is, the orientations of α3 domains in pMHC-Is of primitive jawed vertebrates are more similar to the orientations of β2 domains in pMHC-IIs [**Figures 4A**(c, d) and **4B**] and they overlap with the outer range of reported II-β2 orientations (**Figure 4B**), suggesting that they represent the more primitive orientation of I-α3 domains.

#### The pb Domain Helices Are Similarly Organized in pMHC-I and pMHC-II, and May Represent the Ancestral Helix Organization

**Figure 5:**
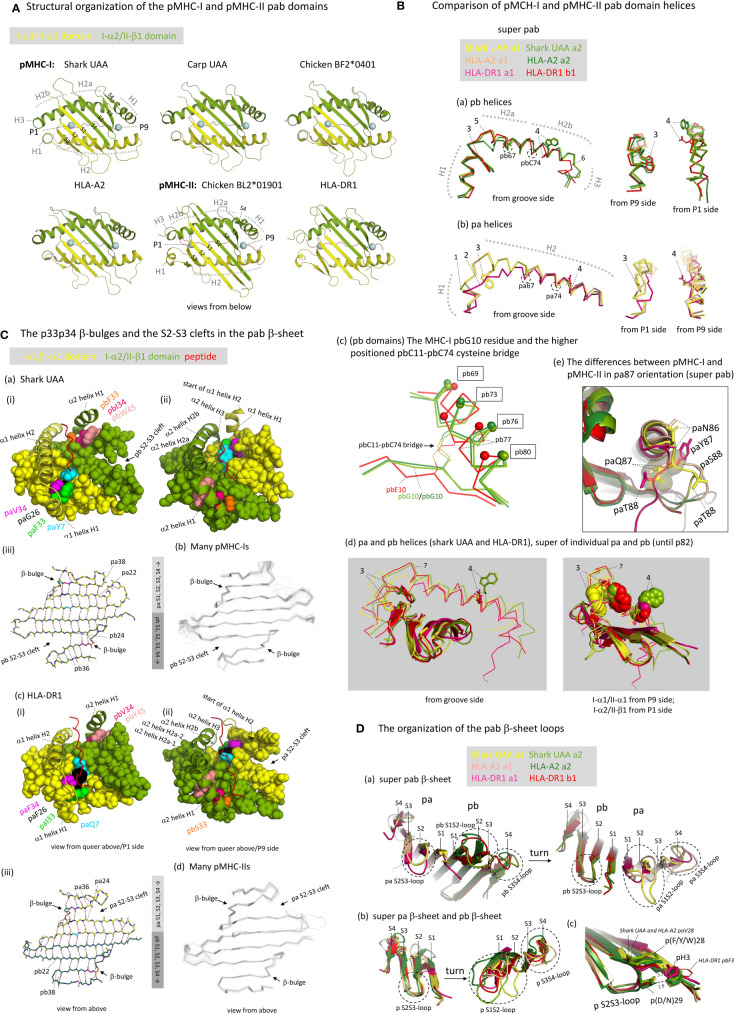
The structure of the pab domains. **(A)** Structural organization of the pMHC-I and pMHC-II pab domains, shown from underneath with indications of pa and pb domains (yellow and green), β-strands (S1-to-S4; PyMOL software does not recognize the shark pUAA α1 domain S4 strand whereas DSSP software does, see **Figure 3A**), α-helices (H1-to-H3), peptide ligand (red), and peptide ligand P1 and P9/Ω Cα atoms (blue spheres). **(B)** Comparison of pMHC-I and pMHC-II pab domain helices. (**B**a, **B**b) show superpositions of pb and pa domain helical structures, respectively, from different angles, of shark pUAA, pHLA-A2, and pHLA-DR1, based on superimposing of the pab domains, and (**B**d) shows a superposition of the helical regions of both the pa and pb domains of shark pUAA and pHLA-DR1, based on superimposing of their position 1-82 stretches. In the (**B**a, **B**b, **B**d) figures, the numbers 1-to-7 refer to: (1) The H1 helix regions of mammalian I-α1 compared to I-α1 in more primitive species lost a residue [**Supplementary File 1A**(a)] and this seems to be compensated for by having a 3_10_-helix structure in mammalian I-α1 H1 helix rather than a (more loosely winding) α-helix structure as found for some pMHC-I of primitive animals [**Figures 3A** and **5B**(b), although frog pUAA also has a 3_10_-helix at this position] (27); (2) Compared to the other classes of p domain, in II-α1 a few helical turns were replaced by a stretch of extended chain (5); (3) In I-α2 and II-β1, ancestral pW60 is part of the H1 helix and its sidechain points sideways into the groove; in I-α1, pW60 became part of the long H2 helix and points downwards to the β-sheet; in II-α1 sequences pW60 was lost [**Supplementary File 1A**(a)]; (4) In I-α1, II-α1, and II-β1, p77 residues have the ability to form hydrogen bonds with the peptide ligand backbone (see **Figures 6B, C**) and are situated at the middle height of the groove, whereas in I-α2 the ancestral p(D/N)77 residue has been replaced by a tryptophan starting at a higher position and blocking the groove [see also **Figures 6F, G**). The higher helix position of I-α2 p77 is caused by an uplifting of the pbC11-pbC74 disulfide pair caused by an absence of typical β-strand folding at the pbG10 position (**B**c); in (**B**c), to help readers with orientation, small spheres indicate the Cα of positions that are indicated with boxes; (5) The pb domain H1-H2 elbow organization is similar in shark pUAA and pHLA-DR1, and different from pHLA-A2, consistent with the number of residues in this region [**Figure 3A** and **Supplementary File 1A**(a)], but a possible functional impact is not known [see also (30)]. (6) The I-α2 domains have a steeply downwards running H3 α-helix, but the II-β1 sequences have fewer residues in this region [**Figure 3A** and **Supplementary File 1A**(a)] and breach the same distance by a less steeply orientated and less tightly wound spiraling structure. The No. 6 indicator in (**B**a) points at pMHC-I pbG85, at the border between pMHC-I pb helices H2b and H3; (7) The kink regions between helices H1 and H2 of the pb (I-α2 and II-β1) domains are at a higher point relative to the β-sheet than the corresponding regions in the pa (I-α1 and II-α1) domains. (**B**e) The differences between pMHC-I and pMHC-II in pa87 orientation are shown in a superposition of shark pUAA, pHLA-A2, and pHLA-DR1 pab domains with highlighting the pa86, pa87, and pa88 sidechains in sticks format; the shark pUAA and pHLA-A2 paQ87 sidechains and Cα atoms are additionally highlighted in transparent spheres format to show how they, different from pHLA-DR1 paY87, may be responsible for uplifting the end of the pa domain H2 helix. **(C)** The p33p34 β-bulges and the S2-S3 clefts in the pab β-sheet. In (**C**a-i, ii), (**C**c-i, ii), respectively, shark pUAA and pHLA-DR1 pab domain β-sheet residues are shown in spheres format, while peptide ligand, α1 helical region, and α2 helix H1 are shown in cartoon format. Residues of interest are given individual colors. Whereas in the α2 domain the β-bulge pb33+pb34 residues form a supportive ridge together with pb45 for the above-positioned and parallel running pb domain H1 helix, in the α1 domain the pa33+pa34 β-bulge is not positioned under the α1 domain H1 helix. In (**C**a-iii), (**C**c-i), the main chains of the pab β-sheets of shark pUAA and pHLA-DR1, respectively, are shown from above in sticks format and hydrogen bonds between them are shown by dashed red lines; residue colors match those in the pictures on the left. In pMHC-I pa and pMHC-II pb domains, the β-sheet connection between β-strands S2 and S3 extends to the p22-p38 pair, whereas pMHC-I pb and pMHC-II pa have an “S2-S3 cleft” meaning that the S2-S3 β-sheet connection only extends to the p24-p36 pair. (**C**b, **C**d), showing superpositions of many pMHC-I or pMHC-II structures, reveal that this organization is conserved among pMHC-I and pMHC-II structures, respectively. **(D)** The organization of the pab β-sheet loops. (**D**a) The pab β-sheets of shark pUAA, pHLA-A2, and pHLA-DR1 were superimposed and are shown from either side to highlight the loop orientations. (**D**b) The pa and pb domain β-sheets were also superimposed with each other. The conclusion from (**D**a, **D**b) is that the S2S3-loop orientation is well conserved, even between pa and pb domains, but that there is a lot of variation in S1S2- and S3S4-loops [see also (**C**b, **C**d)]. (**D**c) shows a fragment of the **(D**b**)** superposition with the sidechains of the p3, p28, and p29 residues highlighted in sticks or lines format, with numbered dashed gray lines indicating the distance in Å between pHLA-A2 pbH3, pbDY28, and pbD29.

To follow the curve of the β-sheet, both in pMHC-I and pMHC-II, and both in the pa and the pb domains, the α-helical regions are divided into parts separated by “kinks” (4, 5). The parts separated by kinks are commonly named H1 and H2 in the case of the pa domains, and H1, H2a, H2b, and H3 in the case of the pb domains (**Figures 3A**, **5A, B**) (49, 50). The pb domain H1 helices are longer than those in the pa domains (**Figure 3A**), and run parallel to, and above, the pb domain β-strands S3 and S4 (**Figure 5A**). After a big kink (“elbow”), this is followed by a long pb H2 helix that because of a small kink can be divided into H2a and H2b parts [**Figures 5A, B**(a)]. In pMHC-I pb, the H2b helix continues longer than in pMHC-II pb, and at the MHC-I-specific residue pbG85 the strand bends in a rather straight angle to continue as a small helix named H3 [**Figure 5B**(a)]. In pMHC-II pb, in the absence of pbG85 and having a few residues less to span the same distance as in pMHC-I, the H2b helix structure stops earlier and continues in a more tightly wound H3 helix without α-helix characteristics [**Figure 5B**(a)] (5, 50); consequently, in pMHC-II there is no sharp H2-H3 angle [**Figure 5B**(a)]. Compared to pMHC-II, the pbC11-pbC74 cysteine bridge is pushed slightly upwards in pMHC-I, causing a slight rotational change in the pb H2b helix that results in a more upward orientation of pbW77 in pMHC-I compared to the pbN77 orientation in pMHC-II that presumably represents the ancestral situation [**Figure 5B**(a), -(c), and -(d); see also below]. The higher position of pbC11-pbC74 in pMHC-I is accompanied by the evolutionary acquisition/fixation of pbG10 and a disruption of the regular β-sheet folding in the pb9-pb11 stretch, whereas regular folding is found at corresponding positions in pMHC-II pb and in the pMHC-I and pMHC-II pa domains [**Figure 5B**(c) and **Supplementary File 1B**(a)]. The pbG10-dependent β-sheet irregularity resulting in different positions/orientations of the pbC11-pbC74 pair and the pb H2b helix region had been noted before in a classical MHC-I versus MIC-A (nonclassical MHC-I) comparison context (55), but, as far as we know, not in a I-versus-II context.

We assume that, in evolution, the ancestral MHC homodimer had a H1-H2a-H2b helix organization similar to that in MHC-II β1 domain, because: (i) a similar organization is found in MHC-I α2 domain [**Figures 5A, B**(a)]; (ii) the conservative nature of the MHC-I and MHC-II pb helices is suggested by their conservation of the ancestral pC11-pC74 cysteine bridge [**Figure 5B**(c) and **Supplementary File 1A**(a)]; and, (iii) MHC-II β1 helices can bind the peptide ligand orientated in either N-to-C or C-to-N direction using their ancestral pW60 and pN77 residues (see below) (56).

#### The pa Domain Helices of pMHC-I and pMHC-II Differ From Each Other and From the Presumed Ancestral Situation

If, as we assume, the helix organization in the ancestral MHC homodimer was similar to that in extant pMHC-II pb indeed, the shared evolution of MHC-I and MHC-II pa domains probably involved the reorientation of the H1 helix from being parallel as in the pb domains to being rather perpendicular to the β-sheet as in the pa domains (**Figure 5A**). However, the MHC-I and MHC-II pa domain helix organizations also differ from each other. In I-α1 domains, compared to MHC pb domain helix organization, seemingly, insertions of residues around position p55 and deletions around position p62b deleted the H2a-H2b kink and repositioned the H1-H2 kink, and herewith shortened the H1 helix and lengthened the H2 helix [**Figures 3A**, **5B**(d), **Supplementary File 1A**(a)]; this reorganization resulted in partial rotation of the helical stretch including residues p59 and p60, so that in pMHC-I paY59 now points to the bottom of the groove (see below) and pMHC-I paW60 no longer is part of the groove but only connects the helix to the β-sheet [**Figures 5B**(b) and -(d), and see below]. Compared to I-α1, in II-α1 domains the H1-H2 elbow region was shortened by deletion of a few residues and the helices partially melted (5), while ancestral paW60 was lost [**Figures 3A**, **5B**(b) and -(d), **Supplementary File 1A**(a)]; given the diverged sequences of this MHC-II pa stretch, it can’t be determined with certainty at which precise positions the residues were deleted. Sequence comparisons suggest that the helix organization of II-α1 found in mammals and chicken is common throughout MHC-II in jawed vertebrates, although the ancestral paC11-paC74 cysteine pair that was independently lost in tetrapods and Elasmonbranchii (sharks/rays) has been retained in other clades of jawed vertebrates [**Supplementary File 1A**(a)]. The losses in several clades of animals indicate that the paC11-paC74 cysteine pair is not very important for MHC-II, and the pair has also been lost throughout MHC-I [**Supplementary File 1A**(a)]. In both pMHC-I and pMHC-II, the region including pa11 interacts with the below ia domain (β_2_-m or II-α2, respectively), and so may provide a regional stabilization causing redundancy of the stabilization by paC11-paC74; in contrast, in both pMHC-I and pMHC-II, the well-conserved pbC11-pbC74 cysteine bridge stabilizes a part of the pab domain that is free of IgSF interaction (**Supplementary File 3C**).

The lifting of the end of the C-terminal part of the pa domain helical region in pMHC-I compared to pMHC-II [already noted by Brown et al. (5)] may be explained by the pMHC-I pa domain strand bending backward for continuation as a pb domain strand, concurrently inserting the MHC-I-specific paQ87 sidechain into the pab domain bottom [**Figure 5B**(e)].

#### The pab Domain β-Sheets Show Conserved β-Bulges in Both pa and pb Domains of Both pMHC-I and pMHC-II, and Unique Differences in the Aligning of β-Strands Between pMHC-I and pMHC-II

An unusual feature shared among all investigated pa and pb domains of both pMHC-I and pMHC-II is a β-bulge at positions p33 and p34 of β-strand S3 with both sidechains pointing upwards (**Figure 5C** and **Supplementary File 3D**) (5, 49). In the ancestral MHC homodimer molecule, these p33 and p34 residues probably were hydrophobic [**Supplementary File 1A**(a)], and the best residue conservation is found among MHC-I α2 domains which tend to possess pb(F/Y)33 and pb(I/L)34 [**Figures 3A**, **5C**(a), and **Supplementary File 1A**(a)]. Together with the pb-lineage-specific aromatic residue pb(F/Y/W)45, of which the sidechain “leans over” from β-strand S4 to β-strand S3, in I-α2 and II-β1 domains the residues pb33+pb34+pb45 form an, in many instances hydrophobic, ridge on which the pb domain H1 helix rests (**Figure 5C**). The better conservation of the pb33 and pb34 residues among I-α2 than among II-β1 sequences [**Supplementary File 1A**(a)] suggests that the ridge function is under stricter requirements in pMHC-I than in pMHC-II. In the pa domains of pMHC-I and pMHC-II the β-bulge does not have the same function as in the pb domains because the H1 helices have a different orientation (**Figures 5A, C**).

**Figure 5C** and **Supplementary File 3D** also show remarkably conserved features that, to our knowledge, have not been noted before, and which we named “pb S2-S3 cleft” in pMHC-I and “pa S2-S3 cleft” in pMHC-II. These features concern that in pMHC-I pab β-sheets the α2 domain strands S2 and S3 only show main chain pairing until residues pb24 and pb36, respectively, and that beyond this contact the S3+S4 strands bend away from the rest of the β-sheet; in investigated pMHC-II structures a similar situation is found in the MHC-II α1 domains, with pa24 and pa36 as border. Meanwhile, in pMHC-I α1 and pMHC-II β1 domains the S2-to-S3 main chain pairings extend beyond the p24-p36 interaction (**Figure 5C** and **Supplementary File 3D**). The S2-S3 clefts probably increase the regional flexibility, and their positions associate with structures that undergo pronounced positional changes upon peptide binding/editing, including the pMHC-I α2 domain H1 helix (57–60) and the border region of helices H1 and H2 in pMHC-II α1 domain (61, 62).

#### Loops Connecting β-Strands S2 and S3 Show Conserved Orientations in Both pa and pb Domains of Both pMHC-I and pMHC-II, Whereas the Other pab β-Sheet Loops Can Display Considerable Variation

The S2S3-loop orientations are very well conserved in both pa and pb domains of both pMHC-I and pMHC-II [**Figure 5D**; see also the superimpositions of many pMHC-Is and pMHC-IIs in **Figures 5C**(b) and –(d), respectively]. In the MHC homodimer ancestor, though not conserved in most MHC-II pb (II-β1) domain sequences [**Supplementary File 1A**(a)], the S2S3-loop probably bound to the S1 strand of the same domain by a hydrogen bond between pH3 and p(D/E/N)29 as seen in extant pMHC-I pa (I-α1), pMHC-I pb (I-α2), and pMHC-II pa (II-α1) [**Figure 5D**(c)] (49). In case of the pb domains, the S2S3-loop tends to possess an aromatic residue at position pb28 which also participates in an interaction with residue pb3 [**Figure 5D**(c)]. Among MHC-II α1 domain sequences a variety of hydrophobic residues is found at position pa28 [**Supplementary File 1A**(a)], which in higher tetrapods including mammals and birds tends to be phenylalanine which can interact with paH3 and paD28 reminiscent of the pMHC-I pb domain situation [**Figure 5D**(c); for chicken pMHC-II see PDB 6KVM]. Meanwhile, most MHC-I α1 domain sequences possess paV28 which may affect the orientation of the important A pocket residue pbY81 with which it interacts (see below).

In contrast to the S2S3-loops, considerable variations are observed in pa and pb domain S1S2- and S3S4-loop orientations among pMHC-Is [for more details see (27)] and in pa and pb domain S1S2-loop orientations among pMHC-IIs [**Figures 5C**(b) and –(d), and 5D]. The pa and pb domain S3S4 loop orientations of pMHC-II and pMHC-I in primitive animals are similar and probably reflect the orientation within the ancestral MHC homodimer; compared to this, the S3S4 loops of mammalian pMHC-I pa (I-α1) domains are two residues longer, and the S3S4 loops of mammalian pMHC-I pa (I-α2) domains have flipped upwards [**Figures 3A**, **5C**(b) and –(d), and **5D**] [for more details see (27)].

#### In pMHC-I the Peptide Ligand P1 and P9 Positions Are Closer Together Than in pMHC-II, and the P1 Sidechain Points Upwards Instead of Downwards.

**Figure 6:**
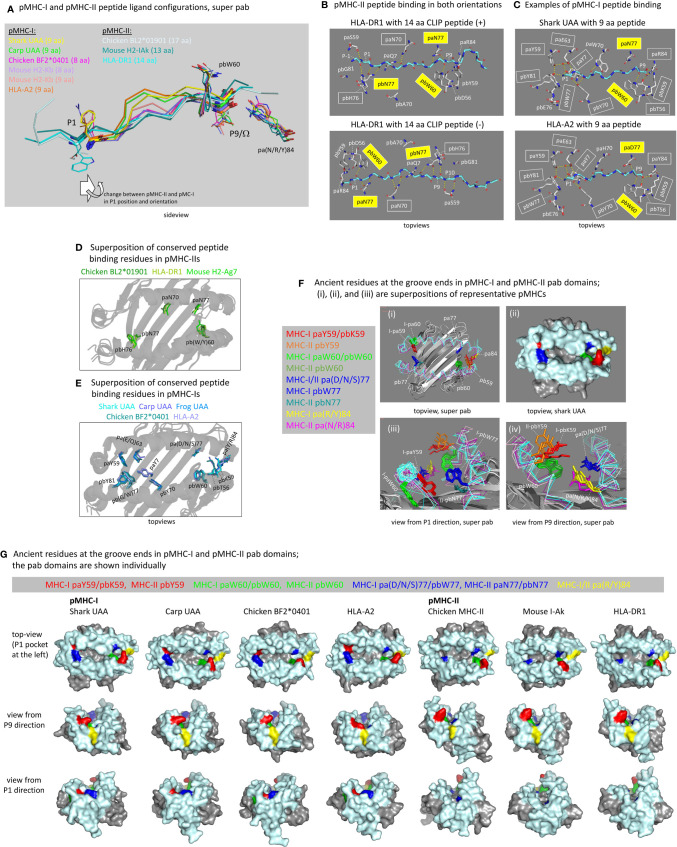
The peptide binding groove and the peptide ligand. **(A)** Comparison of peptide orientations in shark pUAA and representative pMHC-I and pMHC-II structures based on superimposing of pab domains. Peptides are shown in ribbon format, with P1 and P9/Ω main chain residues additionally shown in sticks format. P1 and P9/Ω sidechains, and pbW60 and pa(R/Y)84 residues (shown to help with orientation), are shown in lines format. In the sticks and lines formats, red and blue represent oxygen and nitrogen atoms, respectively. The important conclusion is that the P9/Ω residue is orientated similarly in pMHC-I and pMHC-II, but that compared to the presumed ancestral situation, as found in pMHC-II, the P1 residue in pMHC-I has shifted in C-terminal direction (to the right) and has rotated so that the sidechain points upwards instead of downwards. Examples of distances between Cα atoms are: pHLA-DR1 P1-P9, 25.7 Å; pHLA-DR1 P2-P9, 22.5 Å; shark pUAA P1-P9, 23.2 Å; and pHLA-A2 P1-P9, 22.1 Å. **(B)** The ancient character of the pMHC-II mode of binding is reflected in HLA-DR1 being able to bind CLIP peptides in both orientations [from the study by Günther et al. (56)]. Names of residues commonly conserved among pMHC-IIs for binding the peptide ligand main chain are boxed, and yellow boxes indicate residues presumably inherited from the ancestral MHC homodimer; some of the highlighted residues are only shown for easier comparison with the pMHC-I situation. Dashed yellow lines represent polar contacts. **(C)** This figure is in a similar style as **(B)**, but from the viewpoint of pMHC-I, showing two representative pMHC-I structures. In the P1 pocket, a water molecule, indicated with a red sphere, participates in a hydrogen bond network by making hydrogen bonds with paY7, paY59, and paE63. **(D)** Superposition of the pab domains of three representative pMHC-II structures shows the conserved orientations of the highlighted residues paN70, paN77, pbW60, pbH76, and pbN77 commonly involved in peptide ligand main chain binding **(B)**; mouse H2-Ag7 is an exception in that it has a tyrosine at the pb60 position. **(E)** is a similar figure as **(D)**, but from the viewpoint of pMHC-I and a superposition of representative pMHC-I structures. Conserved residues involved in peptide ligand main chain binding **(C)** are highlighted, and show a better conservation in the P1 pocket (left) than in the P9 pocket (right). (F-i, F-iii, F-iv) show pab domain (without peptides) superpositions of pMHC-I (Shark UAA, Carp UAA, Chicken BF2*0401, HLA-A2) and pMHC-II (Chicken BL2*01901, Mouse I-Ak, HLA-DR1) structures, and (F-ii) is a surface presentation of the shark pUAA pab orientation shown in (F-i) to make the **(F)** figure panel easier to understand; for this figure, mouse I-Ak was chosen over mouse H2-Ag7 because it possesses ancient pbW60. **(F)** highlights the residues at positions p59, p60, p77, and p84, as they are very important for the groove end and peptide binding characteristics, and they may all represent ancient features or lineage-specific deviations from ancient features; the ancestral MHC homodimer presumably possessed pW60 and p(D/N)77 and may have possessed a large hydrophilic sidechain at p59, while possibly the pa(R/Y)84 residue was ancestral to the I-α1/II-α1 lineage [**Supplementary File 1A**(a)]. **(G)** shows, in surface format with coloring of all molecules as done for shark pUAA in **(F)**, the individual pab domains that were compared by superposition in **(F)**; the view angles slightly differ per pMHC in order to maximally show their individual features. The views in **(F, G)** from P1 and P9 directions help to understand why in pMHC-Is the groove ends are closed and in pMHC-IIs they are not, although at the P9 ends the differences are rather subtle.

In contrast to pMHC-Is, in pMHC-IIs the groove ends are open, and the peptide lays in the groove in an extended, polyproline-like manner (5, 63). Although in pMHC-IIs the peptides typically are >9 aa and extend beyond the groove ends, the core interaction within the pMHC-II groove concerns a 9 aa peptide fragment as in most pMHC-Is, and the start and end residues of such fragment are named P1 and P9 (the residues N-terminal of the P1 residue are named P-1, P-2, etc.). Whereas in pMHC-I the P1 sidechain points upwards and the PΩ sidechain points downwards, in pMHC-II both the P1 and P9 sidechains point downwards (**Figure 6A**) in a semi-symmetric structure. The distances between the P1 and P9 pockets (aka A and F pockets in pMHC-I) in the groove of pMHC-II are larger than in pMHC-I (**Figure 6A**), and the linear distances between the Cα atoms of pMHC-I peptide residues P1 and PΩ (P9 in 9 aa peptides) are similar to those between pMHC-II peptide residues P2 and P9 (see **Figure 6A** legend). The different organization explains why peptide ligands of 9 aa length bulge in pMHC-Is, whereas in pMHC-IIs the peptide ligand main chains are in a regular extended configuration (**Figure 6A**). Naturally, as has been shown in multiple studies but is not shown here, in pMHC-I structures with peptides >9 aa, the bulging is more extensive (e.g., 64, 65). At a different note, the closing of the pMHC-I P1 and P9 pockets for peptide extensions beyond the groove is not absolute, but for such discussion we refer to other articles (66–69).

#### Peptide Binding in pMHC-II Is Rather Symmetric, but in pMHC-I the P1 Pocket Very Much Changed in Comparison to pMHC-II and the Presumed Homodimer Ancestor

The peptide binding mode in pMHC-II is characterized by pa-pb semi-symmetry and presumably similar to a symmetric peptide binding mode of the ancestral MHC homodimer. In pMHC-IIs, three ancient residues that make hydrogen bonds with the peptide ligand main chain are paN77, pbW60, and pbN77 (5, 12, 39, 54), and they can bind peptides in either N-to-C or C-to-N direction (**Figure 6B**) (55). The ancestral MHC homodimer presumably possessed a symmetry of pW60 and p(D/N)77 in each p domain, as specific loss of pW60 in the MHC-II pa lineage is suggested by the presence of pW60 in MHC-II pb as well as in MHC-I pa and pb [**Figure 3A** and **Supplementary File 1A**(a)]. In pMHC-Is, ancestral residues (or residue characteristics) for forming hydrogen bonds with the peptide ligand were conserved in the form of pa(D/N/S)77 and pbW60 (better known as “W147”), although their hydrogen bonding with the peptide ligand is not consistently conserved among pMHC-Is and may depend on the HC allele and the bulging of the particular peptide ligand (e.g., **Figure 6C**) (27, 30, 70). In pMHC-Is, the paW60 residue, because of reorganization of the respective pa domain helical region, can no longer participate in peptide binding [**Figures 5B**(b) and –(d), **6F, G**]. Furthermore, in pMHC-Is, the ancestral pb(D/N)77 was replaced by the large hydrophobic pbW77, which because of a rotation in the respective pb domain helical region has its sidechain pointing more upwards and blocking the groove at the P1 pocket side [**Figures 5B**(a), -(c), and –(d), **6F, G**].

Besides the ancestral paN77, pbW60, and pbN77 residues, the MHC-II lineage rather consistently possesses the two residues paN70 and pb(H/N)76 for (presumably) making hydrogen bonds with the peptide ligand main chain (**Figure 6B**) (5, 12, 39, 54); residue pbN76 is only common in MHC-II of primitive vertebrates [**Supplementary File 1A**(a)], and its possible hydrogen bonding with the peptide ligand remains to be determined. In pMHC-I, residues at positions pa70 and pb76 in pMHC-I do not possess homogeneous characteristics [**Figure 3A** and **Supplementary File 1A**(a)] and do not usually bind the peptide ligand main chain (**Figure 6C**). Therefore, and also because they are not part of an ancient pa-pb symmetry, we assume that paN70 and pb(H/N)76 were specifically acquired/fixated in the MHC-II lineage. Conserved orientations of the here listed MHC-II residues are shown in the pMHC-II superposition figure in **Figure 6D** (note that mouse H2-Ag7 possesses pbY60 instead of the typical pbW60).

The P9 pockets (aka F pocket in case of pMHC-I) are quite similar between pMHC-I and pMHC-II, but in pMHC-I a higher number of residues for peptide ligand main chain binding were acquired/fixated including pa(R/Y)84, pbT56 (aka “T143”), and pbK59 (“T146”) [**Figures 3A**, **6C** and **Supplementary File 1A**(a)] (27, 71). Explanations for why in pMHC-Is the groove is closed, while in pMHC-IIs the groove is open, are, in the case of pMHC-I, the higher and lower positions of the sidechains of the pa84 and pb59 residues, respectively (**Figures 6F, G**) (5). A mechanical explanation for the lifting of the C-terminal part of the pa domain helical region in pMHC-I compared to pMHC-II, was discussed above [**Figure 5B**(e)]. Even among pMHC-I P9 pockets there is some variation in the contribution of the conserved peptide binding residues (examples in **Figures 6C, E**) (27, 70, 72), and pMHC-I P9 pockets may need some flexibility for engulfing various amino acid sidechains.

In contrast to the P9 pockets, the P1 pockets (aka A pocket in case of pMHC-I) are very different between pMHC-I and pMHC-II. A critical difference caused by the change and reorientation of the ancestral pbN77 into pMHC-I pbW77 was explained above, and this change forced the P1 residue of the peptide ligand to bind more towards the center of the groove (**Figures 6A, F, G**). This was accompanied by another critical change, namely the binding of the P1 main chain at the bottom of the P1 pocket instead of the P1 sidechain as seen in pMHC-II. For this purpose, in MHC-I sequences a number of new residues were acquired/fixated, including paY7, paY59, pa(D/E/Q)63, pbY70 (“Y159”), and pbY81 (“Y171”) for participating in a hydrogen-bond network with the main chain of peptide ligand residue P1 [**Figures 3A**, **6C** and **Supplementary File 1A**(a)]; the tyrosines among these residues show near-absolute conservation in orientation (**Figure 6E**), which is possible because the pMHC-I P1 pocket needs little flexibility since the P1 sidechain points out of the groove (**Figure 6A**) (27, 71). For the evolutionary creation of the P1 pocket in the MHC-I lineage it was also important that residue paG26 was acquired/fixated [**Figure 3A** and **Supplementary File 1A**(a)], because the absence of a sidechain at this position allows paY7 to reach the P1 pocket [**Figure 5C**(a-i)]. It is difficult to speculate which of the unique pMHC-I P1 pocket residues and features was acquired first in evolution. In some MHC-I, the pbW77 was lost with the consequences not well understood, such as in the elucidated frog UAA structure (30) which possesses pbG77 (**Figure 6E**).

#### Although the Overall Orientation of the ia Domain Is Similar Between pMHC-I and pMHC-II, only in pMHC-I the Domain Acquired a Hydrophobic Knob That Inserts Into the pab Domain β-Sheet

**Figure 7:**
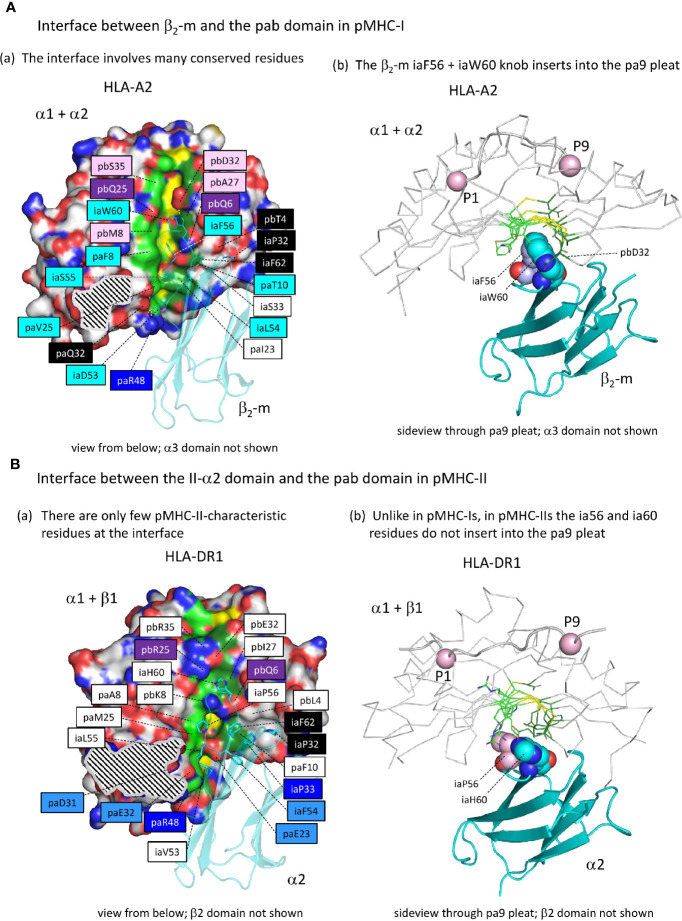
The interface between the ia and pab domains is different between pMHC-I and pMHC-II. (**A**a) The pMHC-I structures, here represented by pHLA-A2, have many conserved residues/features at the β_2_-m to α1α2 interface (for a complete list of interface residues see **Supplementary Files 2A, B**), especially at the pa9 pleat contact region. Also, a contact patch involving paT10 and pbQ6 (both part of the pa9 pleat) and pbT4 and β_2_-m ia33 (mostly iaP33) and iaF62 is relatively well conserved. Many of the indicated residue names are shaded with non-white colors, which are based on estimated conservation patterns and are also used in **Figure 3** and **Supplementary File 1A**: black, inherited from the MHC homodimer ancestor; dark blue, ancestral to the I-α1+β_2_-m/IIα lineage; purple, ancestral to the I-α2+I-α3/IIβ lineage; light blue, characteristic for the I-α1+β_2_-m lineage; pink, characteristic for the I-α2+I-α3 lineage. The α1α2 domain is indicated in surface format with only yellow for pa9 pleat top ridge residues (the ability to see them in this figure is evidence of the pleat being open) and element coloring for the other α1α2 domain residues with red, blue, and gold for O, N, and S atoms, respectively, dark and light green for the C atoms of the pa9 pleat pa8 and pa10 lower ridge residues, respectively, and white for the other C atoms. The β_2_-m domain is shown in cyan transparent cartoon format with sidechains of highlighted residues in element color sticks format. The black and white striped region is the α1α2 domain with α3 domain contact region. Residue pbS35 does not directly contact β_2_-m but pa(A/S/T)45 is a conserved part of the constellation. **(A**b) The β_2_-m iaF56 + iaW60 residues (shown in individual, element color spheres format) insert into the pa9 pleat, which can easily be seen from this angle with the α1α2 domain [coloring as in **(A)**] shown in ribbon format and the sidechains of the pa9 pleat pa8 and pa10 lower ridges in sticks format. The peptide ligand is shown in cartoon format with the Cα’s of P1 and P9 as pink spheres. (**B**a) Similar figure as (**A**a), but with pHLA-DR1 as a representative structure for pMHC-II. For interactions between the α1β1 and α2 domains, also see **Supplementary Files 2A, B**. Coloring of residue names is based on conservation patterns as also done in **Figure 3** and **Supplementary File 1A**, and is similar as done in (**A**a) except that MHC-IIα-characteristic residues are colored non-dark blue. (**B**b) is as (**A**b), but showing pHLA-DR1 as a representative pMHC-II structure. The figures **A**a-to-**B**b show that only in pMHC-I the ia56+ia60 residues penetrate into the pa9 pleat of the pab domain.

**Supplementary Files 2A, B** list all residues that according to PDBePISA software analysis are part of the ia to pab interface of several representative pMHC-I and pMHC-II structures; relevant pHLA-A2 and pHLA-DR1 residues are highlighted in the structural figures in **Figures 7A**(a), **7B**(a), with their names colored according to conservation patterns as shown in **Figure 3** and **Supplementary File 1A**. Among the, probably ancestral, interface residues shared between pMHC-I and pMHC-II are: pa(D/E/Q)32, paR48, pbQ6, pb(Q/R)25, iaP32, iaP33, and ia(F/Y)62 [also see **Supplementary File 3A**(b)]; among these residues, pa(D/E/Q)32 is better conserved in MHC-II whereas pbQ6, pb(Q/R)25 and iaP33 are better conserved in MHC-I (**Supplementary File 1A**). In contrast to pMHC-I, pMHC-II only has a few specific residues at the ia-to-pab interface [shaded non-dark blue in **Figure 7B**(a)], namely iaF54, pa(E/Q)23, pa(D/E/N)31, and paE32, the last two of which participate in the connection between the pab domain and the IgSF domains at the P1 side of the pab domain (see below). Compared to pMHC-II and, presumably, to the last common ancestor of MHC-I and MHC-II, the biggest change in the pMHC-I interdomain contacts was the acquisition of the iaF56+iaW60 hydrophobic knob and its insertion into the pab pa9 pleat [**Figure 7A** and **Supplementary File 3E**]; in pMHC-II structures, at the corresponding position, the pa9 pleat is closed by sidechains from the pleat ridge residues [**Figure 7B** and **Supplementary File 3E**]. The establishment of the iaF56+iaW60 hydrophobic knob in MHC-I was accompanied by acquisition/fixation of a large set of interacting residues that have been well conserved throughout extant MHC-I from shark to human [**Figure 3** and **Supplementary Files 1A**, **2A**, and **3A**(a)] [for more details see (27)].

#### The ib to pab Interdomain Interface in pMHC-II Acquired a Large Hydrophobic Tryptophan Residue

**Figure 8:**
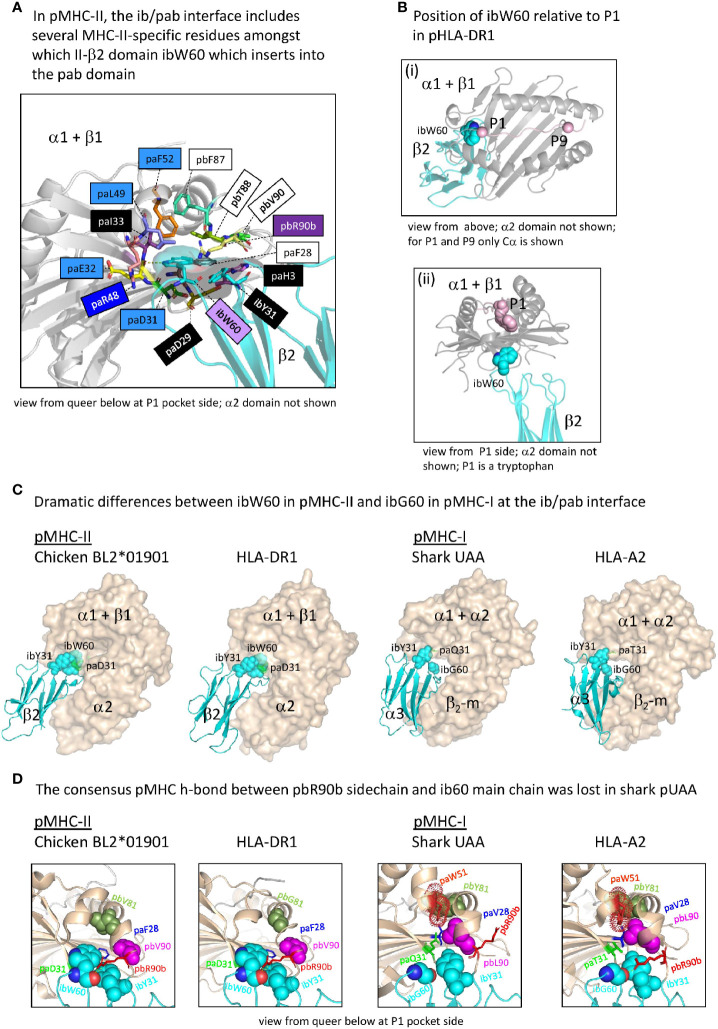
The interface between the ib and pab domains. **(A)** Shown here for pHLA-DR1, the MHC-IIβ-specific residue ibW60 residue together with the more ancient residue ibY31 forms a knob (indicated in sticks format plus transparent surface presentation) which inserts into the pab domain. Pab residues surrounding this knob, and also paH3 as being part of the paH3/paF28/paD29 interaction [**Figure 5D**(c)], are highlighted in sticks format. Residue ibW60 makes polar contacts with paD31 and paE32. Color shading of residue names, as in **Figure 7B**(a), refers to conservation patterns (see above and **Supplementary File 1A**), with light purple used for MHC-IIβ-characteristic residues. **(B**-i) and **(B**-ii) help to understand the position of pHLA-DR1 ibW60 (sidechain in spheres format) relative to the pab groove and the P1 residue. **(C)** At the position where pMHC-II possesses the very large ibW60, in pMHC-Is the very small ibG60 tends to be found (together with ibY31 indicated in spheres format). Whereas in pMHC-IIs the sidechain of a characteristic paD31 residue makes a polar contact with the ibW60 main chain, in pMHC-Is a variety of residues is found at pa31 (green). The pa+pb+ia domains are indicated in wheat color transparent surface format and the ib domains are indicated in cyan color cartoon format. **(D)** Shared between most elucidated pMHC-IIs and pMHC-Is is a polar contact between pbR90b sidechain and ib60 main chain, but in shark pUAA this contact was lost. Some other residues characteristic for this region in either pMHC-II or pMHC-I, and their counterparts in the other MHC class, are highlighted in several manners, with only ib60 coloring based on elements.

Whereas in pMHC-I an iW60 residue was acquired in the ia (β_2_-m) domain [**Figure 3B** and **Supplementary File 1A**(b)], making a large interaction with the pab domain that involves a set of additionally selected residues [**Figure 7A**(a)], in pMHC-II a reminiscent situation is observed for a specifically acquired iW60 residue in the ib (II-β2) domain (**Figure 8**) (73, 74). **Figure 8A** shows how in pHLA-DR1 the ibW60 residue inserts into the pab domain and is surrounded by multiple conserved residues including ones specifically selected in MHC-II, namely pa(D/E/N)31, paE32, pa(I/L)49, and paF52. Whereas the iaW60 residue in pMHC-I structures binds closer to the P9 side of the pab domain [**Figure 7A**(b)], the ibW60 residue in pMHC-II structures binds to the floor at the P1 end of the pab domain (**Figure 8B**) (73, 74). **Figure 8C** shows the dramatic difference in ib-pab interfaces caused by having a large ibW60 in pMHC-II β2 domains rather than a small ibG60 common among pMHC-I α3 domains. Considering the frequent finding of iG60 in non-MHC IgSF sequences [**Supplementary File 1A**(b)], we speculate that glycine represents the ancestral MHC residue at this position.

At the end of the MHC-I pb domain, residue pbL90, which is specifically conserved in MHC-I but probably also ancestral to MHC-II, forms a conserved structure with MHC-I-specific residues paV28, paW51, and pbY81 (**Figure 8D**). The nearby pbR90b residue also appears to be ancestral to both MHC-I and MHC-II [**Supplementary File 1A**(a)] and tends to strengthen the contact between the end of the pb domain and the ib domain by making one or more polar contacts with the main chain of ibG60 (in case of pMHC-I) or ibW60 (in case of pMHC-II) in most of the investigated structures (**Figure 8D** and data not shown). Shark UAA is an exception, as a residue was lost in this region [**Supplementary File 1A**(a)], and the sidechain of shark pUAA pbR90b points in a different direction (**Figure 8D**).

#### The ia to ib Interdomain Interface Is Different Between pMHC-I and pMHC-II

**Figure 9:**
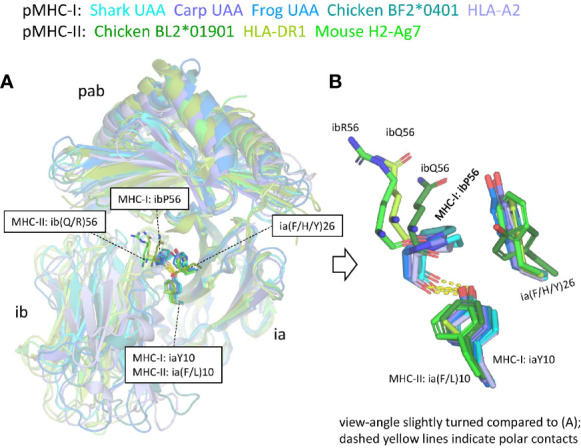
The interface between the ia and ib domains. Only among pMHC-Is a set of interacting residues iaY10, ia(F/H/Y)26, and ibP56, is well conserved. **(A)** Superposition figure (based on superimposing of ia domains) with the sidechains of residues at the ia10, ia26, and ib56 positions highlighted in sticks format. For the ib56 residues also the main chains are shown in sticks format. **(B)** Enlarged view of these residues.

**Supplementary Files 2C, D** list all residues that according to PDBePISA software analysis are part of the ia to ib domain interface of representative pMHC-I and pMHC-II structures. In both pMHC-Is and pMHC-IIs, the interaction between the ia and ib domains basically involves their S1-S2-S5-S4 (ABED) sheets, with the ia domain participating more with its S1 and S2 strands, and the ib domain participating more with its S4 and S5 strands [**Supplementary File 3B**(a)] (49). The iaY10-ibP56 bond, which is highly conserved in pMHC-I, appears to be absent in pMHC-II. Namely, (i) in MHC-II sequences ibP56 is very uncommon and iaY10 is only partially conserved and seems, judging from its conservation pattern [**Supplementary File 1A**(b)], functionally equivalent to iaF10 (bearing a sidechain without polar groups), and (ii) in elucidated pMHC-II structures, in contrast to pMHC-I, the ib56 residue is not close to the ia10 residue (**Figure 9**). On the other hand, in both chicken and mammalian pMHC-IIs, a tyrosine at position ia67 makes a hydrogen bond with the ibN57 main chain [**Supplementary File 3B**(b)] and, given the presence of these residues in MHC-II of cartilaginous fish—though iaY67 is poorly conserved in ray-finned fish [**Supplementary File 1A**(b)]—, iaY67-ibN57 may represent an ancestral pMHC-II interaction.

### The pMHC IgSF Domains Inherited Some Unique Structural Features From the Presumed Homodimer Ancestor That Are Best Conserved in pMHC-II; For Unknown Reasons, Some Consensus IgSF Features Have Not Been Well Conserved in pMHC-I

**Figure 10:**
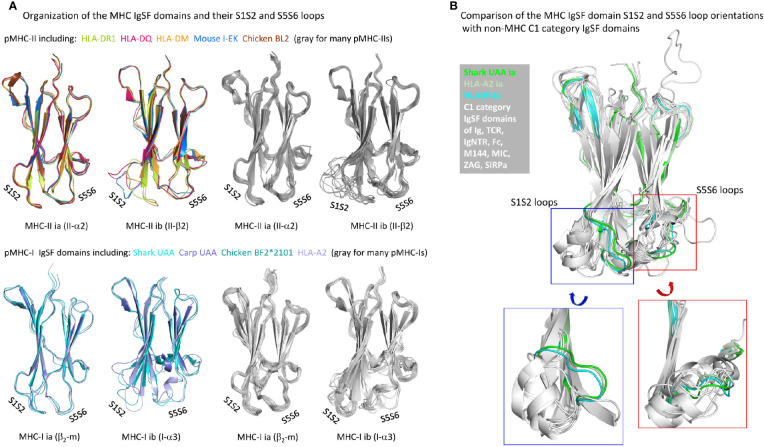
Organization of MHC IgSF domain S1S2 and S5S6 loops. **(A)** Superpositions of representative pMHC-II and pMHC-I IgSF domains (left part of the figure) and many pMHC-II and pMHC-I IgSF domains (all colored in gray; right part of the figure) show that the MHC IgSF consensus orientation of the S1S2 and S5S6 orientations are well conserved in the pMHC-II and pMHC-I ia domains; **(B)** if representative MHC IgSF domains possessing this organization are compared with a variety of non-MHC IgSF C1 set domains, it can be seen that especially their S1S2-loop orientation is quite uncommon.

The β-strands of C1 set IgSF domains of various MHC and non-MHC molecules superimpose well (75). However, considerable variation can be found in the relative orientations of their S1S2-loops and interacting S5S6-loops (**Figure 10A**), which in pMHCs are positioned distal to (away from) the pab domain. Although among the different pMHC IgSF domains the S5S6-loop orientations tend to be similar, the S1S2-loop orientation can differ a lot (**Figure 10A**). The pMHC ia domains (β_2_-m and II-α2) possess the MHC IgSF consensus orientation of the S1S2-loops (**Figure 10A**), which we assume to have been inherited from the MHC homodimer ancestor. This S1S2-loop consensus orientation is only found in approximately half of the investigated pMHC-II ib (II-β2) domain structures, while among the pMHC-I ib (I-α3) domains there is little consensus (**Figure 10A**); the variation among I-α3 S1S2-loops is also reflected in their variable lengths [**Figure 3B** and **Supplementary File 1A**(b)]. When pMHC structures representative of the MHC IgSF S1S2-loop plus S5S-loop consensus organization are compared with several non-MHC C1 set IgSF domains, it is seen that this organization is uncommon (**Figure 10B**).

**Figure 11:**
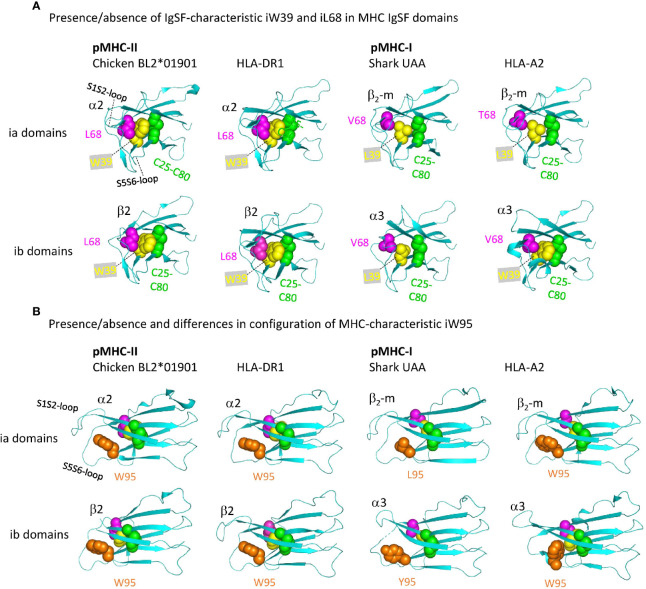
Representative MHC IgSF domains with the structurally important residues iC25-iC80 cysteine pair, iW39, iL68, and iW95, or their replacements. Highlighted residues are shown with individual colors per position and with spheres format presentation for sidechains. **(A, B)** Are from different angles. Residue coloring in **(B)** is as in **(A)**, but In **(A)** the i95 residues are not highlighted.

**Figure 11** shows important structures in the internal organization of the pMHC IgSF domains. Interestingly, compared to pMHC-II and the deduced ancestral situation, in pMHC-I evolution there have been complete or partial losses of features that probably increase the rigidity of the IgSF domains. Namely: (i) in pMHC-II α2 and β2 domains, as common among IgSF domains (76), the conserved iW39 and iL68 residues interact and form part of a topohydrophobic inner core, whereas iW39 (77) and iL68 were lost in β_2_-m, iL68 was lost in I-α3, and iW39 has not been not stringently conserved in I-α3 [**Figure 11A** and **Supplementary File 1A**(b)]; (ii) The iW95 residue and its orientation, possibly supporting the S1S2 and S5S6 loops region, are very well conserved among pMHC-II α2 and β2 domains, but not among pMHC-I α3 and β_2_-m domains [**Figure 11B** and **Supplementary Files 1A**(b), **1B**(b) last picture]. The iW95 residue is very uncommon in non-MHC IgSF molecules and probably was specifically established in the MHC homodimer ancestral molecule [**Supplementary File 1A**(b)]. The iW95 orientation in pMHC-II IgSF domains [**Figure 11B**] probably represents the ancestral situation. Experimental replacement of β_2_-m iW95 by a glycine has been reported to induce protein instability, confirming a role of iW95 in β_2_-m stabilization (78). The lack of strict conservation of the I-α3 domain structures is highlighted by the single residue shift in the main chain pairing between β-strands S6 and S7 in frog pMHC-I [last two pictures in **Supplementary File 1B**(b)].

Taken together, among the different pMHC IgSF domains, the MHC-II α2 domain appears to be the most conservative and representative of the IgSF structure in the MHC homodimer ancestor regarding the combined features of S1S2-loop orientation, IgSF-typical topohydrophobic inner core, and iW95 orientation.

#### MHC-IIα Plus MHC-IIβ Genetic Fusions in Lungfishes Suggest Another Possible Route for the Creation of MHC-I Genes From MHC-II-Like Ancestral Genes

**Figure 12:**
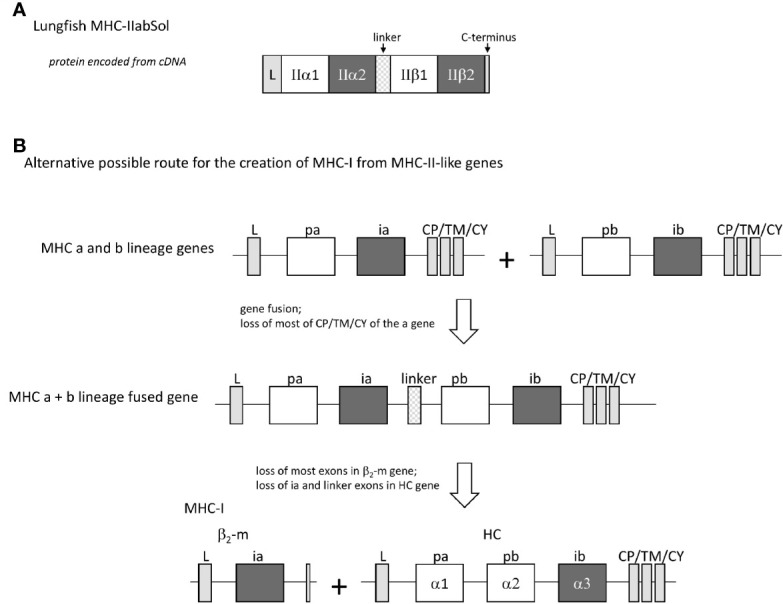
The finding of a natural genetic fusion between MHC-IIα and MHC-IIβ in lungfishes suggest the possibility of a “II-to-I” evolution route different from that proposed by Kaufman et al. (20, 21). **(A)** In lungfishes, transcripts encoding MHC-IIabSol were identified, comprising a leader sequence, MHC-II α1 and α2 domains, a linker, MHC-II β1 and β2 domains, and a short C-terminus (for sequences see **Supplementary File 4A**). **(B)** A genetic fusion combining the four MHC-IIα-like and MHC-IIβ-like ectodomain exons may have been an intermediate in the creation of MHC-I from MHC-II-like ancestral genes.

In four different lungfish species, we found transcripts encoding soluble proteins consisting of fusions of MHC-IIα and MHC-IIβ with a linker in between (**Figure 12A** and **Supplementary File 4**), which we named MHC-IIabSol. For three of the species we assembled these sequences by hand from single read archive (SRA) NCBI databases, namely for slender lungfish (*Protopterus dolloi*), West African lungfish (*Protopterus annectens*), and Australian lungfish (*Neoceratodus forsteri*). For South American lungfish (*Lepidosiren paradoxa*) sequence, a similar sequence was retrieved from an NCBI transcriptome shotgun assembly (TSA) database. For slender lungfish, we confirmed the MHC-IIabSol sequence experimentally using RNA from kidney. Indications that the gene encoding MHC-IIabSol might also produce single α or β chains, or transmembrane proteins, were not observed; however, we dedicated little effort to potentially finding alternatively spliced transcripts, and we cannot deny their possible existence. Although recombinant fusions between IIα and IIβ have been created artificially as a tool for MHC research (e.g., 79), we are not aware of previous reports on their natural existence. Considering their conservation of peptide-binding residues (**Supplementary File 4B**), the MHC-IIabSol proteins are expected to bind proteins. How a secreted pMHC-II molecule can have a biological function is unclear at this moment. The Australian and other lungfishes separated >150 million years ago (80), concluding that MHC-IIabSol is ancient. Phylogenetic tree analysis is not conclusive on how to cluster MHC-IIabSol with classical MHC-II sequences (**Supplementary File 4C**), which is a common problem when comparing proteins with different functions over large evolutionary distances (14). However, the sequences (**Supplementary File 4B**) and trees for the different domains (**Supplementary File 4C**) collectively suggest that MHC-IIabSol was established from classical MHC-II genes only in Sarcopterygii (lobe-finned fish plus tetrapods), possibly only in Dipnomorpha (lungfishes). In the context of the present paper, we are predominantly interested in MHC-IIabSol because its MHC-IIα to MHC-IIβ fusion suggests an alternative possible route for II-I evolution. Namely, rather than that a translocation event directly created an MHC-I/β_2_-m ectodomain exon organization from an MHC-II-like organization (the **Figure 1** model), an intermediate step in the creation of the MHC-I/β_2_-m system might have been an MHC-IIα/MHC-IIβ genetic fusion as speculated in **Figure 12B**.

## Discussion

The present study is the first to extensively compare structures of pMHC-I with pMHC-II. After Pamela Bjorkman as part of the Don Wiley group was the first in 1987 to elucidate a pMHC-I structure (4), hitherto, the most comprehensive analysis of pMHC-I structural organization had been the seminal study in 1991 by Saper, Bjorkman, and Wiley (49); both studies only investigated pHLA-A2. After the first pMHC-II structure was reported in 1993 by Brown et al. (5), global comparisons between pMHC-I and pMHC-II structures were made [e.g., (5, 7)], but those studies did not achieve the comprehensiveness of the Saper et al. (49) analysis, and the majority of the pMHC structural studies that followed focused on peptide-binding groove properties.

To our knowledge, other than discussion of how genes and exons may have been duplicated, differentiated, and/or shuffled [e.g., (20–23)], the early evolution of pMHC structures has hardly been discussed before. In **Supplementary File 5**, we propose a model of the major structural changes in the evolution of an ancestral MHC homodimer towards extant pMHC-I and pMHC-II. In the present study, we do not speculate on the type of homodimer molecules that may have been the evolutionary origin of the MHC homodimer molecules, and for such speculation we refer to our previous study (15).

**Figure 13:**
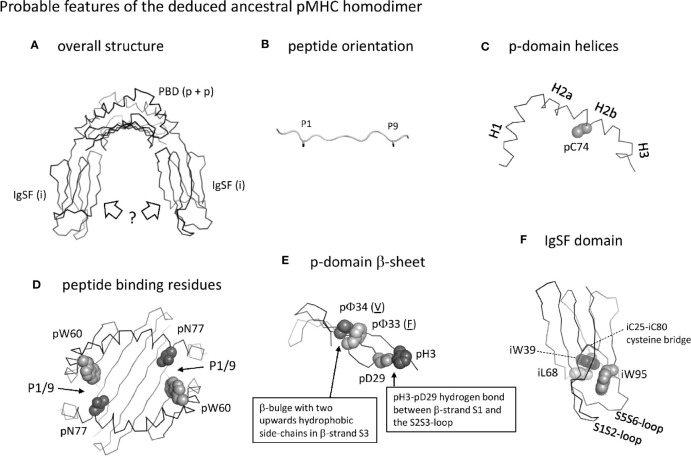
Probable features of the deduced ancestral pMHC homodimer. **(A)** Overall structure shown without peptide ligand. The peptide binding domain (PDB) consisting of two identical p domains was roughly similar to as found in extant pMHC. The orientation of the IgSF domains is very speculative, and possibly was as known for ib domains in extant pMHC (as shown in the figure) or more as structures found in antibodies (not shown). **(B)** The orientation of the peptide ligand probably was as in extant pMHC-II, with overhanging peptide ends and the sidechains of the P1 and P9 residues (shown until the Cβ atom in black sticks format) pointing downwards into the groove. **(C)** The p-domain helical regions were probably as in pb domains of extant pMHC-I and pMHC-II: helices H1, H2a, H2b, and possibly H3 were separated by kinks and formed a structure curving over the β-sheet, and a cysteine at position 74 in the H2b helix participated in a disulfide bridge with the underlying β-sheet (the β-sheet is not shown here). **(D)** Peptide binding residues. The groove was probably open at both sides allowing overhang of peptides as in extant pMHC-II, and important binding residues were pW60 and pN77 as in the pb domain of extant pMHC-II. Positions of the P1/9 pockets are indicated. **(E)** The p-domain β-sheet. As in extant pMHC, each p-domain contributed four strands of a β-sheet, with a β-bulge in β-strand S3 at positions 33 and 34 with hydrophobic sidechains pointing upward (the p33 and p34 residues shown are just examples, as indicated by their underlining). As common among pa domains in extant pMHC-I and pMHC-II, and pb domains in extant pMHC-I, there was a hydrogen bond between pH3 and pD29. **(F)** The homodimer IgSF domain presumably had a structure similar as in extant pMHC-II ia domain, with an IgSF typical core including the iC25-iC80 cysteine bridge and the hydrophobic interacting residues iW39 and iL68. The orientation of the S1S2 plus S5S6 loops was unusual compared to other C1 category IgSF domains and formed a structure together with a unique iW95 residue. For more information see **Supplementary File 5**.

The MHC homodimer ancestral state probably concerned a symmetric molecular structure (**Figure 13**, which corresponds to Stage 1 in **Supplementary File 5**). Probable features were a pab domain with an 8-stranded β-sheet, as common in extant pMHC-I and pMHC-II (**Figure 5A**), topped by two helical structures each similar to as found in extant pMHC-II pb (II-β1) domains [**Figures 4B**(a), **5B**(a)]. Characteristic features of the β-sheet were the p33+p34 β-bulge (**Figure 5C** and **Supplementary File 3D**) and the S2S3- and S3S4-loop orientations [**Figures 5C**(b) and -(d), and **5D**]. The peptide probably was bound, as in extant pMHC-II, in an extended poly-proline-like fashion, and important residues for peptide main chain binding presumably were pW60 and p(D/N)77 (**Figure 6**). Unique features of the MHC homodimer IgSF domains appear to have been their S1S2- plus S5S6-loop orientations (**Figure 10**) in combination with residue iW95 (**Figure 11**); these features locate distal from the pab domains, and, although this is speculation only, by increasing local stability they may have allowed modifications in other parts of the IgSF domains.

The homodimer ancestral gene must have duplicated and given rise to the early MHC a- and b-lineage genes (**Figure 1**) (20, 21), which together encoded an MHC-II-like heterodimer structure. Several a- and b-lineage-specific residues are rather well conserved in both MHC-I and MHC-II (**Figure 3** and **Supplementary File 1A**). The most dramatic change from the homodimer ancestral structure to the last common structure before the evolutionary separation of the MHC-I and MHC-II lineages (Stage 2 in **Supplementary File 5**) presumably was the repositioning of the IgSF domain ia domain (β_2_-m or II-α2 in extant pMHC). Bjorkman et al. (4) already noted that the asymmetrical organization of the pMHC-I IgSF domains was very distinct from a typical homodimer-like structure, and the observed, unusual, central position of the ia domain under the pab floor was later confirmed for pMHC-II (5). Given that there is no space for two IgSF domains at that central position, this cannot have been the IgSF orientation in a symmetrical ancestral MHC homodimer structure, and the IgSF domain orientations may have been more similar to those of ib domains (I-α3 and II-β2) in extant pMHC (as speculated in **Figure 13A**). The central position of the ia domain orientation may have stabilized the pab floor and so have allowed modifications of the pa domain helix organization. However, it is unclear in how far the different pa domain helix organizations in extant pMHC-I α1 and pMHC-II α1 share a common history, although they do share common differences from the presumed ancestral helix organization found in extant pMHC-II β1 domains [**Figures 3A**, **5B**(b) and –(d)]. Possibly, an increased flexibility of the pa domain helical region near the P1 pocket may have contributed to peptide ligand selectivity, as is known for extant pMHC-II in mammals (62).

Dominant changes from the time of the last common MHC-I and MHC-II ancestor towards extant MHC-II (Stage 3a in **Supplementary File 5**) presumably were: (i) the melting of part of the pa domain (II-α1 domain) helical region at the border between the H1 and H2 helices [**Figure 5B**(b)], probably in order to enhance peptide editing abilities (12, 56); (ii) acquisition of residue ibW60 in the MHC-II β2 domain [**Supplementary File 1A**(b)] for insertion into the pab domain near the P1 pocket (**Figure 8**) (73, 74), possibly for strengthening the local pab floor; (iii) the acquisition of peptide binding groove residues paN70 in the II-α1 domain and pb(H/N)76 in the II-β1 domain for binding the peptide ligand main chain (**Figure 6B**), possibly to compensate for the loss of the ancestral pW60 in the II-α1 domain (**Figures 3A**, **6F** and **Supplementary File 1A**); and (iv) —although this remains to be determined for amphibians and fishes—an “S2-S3 cleft” in the pa part of the pab domain β-sheet, possibly for increasing the flexibility of the P1 pocket region (**Figure 5C** and **Supplementary File 3D**). Sequence comparisons indicate that the change of the P1 pocket pa helical region together with the probable strengthening of the P1 pocket floor by ibW60 is shared among MHC-II throughout jawed vertebrate species (**Supplementary File 1A**), despite that nonclassical MHC-II lineage DM, which binds to the classical pMHC-II P1 pocket pa helical wall and so participates in peptide editing (62), has only been found from the level of Rhipidistia (Dipnomorpha plus tetrapods) (15). It has been speculated that in more primitive jawed vertebrates, DM-like peptide editing function may be executed by classical MHC-II in higher order complexes (12, 15).

Pronounced changes did occur from the last common ancestor with MHC-II towards extant MHC-I (Stage 3b in **Supplementary File 5**). We propose that the major changes for creating extant MHC-I were: (i) Establishment of covalent pa+pb+ib+CP/TM/CY (HC) and free ia (β_2_-m) by a process involving exon shuffling events, possibly by a route as proposed in **Figure 1** (20, 21) or as in **Figure 5**; (ii) Creation of a closed pab groove (**Figures 5B** and **6F, G**) with a new P1 pocket closer to the pab center (**Figures 5A**, **6**) and the acquisition of new/additional P1 and P9 pocket residues [**Figures 3A**, **6** and **Supplementary File 1A**(a)] (iii) Establishment of three unique interdomain interactions involving the ia domain residues iaY10, iaD53, and iaF56+iaW60 (**Figures 7**, **9** and **Supplementary Files 1**, **3A, B, E**); and (iv), an “S2-S3 cleft” in the pb part of the pab domain β-sheet which may increase the flexibility of the P9 pocket region (**Figure 5C** and **Supplementary File 3D**). As for the third point, especially the insertion of the hydrophobic iaF56+iaW60 knob into the pa9 pleat of the pab domain made a big structural difference and, in evolution, was accompanied by the acquisition/fixation of a large set of interacting residues [**Figure 7A**(a) and **Supplementary Files 1A**, **2A, B**, **3A**]. In our joint study we provided experimental evidence by alanine exchanges showing that all four β_2_-m residues iaY10, iaD53, iaF56, and iaW60 are important for pMHC-I complex formation (27), whereas previous studies only showed this for iaD53 and iaW60 (78, 81).

Comparison of sequences and structures suggest that the ia and ib IgSF domains in pMHC-I lost some rigidity compared to these domains in pMHC-II and in the presumed ancestral MHC homodimer (**Figure 11**). This may be related to a single β_2_-m molecule having to be able to bind a variety of different I-α3 domains of classical and nonclassical MHC-I (e.g., 17), or with an induced fit mechanism. However, as for the latter, the only consistent structural difference of free β_2_-m versus β_2_-m within pMHC-I complexes, analyzed for a variety of species, appears to concern that the orientations of the iaF56 and iaW60 residues in free β_2_-m are different from that in pMHC-I; however, although their orientations are similar among pMHC-Is, they differ between free β_2_-m of different species, making a more detailed discussion difficult (27).

MHC-II appear to represent the older MHC organization in regard to molecular structure, intron-exon organization, and peptide binding. Nevertheless, overall, the pa and pb domain sequences of MHC-I are better conserved in long term evolution, including ancestral residues that predate the separation of the MHC-I and MHC-II lineages (**Supplementary Files 1A, C**). This probably relates to the requirements for MHC-I function being more stringent than for MHC-II function.

A big question is why both MHC-I and MHC-II evolved and were both maintained in most jawed vertebrate species. Extant pMHC-II may have functions quite similar to the ancestral MHC homodimer, although with an improved capacity for peptide editing. On the other hand, in pMHC-I a substantially different peptide binding mode was created, with closed grooves, a shorter distance of P1-P9 binding, and a soluble ia (β_2_-m) domain. Probably, similar to the evolution of the MHC-II peptide editing system (12, 15, 56, 82, 83), the HC+β_2_-m system was developed to more strictly select only a subset of possible peptide ligands. Laws of thermodynamics conclude that synergistic binding of peptides to HC and its non-stably associated heterodimer partner β_2_-m (84, 85) should select for higher affinity peptides than in an imaginary situation with a similar but stable peptide-receptive MHC (e.g., imaginary covalent HC+β_2_-m) complex (three non-stably associated units have a stronger tendency to dissociate than two). Furthermore, closing of the grooves at the P1 and P9 pocket ends in MHC-I, compared to an open groove in the MHC-II-like ancestral situation, caused a major restriction to the lengths of peptides that could be bound. Additionally, the MHC-I groove acquired unfavorable properties for a stretched polyproline-type peptide conformation of most peptide ligands, resulting in the bulging of these peptides in individual ways (**Figure 6A**) (e.g., 70, 72, 86). Such individual bulging can structurally amplify peptide ligand sequence differences (87) and in case of tumor cells that probably makes it easier for T cells to distinguish between pMHC-I complexes with peptide ligands that differ in only single residues, even if the sidechains of those residue are not exposed at the pMHC-I surface. Considering these arguments, we speculate that the pMHC-I peptide binding mode has been developed to structurally maximize small sequence differences in peptide ligands so that it is better equipped to also recognize tumor antigens. In addition, the increased selectivity for peptide ends by having a closed groove such as in MHC-I, may have improved the ability of the immune system to distinguish between peptides generated by the different peptidase activities of proteasomes versus immunoproteasomes (88), and thus between homeostasis and inflammation conditions. After MHC-I was established, it has been superior compared to MHC-II as a source for creating a wide array of divergent nonclassical MHC molecules (e.g., 25, 89), which might be explained by the covalent association of the pa and pb domains providing a stabler situation for making functional changes. Given this superior plasticity of MHC-I, it is puzzling why most species still have MHC-II. We speculate that in unstable/variable endocytic/phagocytic compartments, where classical MHC-II molecules are loaded with peptides, the seemingly simple and robust classical MHC-II loading system (90) has advantages over the classical MHC-I loading system which—at least for the initial loading with peptide—requires free β_2_-m and multiple other factors (2). It probably would be difficult to deliver the different molecules of the MHC-I loading system in proper stoichiometry to the variable endocytic/phagocytic compartments, and the system might also be susceptible to the biochemical variations in those compartments. That the classical MHC-I loading system in mammals and fishes is similar is suggested by the conservation of relevant genes (91–93).

It has been argued that the presence of classical MHC-I genes on human Chr. 6 and of a nonclassical MHC-I gene (encoding FcRn) on human Chr. 19 suggest that MHC-I is older than MHC-II (26). This hypothesis is based on a model, from a time before whole genome sequences were known, that these human chromosomal regions are paralogues deriving from a whole genome duplication very early in vertebrate evolution (94). However, previously, we have shown that the corresponding genomic regions are linked in teleost fishes, so that many similarities between the regions may have derived from tandem gene duplications and intrachromosomal rearrangements occurring before the separation between Actinopterygii (ray-finned-fishes) and Sarcopterygii, which then in the tetrapod lineage were separated by an interchromosomal translocation event (95). Moreover, the FcRn lineage probably only separated from the classical MHC-I lineage within tetrapod evolution (14) and, in our opinion, FcRn is not a candidate for representing MHC molecules that predated the I-II divide.

With the finding of the natural MHC IIα-IIβ fusions in lungfish, which we designated MHC-IIabSol, the present study provides evidence that nature allows structures other than—complete or partial—consensus MHC-I or MHC-II organizations. Although the MHC-IIabSol sequences are very different from the classical MHC-II sequences in lungfishes, they probably were established from duplicates of classical MHC-II genes within Sarcopterygii or even within Dipnomorpha (lungfishes) (**Supplementary File 4**). Thus, these fusions represent a principle of how MHC-II exons can be combined for encoding larger structures, although we do not propose lungfish MHC-IIabSol itself to belong to an MHC lineage that predated the I-II divide. Whether the exon shuffling events for the evolutionary creation of MHC-I genes from an MHC-II-like origin were similar to the original model proposed by Kaufman et al. (20) (**Figure 1**) or involved a IIα-IIβ fusion as hypothesized in our **Figure 12** model, can’t be determined.

In short, the present study provides structural and genetic support for the II-I model of MHC evolution, identified critical evolutionary events, and recognized a large set of characteristic features— some of which for the first time—in especially pMHC-I. Future studies should use these insights to further unravel MHC functions and mechanisms.

## Data Availability Statement

The original contributions presented in the study are included in the article/**Supplementary Material**. Further inquiries can be directed to the corresponding authors.

## Author Contributions

YW and NZ contributed equally to the manuscript. They made figures and were involved in the generation of the concepts and checking the manuscript. KH and CX were involved in the generation of the concepts and checking the manuscript. CX was responsible for the generation of many of the primary data compared in this study. JD generated most of the concepts, made most of the figures, and wrote the manuscript. All authors contributed to the article and approved the submitted version.

## Funding

This work was supported by the 973 Project of the China Ministry of Science and Technology, Grant Number 2013CB835302, by the National Natural Science Foundation of China (NSFC) and by the Japan Society for the Promotion of Science Grants-in-Aid for Scientific Research, Grant Number JP26440201. The funders had no role in study design, data collection, or interpretation.

## Conflict of Interest

The authors declare that the research was conducted in the absence of any commercial or financial relationships that could be construed as a potential conflict of interest.
