# Supplementary material for: Structural Comparison Between MHC Classes I and II; in Evolution, a Class-II-Like Molecule Probably Came First
Source: Front Immunol. 2021 Jun 14;12:621153. doi: 10.3389/fimmu.2021.621153 (PMC8236899; doi:10.3389/fimmu.2021.621153)
Supplement: Supplementary file 1 [file DataSheet_1.zip › Supplementary File 1.pdf]

# Supplementary file 1

Alignment of MHC sequences

**Table of Contents** **Page**


| | |
|---|---|
| Legend to Supplementary files 1A, 1B, and 1C | 2 |
| 1Aa: Alignment of deduced MHC-I and MHC-II peptide binding domain amino acid sequences | 6 |
| 1Ab: Alignment of deduced MHC-I and MHC-II IgSF domain amino acid sequences | 7 |
| 1Ba: Superposition of the peptide-binding domains of structures whose sequences are aligned in main text Fig. 3A | 8 |
| 1Bb: Superposition of the IgSF domains of structures whose sequences are aligned in main text Fig. 3B | 9 |
| 1C: Amino acid identity percentages per identical domain between representative MHC-I and MHC-II sequences | 10 |

## Legend to Supplementary files 1A, 1B, and 1C

**(1A)** Sequence alignment of representative MHC-I and MHC-II sequences. (a), pa and pb domains; (b), ia and ib domains. Structural elements are only indicated, below the respective sequences, for HLA-A2 and human $\beta_2$-m (based on PDB accession 3PWN) (s for β-strand, h for helix). The residue numbering used in the present paper for all peptide binding domains and IgSF domains is as indicated above and below the alignments, following the numbering by Saper *et al.* (1991) of HLA-A2 α1 and $\beta_2$-m domain residues, respectively. For convenience of the readers, below the alignments also the continued numbering by Saper *et al.* (1991) of HLA-A2 α2 and α3 domain residues is shown. For database accessions of the individual sequences see the end of this figure legend.

Residues in are color-shaded if they display an interesting conservation pattern. The color-shading is based on the II-to-I evolution model and our, partially subjective, comparisons of sequences in the present and previous studies (e.g. Dijkstra et al. 2013; Grimholt et al. 2015; Dijkstra et al. 2018b), and intends to help with the estimation of when in evolution certain residues or "residue-types" were established or fixated. "Residue-types" here refers to the sets of residues indicated above the alignment, which we chose somewhat arbitrarily based on the observed MHC sequence conservation patterns and on similarities between amino acids. Residues or residue-types that, by deduction, were probably present at the respective position in the assumed ancestral homodimer are shaded black. Residues or residue-types that probably were present in early members of, and characteristic for, lineage I-α1+$\beta_2$-m/IIα and lineage I-α2+I-α3/IIβ, are shaded dark blue and dark purple, respectively. Residues or residue-types that are characteristic for the I-α1+$\beta_2$-m, IIα, I-α2+I-α3, and IIβ lineages are shaded light blue, blue, pink, and purple, respectively. In (1Ab), also some IgSF C1 category sequences of non-MHC sequences are compared, showing that the MHC IgSF residues shaded black are not uncommon among IgSF C1 set sequences. However, the tryptophan at position 95 is characteristic for MHC, and is shaded red. Fonts of residues that are not color-shaded are colored red for basic residues, blue for acidic residues, purple for cysteines, and green residues are more hydrophilic than the orange ones (Hopp and Woods, 1981). For editorial reasons, the discussion in the present study mainly focuses on those residues with interesting conservation patterns for which the functions can be known or estimated.

**(1B)** Superimposition of the structures compared in main text Fig. 3 with some residue positions highlighted by small spheres for orientation. (a), pa and pb domains; (b), ia and ib domains. The first figures in (a) and (b) show superpositions of the entire domains, and the other figures show parts of the superpositions shown in that first figure. Structures are shown from different angles for easier understanding. The N-to-C direction is shown by arrows if the direction does not follow from the positions of highlighted residue numbers. The p90 residues are situated far apart and therefore have more than one label in the same figure. In

the last figure in (a) the pbC11-pbC74 cysteine bridge is shown. In the last figure in (b), it is seen that the frog UAA α3 domain S7 strand is shifted out of MHC IgSF consensus registry.

**(1C)** Amino acid identity percentages per identical domain between representative MHC-I and MHC-II sequences. Calculations were based on the (1A) alignment figure, and indels were included as part of the calculation. Colors help to see similarity levels at a glance: yellow, $\leq$30% aa identity; light orange, 30-40% aa identity; orange, 40-50% aa identity; dark orange, >50% aa identity. At far phylogenetic distances, similarities are especially low among MHC-II α1, MHC-II β1, and MHC-I α3 sequences. For GenBank accession numbers see below.

***GenBank accession numbers of sequences compared in (1A) and (1C)***

*(Latin names of species are only given once)*

**MHC-IIA:** Nurse shark (*Ginglymostoma cirratum*), pSα5-1, GenBank M89950; Little skate (*Leucoraja erinacea*), assembly of GenBank FL670178 and CO050802; Elephant shark (*Callorhinchus milii*), AFM88468; Elephant shark, AFM88844; Bichir (*Polypterus senegalus*), assembly of SRA reads from set SRX796491; Reedfish (*Erpetoichthys calabaricus*), XP_028681425; White sturgeon (*Acipenser transmontanus*), DR975335; Zebrafish (*Danio rerio*), a4, NM_131490; Trout (*Oncorhynchus mykiss*), DAA*0101, CAB96450; Stickleback (*Gasterosteus aculeatus*), DAA*01, AY713945 and reference [10] Dijkstra et al. 2013 for correction; Coelacanth (*Latimeria chalumnae*), XP_006014228; South American lungfish (*Lepidosiren paradoxa*), GEHZ01055957; Giant salamander (*Andrias davidianus*), DAA*0109; African clawed frog (*Xenopus laevis*), DAAf1, AF454374; Gekko (*Gekko japonicus*), XP_015278405; Chicken (*Gallus gallus*), B-LA, AY357253; Opossum (*Monodelphis domestica*), XP_007483702; Mouse (*Mus musculus*), H2-Ag7, 1F3J; Human (*Homo sapiens*), HLA-DR1, HLA-DRA1, NP_061984; Human, HLA-DQ1, HLA-DQA1, M20431.

**MHC-I:** Nurse shark, UAA1, AF220063; Banded houndshark (*Triakis scyllia*), UAA*101, AF034316; Banded houndshark, UAA*201, AF034335; Spiny dogfish (*Squalus acanthias*), UAA*01, AY150811; Clearnose skate (*Raja eglanteria*), KC335152; Clearnose skate, KC335153; Guitarfish (*Rhinobatos productus*), KC469286; Elephant shark, JX207562; Elephant shark, JW872927; Whale shark (*Rhincodon typus*), XP_020366168; Elephant shark, XP_007908419; Bichir, assembly of SRA reads of Bioprojects PRJNA230234, PRJNA299881 and PRJNA269317; Reedfish, XP_028647472; Paddlefish (*Polyodon spathula*), UBA*01, GQ485566; Grass carp (*Ctenopharyngodon idella*), UAA*106, 5CNZ; Trout, UBA*0103, DQ091771; Medaka (*Oryzias latipes*), UAA*0101, BAD93265;

Coelacanth, LC2 [xx], XP_014346879; West African lungfish (*Protopterus annectens*), assembly of SRA reads of Bioprojects PRJNA164839 and PRJNA282925; South American lungfish, GEHZ01089118; African clawed frog, UAAg, AF185579; Axolotl (*Ambystoma mexicanum*), Amme3, U83137; Anole lizard (*Anolis carolinensis*), XP_016847220; Chicken, BF2*0401, AM282699; Chicken, BF2*2101, AY234769; Echidna (*Tachyglossus aculeatus*), Taac2-2, AY112713; Platypus (*Ornithorhynchus anatinus*), XP_028909408; Platypus, XP_028909721; Opossum, UC, NM_001079819; Tasmanian devil (*Sarcophilus harrisii*), I*11, EF591099; Mouse, H2-Db, MH578170; Human, HLA-C*0702, AJ001977; Human, HLA-B*2705, P03989; Human, HLA-A*02, P01892.

**MHC IIB:** Nurse shark, clone 11, L20274; Ray (*Torpedo californica*), EW694773; Elephant shark, JW875734; Elephant shark, JW875394; Bichir, assembly of SRA reads from set SRX796491; Reedfish, XP_028681436; Paddlefish, DAB*01, GU130291; Trout, DAB*1602, AF115531; Zebrafish, DAB1*01, L04805; Stickleback, DAB*01, AY713945; Coelacanth, XP_006014459; South American lungfish, GEHZ01038869; Giant salamander, KF611873; African clawed frog, DAB, D50035; Chicken, B-LBII, M29763; Mouse, H2-Ag7, H2-Ab1, M15848; Human, HLA-DR1, HLA-DRB1*010101, AM493435; Human, HLA-DQ1, HLA-DQB1*05011, AY375865.

**$\beta_2$-m:** Nurse shark, $\beta_2$-m, HM625831; Banded houndshark, $\beta_2$-m, HQ630063; Sandbar shark (*Carcharhinus plumbeus*), $\beta_2$-m, GQ865622; Spiny dogfish (*Squalus acanthias*), $\beta_2$-m, EE721262; Little skate (*Leucoraja erinacea*), $\beta_2$-m, FF600958; Clearnose skate (*Raja eglanteria*), $\beta_2$-m, AF520476; Elephant shark, ), $\beta_2$-m, JW878642; Bichir, assembly of SRA reads from set SRX796491; Siberian sturgeon (*Acipenser baeri*), $\beta_2$-m, AJ132766; Common carp (*Cyprinius carpio*), $\beta_2$-m; Grass carp, $\beta_2$-m, MG902922; Channel catfish (*Ictalurus punctatus*), $\beta_2$-m, NM_001200072; Zebrafish, $\beta_2$-m (on Chr. 4), BC162420; zebrafish, $\beta_2$-m, BC057435; Medaka (*Oryzias latipes*), $\beta_2$-m, AB006593; Medaka, $\beta_2$-m, NC_019881; Indonesian coelacanth (*Latimeria menadoensis*), $\beta_2$-m, GAPS01030276; West African lungfish, $\beta_2$-m, assembly of SRA reads of Bioprojects PRJNA164839 and PRJNA282925; South American lungfish, GEHZ01018268; Giant salamander$\beta_2$-m, KF611890; African clawed frog, $\beta_2$-m, NM_001088531; Anole lizard, XP_003227530; Chicken, $\beta_2$-m, NM_001001750; Platypus, XP_007659874; Opossum, $\beta_2$-m, NM_001032984; Mouse, $\beta_2$-m, NM_009735; Human, $\beta_2$-m, AK315776.

## References used in this file

Dijkstra JM, Grimholt U, Leong J, Koop BF, Hashimoto K (2013) Comprehensive analysis of MHC class II genes in teleost fish genomes reveals dispensability of the peptide-loading DM system in a large part of vertebrates. BMC Evol Biol 13:260.

Dijkstra JM, Yamaguchi T, Grimholt U (2018) Conservation of sequence motifs suggests that the nonclassical MHC class I lineages CD1/PROCR and UT were established before the emergence of tetrapod species. Immunogenetics 70(7):459-476.

Grimholt U, Tsukamoto K, Azuma T, Leong J, Koop BF, Dijkstra JM (2015) A comprehensive analysis of teleost MHC class I sequences BMC Evol Biol 15:32.

Saper MA, Bjorkman PJ, Wiley DC (1991) Refined structure of the human histocompatibility antigen HLA-A2 at 2.6 A resolution. J Mol Biol 219:277-319.

# Supplementary file 1A(a)

Alignment of deduced MHC-I and MHC-II peptide binding domain amino acid sequences

```
Probable ancestral homodimer residues
                               N  Q    N                           N          R
   t                           E  E   YE                           E          Q         NL
G Hs       C                   D  DΦΦ FD             w            WD          K      C  DI
II-a1/I-a1 lineage ancestral residues
                                                                                                            t  t
                      P        D                  R                                            rsn s
MHC-II alpha 1 domain
                                 N
                                 E                                              N
                         Q       DE                LP F                         D
                            .1        .10         .20E       .30   D  .40      I.50           .60           .70        .80       .90
Nurse shark pSa5-1          YLYDF-TQVYFVQQ-RS-----PEKHFDVMEDGDEIFYMDFN--LKKEVARIPEFAH-----LY-MQG-----GEAGISANI-AIVKNNLKVVMNLSGGT-PEPK
Little skate FL670178       YRHEE-HLVYLVQD-GS-----PDKQFDMLVDDDEVFYMDFK--LKKEVARIPAFQH-----IT-IEG-----GEAGITADI-AFAKQNFNVWKNLSHGS-PEPK
Elephant shark AFM88468     YVHDG-SLVWLCQD-EA-----PDKQFDVEVDWDEVYYMDFQ--QKKEIVRLPEFNS-----RT-IQG-----GEAGVSAQI-ATCKHNLGGWKKISHGDTPQPN
Elephant shark AFM88844     YVHDG-SMVSLCQD-EA-----PDKQFDIEMDWDEVLYMDFE--QKKEIVRIPHFDG-----HG-IQG-----GEASVSANI-AVCKHNLGGWKKNSQGVIPEPN
Bichir SRAs                 EPHID-INVITCIS-GS-----TETEDEEQVDGEEMFYTDFI--NNKMVITLPNFADPF---DV-VPG-----WVQTAQANK-QICLSNLDVAIKAEQNP-PEYE
Reedfish XP_028681425       EPHIE-MRVTMCTS-GS-----TETEDELQLDGDEELYADFI--NNKVVMTIPNFVDQF---GA-DPG-----WMQVAQADK-QVCFNDLGINIKAEKNP-PESK
White sturgeon DR975335     VTHLF-RALVACQT-NG-----TLPEDDYELDDDELFHIDFD--KKEAVQRIPDFAKYW---SP-PEG-----APARAETDR-QTCINNVAVAAKCHKYP-PERQ
Zebrafish a4                VVHED-IVMDGCSD-T-------EKEYISVLDGEEMYHTDFS--GKRGEMTLPDFADP----FT-YPG-----TYEQSLADY-ETCKHNLDVAAKAYKSP-LEKL
Trout DAA*0101              VLHTD-IYINGCSD-S-------DGVDMYGLDGEELWYADFN--KKEGVVALPPFADQ----IS-FPG-----YYEQAVGNQ-RTCKGDLGVDIKAYKNP-PETI
Stickleback DAA*01          GPHRD-IAITGCSD-S-------DGEDMYGLDGEELWYADFK--HGKGVKPQPSFVDPI---EF-QEG-----TYELAVGNQ-QICRINLKNRLKGLKDV-PLEK
Coelacanth XP_006014228     TSLLY-DKTLVCQT-AE-----PKVQFNWEIDEDELVRVDID--KQKMEWRLPEFKG-----HK-IDS-----LVEWTRQNI-PICEHNLDLLIKRTNGL-LGKT
S.A. Lungfish GEHZ01055957  VEHVMMAGTMFCQK-DVR----MPGQYSFEWDKEDILDVDLQ--KKESVYHAPNIQK-----FA-YYE------AQGGLQNM-AVCKHNLDVLI-RTNST-PGPT
Giant salamander DAA*0109   FDHVE-SQAIFVQT-QK-----PTGEYIFEYDKDELFHVDAD--RKEAEWRNPAFKD-----FP-TVD------IQGALGNF-AVLKTNLEISMKRSNNT-PATN
African clawed frog DAAf1   VDYFD-YGAMFYQS-YG-----PSGEYLFDYEGNEMFHVDLE--SKSVVWTLPGLEK-----YT-SYD------PQGGLQNI-NVAKYNLDGYKKSRNST-AATS
Gekko XP_015278405          AEEVL-SQVAFVQR-SLT-SEKGPGEFMFDFDGDEIFHVDLD--RKETVWRLPQFQE-----FT-SFQ------AEGAQGNI-AVLRSNLDILMRRSNNT-PAKN
Chicken B-LA                KPHVL-LQAEFYQR-SEG-PDKAWAQFGFHFDADELFHVELD--AAQTVWRLPEFGR-----FA-SFE------AQGALQNM-AVGKQNLEVMIGNSNRS-QQDF
Opossum XP_007483702        ENHVI-IQAEFAQT-SN-----PEGEFMFDFDGDEIFHVDMD--KRETVWRLTDFSN-----FA-SFE------AQGALANL-AVDKANLEIMMKRSNNT-PDTN
Mouse H2-Ag7 a1-domain      ADHVGFYGTTVYQS-PG-----DIGQYTHEFDGDELFYVDLD--KKKTVWRLPEFGQ-----LI-LFE------PQGGLQNI-AAEKHNLGILTKRSNFT-PATN
Human HLA-DR1 a1-domain     EEHVI-IQAEFYLN-PD-----QSGEFMFDFDGDEIFHVDMA--KKETVWRLEEFGR-----FA-SFE------AQGALANI-AVDKANLEIMTKRSNYT-PITN
Human HLA-DQ1 a1-domain     ADHVASCGVNLYQF-YG-----PSGQYTHEFDGDEQFYVDLE--RKETAWRWPEFSK-----FG-GFD------PQGALRNM-AVAKHNLNIMIKRYNST-AATN
MHC-I alpha 1 domain
                                                                                     Q              S
                               tM R Y                                                N              N      rF  s
                               sL KYF T             F  VG V         S        P   w           Y       ET             D      yYnQt
Nurse shark UAA01           GSHSL-RYFFTWST-AG--SGIPEFVAVGYVDDQQFVQYDSD--RKEMIPRQRWVKESE---GPEYWER----ETQTLRGWE-PWGKANIDILSKRTNQT-G
Banded houndshark UAA*101   GSHSL-RYFHTSMT-PI--SGLPEFVIVGYVDELEFVHYDSD--MKKTIPRQRWMAESE---GPDYWER----NTQKARGWE-QWGKVNIPILSERTNQS-G
Banded houndshark UAA*201   GSHSL-RYFYTSMT-PI--SGLPEFVTIGYVDELEFVHYDSD--MKKTIPRQRWIAESV---GPDYWER----QTQIARGWE-QTGKVDIQTLMTRTNQS-G
Spiny dogfish UAA*01        GTHSL-RYFYNSMT-PI--PGVPEFVAVGYVDDALFVHYDSD--RKQMIPRQRWIEESE---DKQYWER----ETQKQLGWE-QIGKVDIQTLITRTNLT-G
Clearnose skate KC335152    GSHSL-RYFYTAVT-PG--SGVPEFTAVGYVDEEQIVYYDSD--RGKLEPRQRWMEESQ---DADYWER----ETRKLRDWE-QGFQINIQTLEQRTNQS-G
Clearnose skate KC335153    GSHSL-RYFYTGVN-PG--SGVPEFTAVGYVDEEQIVHYDSD--RGK-RSRQRWMEESQ---GADYWER----EHTDIRGAA-PVFKANIQTLEQRTNQS-G
Guitarfish AGJ01148         GSHSL-RYFYTGVT-PE--SGVPEFMTAGFVDEEQITYYSSV--VGRDKPRQQWMAESQ---GPDYWEQ----QTQILQGHE-QTFKSNIQIAMKRTNQT-G
Elephant shark JX207562     GSHSL-RYFYTAVH-GA--PGVPEFSIVGYVDDQQFVRYQSNSESRRMEPRQRWIQERE---DAGYWDG----QTRTAQGWE-QTYKVNVKTVMGRYNQT-G
Elephant shark JW872927     DSHSY-RYFYTGVQ-GA--PGVPEFTAVGYVDDLQFVRYQSNSESRRMEPRQRWIQERE---DAGYWDT----QTQILRSTE-QVYKVDMKTLMGRYNQT-G
Bichir SRAs                 DTQSL-KYFYTGSS-GV--TEFPEFVVVGLVNDEQFVYFDKN--INRMIPRQEWMAKSE---GPSYWDG----QTQGLIGSA-QNFKANLGILKERFNET-G
Reedfish XP_028647472       ATHSL-KYFYTGTS-GA--SEFPEFISLGMVDDKQTDYYDSN--IKKVIPRQEWMVKSV---DSDYWDR----ETQISVGDE-QNFKAGTATLNKRFNQT-G
Paddlefish UBA*01           GSHSL-RYFYTGTS-GV--TEFPEFVIVGMVDDVQISHCDSK--SKQTVPKQQWMKDNV---EPGYWER----NTQICLGNQ-QIFKVAVIDLPKRFNQT-E
Grass carp UAA106           GTHSL-KYVYTGVS-RG--IDFPEFTAVGMVDDGQFMYFDSN--SMKAVPKTEWIRQNE---GADYWDR----QTQVLIGAH-QVFKDSSQIVMERFNQS-K
Trout UBA*0103              VTHSL-KYFYTASS-EV--PNFPEFVVVGMVDGVQMVHYDSN--SQRMVPRQDWMNKAA-ETLPQYWES----QTGILKGTQ-QTYKASIDIVKQRFNQS-G
Medaka UAA*0101             VSHSL-KYFYTASS-QV--PNFPEFVAVGLVDDAQIDYYDSD--IRMDIPKQDWMKEAM---DEQYWER----ETGNKLGSQ-QTFKANIEIAKQRFNQT-G
Coelacanth XP_014346879     EPHTL-RYLYLGTM-NV--KDFPEFLTVGLVDNVRIRYFDSV--TGHCLSSQKWMEAFVEKEDPEYWES----MNRRLKSRH-QNFKENIQILRRRYNQT-N
W.A. lungfish SRAs          RSHSL-RYFYTGVS-GI--SEFPEFVLLGYVDDQTIGKFEST--DQVYKPQVQWMEENE---DQGYWTR----ETQNIQSWQ-QVFKANVKTLMDRTNQT-T
S.A. lungfish GEHZ01089118  GIHSL-RYFYTGVS-GT--SEFPEFVAMGYVDDTAISYYDSV--IKRYVPRAQWMKDNE---GQDYWDR----ETQIILGWQ-QAFKANVRTLMDRTNQT-S
African clawed frog UAAg    GSHSL-RYYYTAVSDRA--FGLPEFSIVGYVDDTQSFRYNSD--NQKAEPATQWMKQKE---GPEYWEQ----QTQIAKGSE-PVHKHDVKTAMDRFNQT-S
Axolotl Amme3               GSHSF-RGFYSSLSEEV--PGVPRFSAVGFVDDVPIEGYSSD--TRRWEPRAPWMRKIE---DPQYWER----ETERARGTE-PVERANVGIVMERLNQT-R
Anole lizard XP_016847220  ASSHSL-RYFYTGVTESI--PGLPHFIAVGYVDDQLFIQYDAH--TREAVPRVPWLDKAG-EADPQYWVR----NTQGLQNAE-VGFSVDLATLRERYNQT-G
Chicken BF2*0401            ELHTL-RYIRTAMTDPG--PGQPWFVTVGYVDGELFVHYNST--ARRYVPRTEWIAANT---DQQYWDG----QTQIGQLNE-QINRENLGIRQRRYNQT-G
Chicken BF2*2101            ELHTL-RYIRTAMTDPG--PGLPWFVDVGYVDGELFMHYNST--ARRAVPRTEWIAANT---DQQYWDR----ETQIVQGSE-QINRENLDILRRRYNQT-G
Echidna Taac2-2             RSHSL-RYFYTGVTRPG--LGVSEFTAVGYVDDVPFDRFDSD--RGRAEPRTSWIRNNE---GPEYWDR----NTQIYKGWA-QIFRGNLQTALVYYNQSKD
Platypus XP_028909408       RSHFL-RYFYTAVSRPG--PGVPAFTAVGYLDDQQFVRFDSN--RQKVEGLTTWIQGGQ---GPDYWEQ----QNQELRGTQ-QIFLQNLQVALSYYNQSEG
Platypus XP_028909721       RSHSL-RYFTTGVSRPG--PGVSEFTSVGYVDDVQFVRFDGDGRRGRAEPRTPWIRDNE---GREYWDQ----ETEIFRSGA-QNFHVGLQNIMGYYNQSEE
Opossum UC                  GSHSM-RYFHTAMSRPE--LGDSQFITVGYVDDQQFVRFDSSSESQRMEPRAAWMDKME-EEEPNYWEE----QTQIYRKNA-QIYRGNLETLRGYYNQSHG
Tasmanian devil I*11        GSHSL-RYFGTTVSRPG--LGEPRFFSVGYVDDQQFVGFDSDSASQRVEPRAPWIEKME-NVDRDYWER----NTQNSKRNA-QISREDLQTLHGYYNQSES
Mouse H2-Db                 GPHSM-RYFETAVSRPG--LEEPRYISVGYVDNKEFVRFDSDAENPRYEPRAPWMEQ-E---GPEYWER----ETQKAKGQE-QWFRVSLRNLLGYYNQSAG
Human HLA-C*0702            CSHSM-RYFDTAVSRPG--RGEPRFISVGYVDDTQFVRFDSDAASPRGEPRAPWVEQ-E---GPEYWDR----ETQKYKRQA-QADRVSLRNLRGYYNQSED
Human HLA-B*2705            GSHSM-RYFHTSVSRPG--RGEPRFITVGYVDDTLFVRFDSDAASPREEPRAPWIEQ-E---GPEYWDR----ETQICKAKA-QTDREDLRTLLRYYNQSEA
Human HLA-A2                GSHSM-RYFFTSVSRPG--RGEPRFIAVGYVDDTQFVRFDSDAASQRMEPRAPWIEQ-E---GPEYWDG----ETRKVKAHS-QTHRVDLGTLRGYYNQSEA
                            .1        .10         .20       .30       .40       .50           .60           .70       .80        .90
                              sss-sssssss          ssssssss  sssssss       ss   hhh        hhhhhh----hhhhhhhhh-hhhhhhhhhhhhhh
II-b1/I-a2 lineage ancestral residues
                                                                           Y                                 Q
                                                       r  Y                W      g                          E                     R
                                  g                    q  F                F      a                          D                   L K
MHC-II beta 1 domain
                                         Y   T                             Y                                       N
                                         F N S                             F                                       H
Nurse shark clone 11        GAHSE-ISLHRCVF-NST--GDWVFLKQQVYDQELIAYYDYN--QRKFIAVKAWMKS-----NVDRWNR---EGAEEQYESGKAYCEHNIPIVY---ESA-LARQ
Ray EW694773                GAHSL-TKIGGCAF-NSSAPGEWTYFSKGIYDGEVIWYFDFD--QRKFVAVKGWMKG-----NMDRWNK---EAAESTYQRGLSMCENNIPLYG---REV-LPRR
Elephant shark  JW875734    EAESY-IFRSGCEFNNGT---SWTYVEEQIYNKQVIAYYDFN--QRKYIANKQWTKP-----SVDTWN----QQAEETYQEGIRVCENNAQIFD---RIV-LTRL
Elephant shark JW875394     EADSN-IFRQGCEFNNGT---SWTFVYEEIYNKQLIAYYDFN--QRKYIAVKQWTKP-----SVDSWN----QHAEETYQEGIRICENNVDVYN---RVE-LTRL
Bichir SRAs                 DGYMH-HVQRECRY-NSHSLEGMEFIDRYIFNHEEYIRYNST--LNKFIGYTEHGVK-----NAEQANS-DPSELAGQKSNVDATCKPNAKLYY---DTI-LDKS
Reedfish XP_028681436       EGYTY-HVQRECRF-NSHSLEGTEYIERRIFNKLEITRYNST--LGKFIGYTEIGVY-----NADRFNN-DSGVMAGMKSNVDAVCKRNAQIEY---DHI-LDKS
Paddlefish DAB*01           EGYLM-QTLVDCEY-SDSSMTDMVYSWNYVFNKQEVVHYDSK--IKKYVGNTACGVK-----NAEVWNK-DTAQLAGLLGDVDRYCKHNAELYM---LFT-TDRK
Trout DAB*1602              DGYAS-DVVTRCLY-SSIDMHGAEFIQSYTFNKVEHLRFNST--VGEFVGYTELGLK-----NAKRLNR-G-QEVVQMRGELERLCEPNADVHY---RAI-LDKT
Zebrafish DAB1*01           DGYYQ-YTMLECIY-STSDYSDMVLLESGSFNKVVDVQYNST--VGKYVGYTEQGVI-----FARNFNK-NQAYLQQRKAEVESFCRHNAQISD---SAV-RDKA
Stickleback DAB*01          DGFMM-FVTDECVF-NSTELKDIEFIRSSYFNKKEDTRFSSS--VGKFVGFTEQGVK-----IAANWNK-DASFLSAMKAQKEVYCLNHVPVYY---TAA-LTKS
Coelacanth XP_006014459     ISNVE-QYQWECHYTNGT--QDIDFIHRVIYNGQEESYFDSR--IGKFTGVTEWGKK-----DADYWNK-DKENLAQWRIQEDRWCRNNYNWMQ---GWA-VGKQ
S.A. Lungfish GEHZ01038869  DGFVL-QLSKVCFFSGQQETKDMEFTYVFTFNKQIIMFYNQT--LGIFLEAGFYPIDD----LVDYMNR-QTDLLKQLNTAVDTVCRSNMIKYW---DRT-AGRK
Giant salamander AGY56015   ADFVT-QAKAECHFLNGS--ERVRFLLRYFYNQEELAYFDSD--VGRFVPKTEFGKP-----DADYWNS-DPAILEDARAAVERYCKHNYDVRK---QDA-RSWK
African clawed frog DAB     EDYVY-QYKAQCYFRNGT--DNVRLLWRHYYNLEETDYFDSD--VGLFIAKTELGKP-----SADYWNS-QKETLEQKRAAVDTVCRHNYPFDK---PFT-IDRK
Chicken B-LBII              AFFFC-GAISECHYLNGT--ERVRYLQRYIYNRQQFTHFDSD--VGKFVADSPLGEP-----QAEYWNS-NAELLENRMNEVDRFCRHNYGGVE---SFT-VQRS
Mouse H2-Ag7 b1 domain      RHFVH-QFKGECYFTNGT--QRIRLVTRYIYNREEYLRFDSD--VGEYRAVTELGRH-----SAEYYNK-Q--YLERTRAELDTACRHNYEETE---VPTSLRRL
Human HLA-DR1 b1 domain     PRFLW-QLKFECHFFNGT--ERVRLLERCIYNQEESVRFDSD--VGEYRAVTELGRP-----DAEYWNS-QKDLLEQRRAAVDTYCRHNYGVGE---SFT-VQRR
Human HLA-DQ1 b1-domain     EDFVY-QFKGLCYFTNGT--ERVRGVTRHIYNREEYVRFDSD--VGVYRAVTPQGRP-----VAEYWNS-QKEVLEGARASVDRVCRHNYEVAY---RGI-LQRR
MHC-I alpha 2 domain
                                                                 T                                                    R
                                    M                Y r G       YLS                g                                   QR
                                  q L G      D      GF q A DG DFIA                a       T  K              YL      W KKY   G
Nurse shark UAA01           GIHTY-QLMCGCEL-RDD-GSSNTGFVQHAWDSTDFISLDKD--KMVWVTPVTWGEI-----TKNKWDR-DMAFNQGTKGYLEGICIEWLQKYLKNGNVE-L-RP
Banded houndshark UAA*101   GIHVL-QRMSGCEL-RED-GS-TGGFFQFGWDGKNLISFDKE--QSVWVTPVQWGEI-----TKKKWDK-DQGLNQQWKGYLEGICIEWLKKYLRYGERQ-L-RP
Banded houndshark UAA*201   GIHVL-QRMYGCEL-RED-GS-TGGFFRDGWDAKDSIVFDKE--HLRWIAVGQWAVL-----TKEKWDK-DQGLNQQRKGYLEGICIEWLKKYLRYGERQ-L-RP
Spiny dogfish UAA*01        GIHTL-QVMYGCEL-RDD-GS-TAGFFQYGWDGKDLISFDKE--HLVWNTPVTWQVV-----TKNKWEQ-DRGLGQQRKGYLEQECIEWLKKYLTAGERE-L-KP
Clearnose skate KC335152    GIHTI-QRMFGCEL-RSD-GS-SAGFSQYGWDGEDLISFDKE--HMVYVTPVTWGLL-----TKHKWEQ-DTGIAHQNKAYLEGTCTEWLQKYVQAGQSQ-L-TP
Clearnose skate KC335153    GIHTV-QWMYGCAV-RSD-GS-TAGFDQYGWDGEDFISFDKE--HMVYVTPVTWGLL-----TKHKWDR-ETGGNHQRKGYLEGTCIEWLKKYVQAGQSQ-L-TP
Guitarfish AGJ01148         GIHTF-QRMYGCEI-LDD-GT-TTGFDQFGWDGSDFISFKKD--EMVWVTPVPWGQI-----TKAKWDR-DMAWNQGNKGYLEQICIEWLKKYVKAGESN-L-KP
Elephant shark JX207562     GIHTV-QQMYGCEL-KGD-GS-VGGFIQYGYDGNDFIAFDKD--RKVWTAPTAAAVV-----TKNKWEN-TPGLSEQEKDYLEQICIEWLRKYVEYGHES-L-RP
Elephant shark JW872927     GIHTV-QLMYGCEL-REG-RS-VGGFYQDAYDGNDFIAFDKD--QMVWVAPTAAAVI-----TKNKWEN-TPGVSEQWKDYLEQICIEWLRKYVEYGHES-L-RP
Bichir SRAs                 GIHTL-QFMYGCIK-YDD-DT-TGMFRQFAYDGNDFISFDVN--TLSFTATSPQAAV-----TKKKWEE-NEGFVQFLKNYYQKECFDWLNKYVKYGRST-LERK
Reedfish XP_028647472       GIHTW-QMMYGCEL-DDD-GT-TRGFHQFGYDGDDYVSFDKQ--TLTWTAASPRGET-----TKIRWDP-DTAWNLQTKGYLEGTCIEWLKKYVVYGAAT-LERK
Paddlefish UBA*01           GVHTV-QRMVGCEL-DDD-GT-KRGFEQHGYDGEDYIMFDKD--TLTWTAASQRGFT-----TKVKWDP-LTASNQQRKAYLEGTCIEWLKKYVQYGRET-LERR
Grass carp UAA106           GVHTW-QNMYGCEL-NDD-GT-TQGFYQYAYDGEDFVSLDKN--TLTWTAANPQAVI-----TKHKWEA-L-AVAEQNKGYLENTCIEWLKKYVAYGKDT-LERK
Trout UBA*0103              GVHTF-QNMYGCEW-DDD-TGATEGFFQYGYDGEDFLALDLK--TKKWIAPTPQAVI-----TKHKWDS-NTANEERRKHYLTQECIEWLKKYLDYGKST-LMRT
Medaka UAA*0101             GVHVY-QWMYGCEW-DDE-TGEINSFHQYGYDGEDFIALDLK--TESWIAAKQQAFI-----TKQKWDN-DNAKKAQDKNYFTQICPEWLKKYVNYGSSS-LMRK
Coelacanth XP_014346879     GAHSL-QWTHGCTL-GET-GESVSGFMRFGYDGEDFISFNME--GLNWVAAKQEAKI-----TKDKWDK-DQALNHQTKGYLQDECIHWLKKYLEYGNGD-LGRK
W.A. lungfish SRAs          GVHTN-QVMYGCSL-NDD-GT-TTGEEQHAFDGKDFISFDKD--SLTWNAAALYGVI-----TVNRWNS-DANFNMQTKGYLEQTCIEWLKKYTQYGQSS-LEQK
S.A. lungfish GEHZ01089118  GIHSF-QEMYGCYL-HDD-GT-KSGFFQYGYDGQDYITFDKE--TQTWTAVAPSAVF-----SKNKLDP-DTATNMYLNSYLEQECIESLQRYIKYGQTS-LGKK
African clawed frog UAAg    GTHSL-QVMYGCEL-RED-NS-IRSYHQYGYDGREFIALDTE--RWVYVPSVREAQL-----TEQKWNSPEVNAPERNKNYLQNLCIEGLKRYLSYGRAE-LERR
Axolotl Amme3               GLHTV-QQMYGCEL-RGD-GS-IGGFMQYAYDGGDFLSFDKD--RMRYIATAPPAQV-----SVDRLNS-EKIQAQRQKAYLEGECIEWLQKYLQYGEET-LLRR
Anole lizard XP_016847220   GFHTW-QWMYGCEL-RKD-GS-KGGYFQSAYDGRDYISFDKE--TLTYTAADVPAQN-----TKRKWEA-EPAIAQRNKAYLEDICIEWLQRYLDYGKET-LLRT
Chicken BF2*0401            GSHTV-QWMFGCDI-LED-GT-IRGYRQSAYDGRDFIALDKD--MKTFTAAVPEAVP-----TKRKWEE-E-SEPERWKNYLEETCVEWLRRYVEYGKAE-LGRR
Chicken BF2*2101            GSHTV-QWMSGCDI-LED-GT-IRGYHQAAYDGRDFVAFDKG--TMTLTAAVPEAVP-----TKRKWEE-G-GYAEGLKQYLEETCVEWLRRYVEYGKAE-LGRR
Echidna Taac2-2             GIHSV-QLMYGCQL-RED-NTTGSGFFQFGYDGQDYITLDKA--TLSWTAADGGAQN-----TKRKWEA-DRTIAEQDKAYLEGECIDGLRRYLRYGGPS-LTRA
Platypus XP_028909408       GYHSY-QEMYGCEL-RTD-GSTGKAYDRYGYDGQDYITLDLD--TLTWTAANPEAQY-----TKRKWEA-NKKKLELEKAYLQGQCVYWISEYLKLGGDS-LNRT
Platypus XP_028909721       RSHTV-QGLYGCQL-RED-NTPGEGFMQFGYDGRDYIALDKA--TLSWTVADAGALN-----TKSKWEA-DGTIAERYKAYLEDECIEWLRRYMGFGGPC-LTRA
Opossum UC                  GLHTI-QRMIGCEV-SAE-GSLRKGFYQFAYDGHDYIALHTE--TLSWTAADTGAEN-----TKRKWEP-EKSIAESYKAYLEKECTHWLKKYLEMGKDV-LLRT
Tasmanian devil I*11        GVHTF-QRLVGCEI-SPD-LSFKRGFDQYAYDGQDYLALDTE--TLTWTAAVNEAVN-----SKHKLEA-ERSNAERDKAYLEETCVLWLKMYLEMGKES-LQRA
Mouse H2-Db                 GSHTL-QQMSGCDL-GSD-WRLLRGYLQFAYEGRDYIALNED--LKTWTAADMAAQI-----TRRKWEQ-S-GAAEHYKAYLEGECVEWLHRYLKNGNAT-LLRT
Human HLA-C*0702            GSHTL-QRMSGCDL-GPD-GRLLRGYDQSAYDGKDYIALNED--LRSWTAADTAAQI-----TQRKLEA-A-RAAEQLRAYLEGTCVEWLRRYLENGKET-LQRA
Human HLA-B*2705            GSHTL-QNMYGCDV-GPD-GRLLRGYHQNAYDGKDYIALNED--LSSWTAADTAAQI-----TQRKWEA-A-RVAEQLRAYLEGECVEWLRRYLENGKET-LQRA
Human HLA-A2                GSHTV-QRMYGCDV-GSD-WRFLRGYHQYAYDGKDYIALKED--LRSWTAADMAAQT-----TKHKWEA-A-HVAEQLRAYLEGTCVEWLRRYLENGKET-LQRT
res. number HLA-A2 a1       .1        .10         .20       .30       .40       .50           .60           .70       .80        .90
res. number HLA-A2 (a2)*              .100        .110      .120        .130    .140              .150       .160     .170       .180
                               ss-ssssssss       ssssssssss  ssssss       sss  hhhhh-----hhhhhhh    hhhhhhhhhh hhhhhhhhhhhh hhh-h
```

## Supplementary file 1A(b)

Alignment of deduced MHC-I and MHC-II IgSF domain amino acid sequences

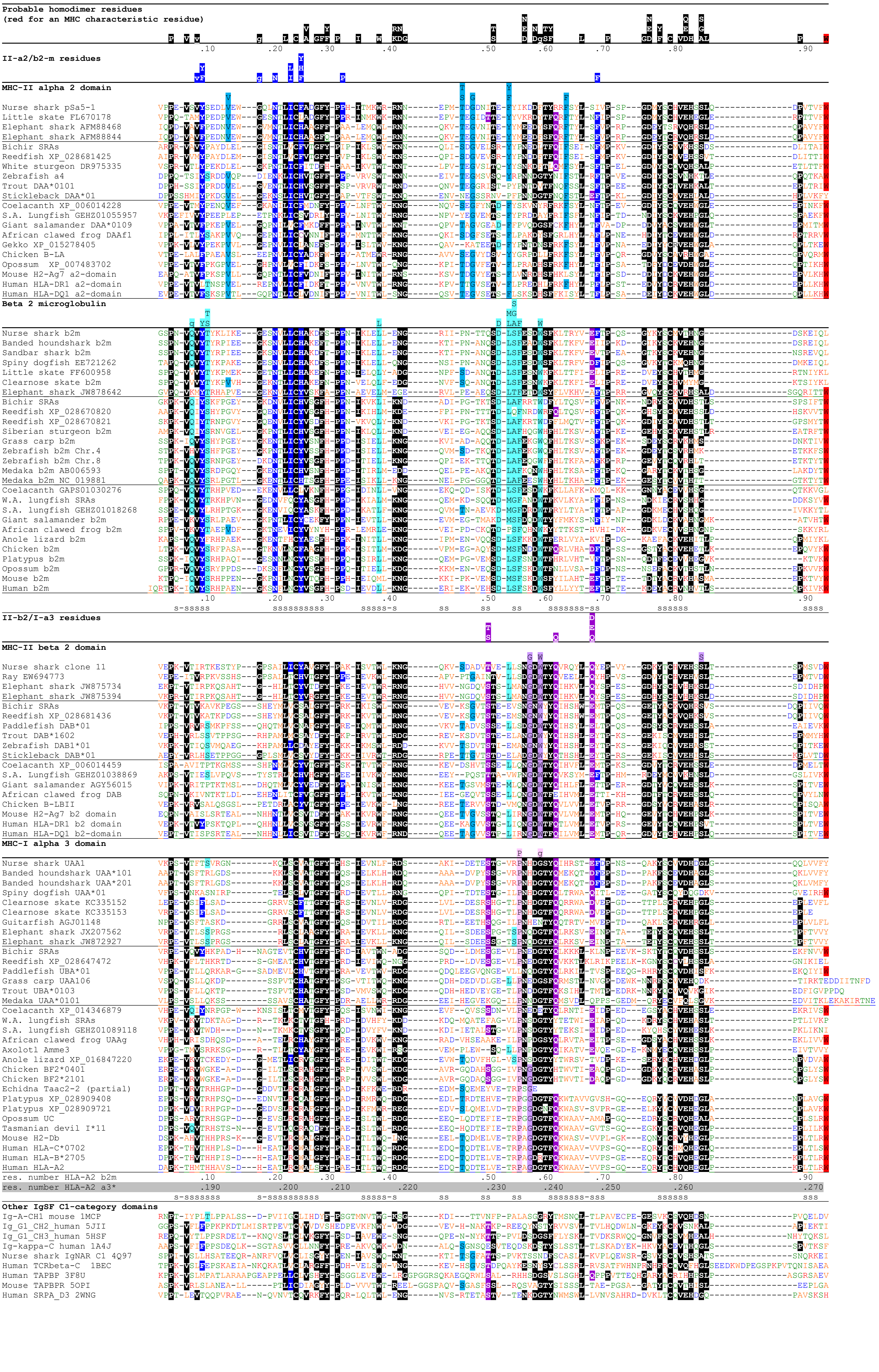

# Supplementary file 1B(a)

Superposition of the peptide-binding domains of structures whose sequences are aligned in main text Fig. 3A

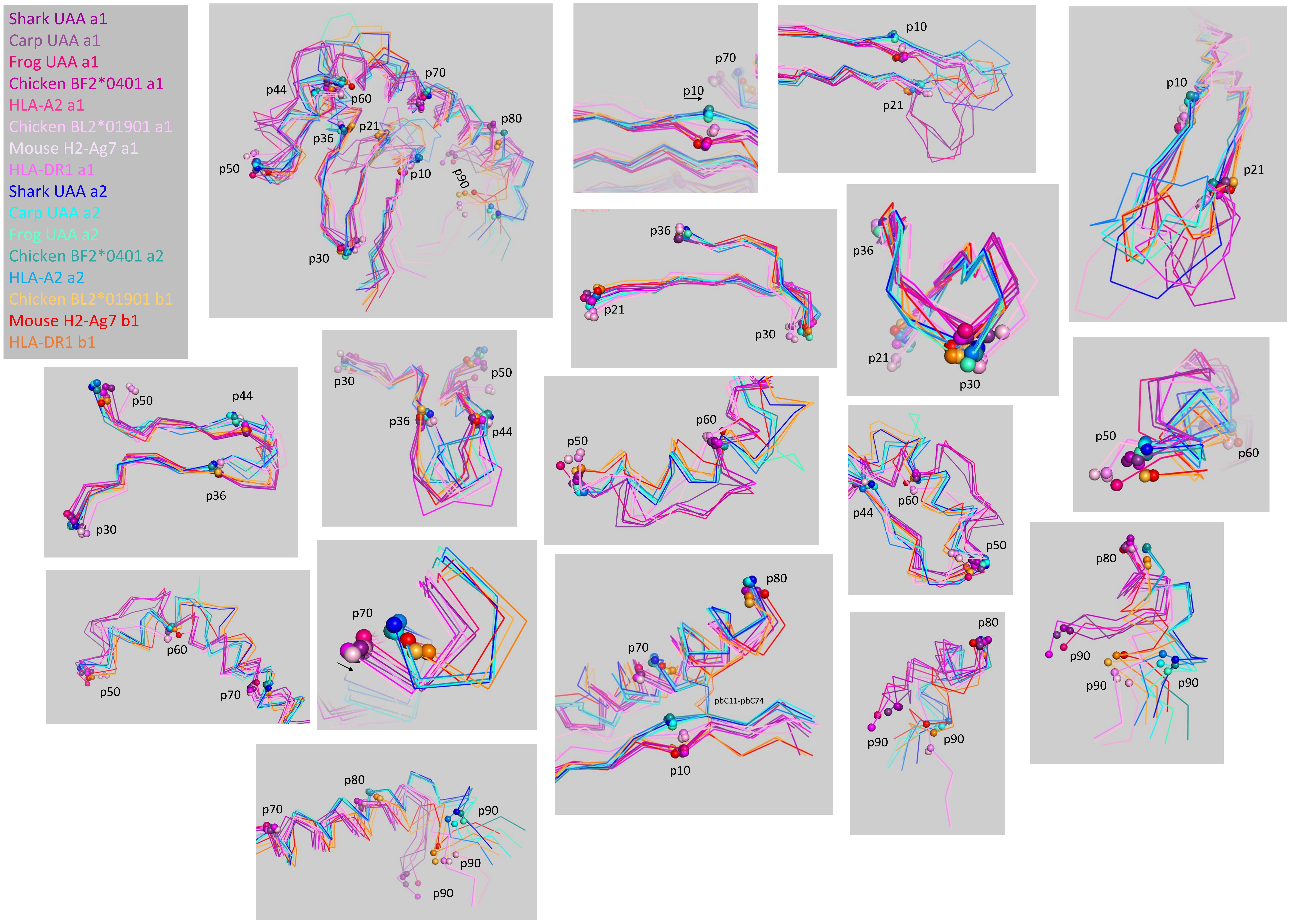

# Supplementary file 1B(b)

Superposition of the IgSF domains of structures whose sequences are aligned in main text Fig. 3B

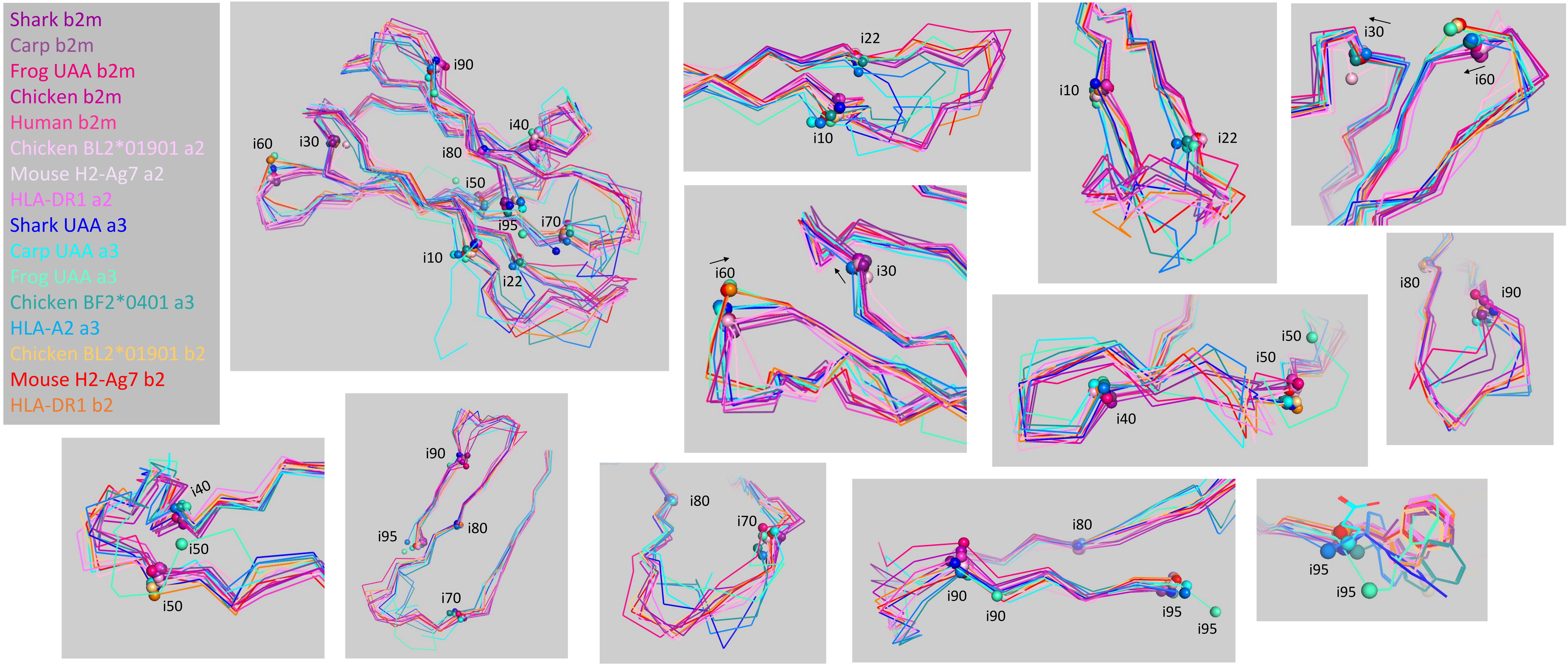

## Supplementary file 1C

# Amino acid identity percentages per identical domain between representative MHC-I and MHC-II sequences

Colors help to see similarity levels at a glance: yellow, <30% aa identity; light orange, 30-40% aa identity; orange, 40-50% aa identity; dark orange, >50% aa identity.

| Domain | Class | Sequence | MHC-I: Nurse shark UAA01 | Grass carp UAA106 | African clawed frog UAAg | Chicken BF2*0401 | Human HLA-A2 | MHC-II: Nurse shark pSa5-1 | Nurse shark clone 11 | Trout DAA*0101 | Trout DAB*1602 | African clawed frog DAAf1 | African clawed frog DAB | Chicken BL2*01901 | Human HLA-DR1 |
|---|---|---|---|---|---|---|---|---|---|---|---|---|---|---|---|
| **pa domain** | **MHC-I** | Nurse shark UAA01 | | | | | | | | | | | | | |
| **(I-$\alpha$1 or II-$\alpha$1)** | | Grass carp UAA106 | 43% | | | | | | | | | | | | |
| | | African clawed frog UAAg | 48% | 47% | | | | | | | | | | | |
| | | Chicken BF2*0401 | 40% | 34% | 36% | | | | | | | | | | |
| | | Human HLA-A2 | 45% | 38% | 45% | 37% | | | | | | | | | |
| | **MHC-II** | Nurse shark pSa5-1 | | | | | | | | | | | | | |
| | | Trout DAA*0101 | | | | | | 24% | | | | | | | |
| | | African clawed frog DAAf1 | | | | | | 19% | | 17% | | | | | |
| | | Chicken BL2*01901 | | | | | | 25% | | 17% | | 30% | | | |
| | | Human HLA-DR1 | | | | | | 31% | | 26% | | 39% | | 52% | |
| **pb domain** | **MHC-I** | Nurse shark UAA01 | | | | | | | | | | | | | |
| **(I-$\alpha$2 or II-$\beta$1)** | | Grass carp UAA106 | 48% | | | | | | | | | | | | |
| | | African clawed frog UAAg | 38% | 42% | | | | | | | | | | | |
| | | Chicken BF2*0401 | 42% | 55% | 51% | | | | | | | | | | |
| | | Human HLA-A2 | 38% | 51% | 38% | 55% | | | | | | | | | |
| | **MHC-II** | Nurse shark clone 11 | | | | | | | | | | | | | |
| | | Trout DAB*1602 | | | | | | | 18% | | | | | | |
| | | African clawed frog DAB | | | | | | | 26% | | 22% | | | | |
| | | Chicken BL2*01901 | | | | | | | 19% | | 20% | | 44% | | |
| | | Human HLA-DR1 | | | | | | | 21% | | 23% | | 58% | 58% | |
| **ia domain** | **MHC-I** | Nurse shark UAA01 | | | | | | | | | | | | | |
| **($\beta_2$-m or II-$\alpha$2)** | | Grass carp UAA106 | 43% | | | | | | | | | | | | |
| | | African clawed frog UAAg | 33% | 39% | | | | | | | | | | | |
| | | Chicken BF2*0401 | 35% | 37% | 26% | | | | | | | | | | |
| | | Human HLA-A2 3 | 40% | 49% | 30% | 48% | | | | | | | | | |
| | **MHC-II** | Nurse shark pSa5-1 | | | | | | | | | | | | | |
| | | Trout DAA*0101 | | | | | | 43% | | | | | | | |
| | | African clawed frog DAAf1 | | | | | | 34% | | 36% | | | | | |
| | | Chicken BL2*01901 | | | | | | 41% | | 35% | | 36% | | | |
| | | Human HLA-DR1 | | | | | | 44% | | 39% | | 49% | | 51% | |
| **ib domain** | **MHC-I** | Nurse shark UAA01 | | | | | | | | | | | | | |
| **(I-$\alpha$3 or II-$\beta$2)** | | Grass carp UAA106 | 24% | | | | | | | | | | | | |
| | | African clawed frog UAAg | 31% | 31% | | | | | | | | | | | |
| | | Chicken BF2*0401 | 32% | 26% | 39% | | | | | | | | | | |
| | | Human HLA-A2 | 27% | 27% | 29% | 33% | | | | | | | | | |
| | **MHC-II** | Nurse shark clone 11 | | | | | | | | | | | | | |
| | | Trout DAB*1602 | | | | | | | 44% | | | | | | |
| | | African clawed frog DAB | | | | | | | 38% | | 39% | | | | |
| | | Chicken BL2*01901 | | | | | | | 43% | | 41% | | 44% | | |
| | | Human HLA-DR1 | | | | | | | 44% | | 44% | | 47% | 54% | |
