# Supplementary material for: Structural Comparison Between MHC Classes I and II; in Evolution, a Class-II-Like Molecule Probably Came First
Source: Front Immunol. 2021 Jun 14;12:621153. doi: 10.3389/fimmu.2021.621153 (PMC8236899; doi:10.3389/fimmu.2021.621153)
Supplement: Supplementary file 1 [file DataSheet_1.zip › Supplementary File 2.pdf]

Supplementary file 2

pMHC interdomain interactions analyzed by PDBePISA software

Table of Contents **Page**

Legend to Supplementary files 2A, 2B, 2C, and 2D 2

2A: Residues that are part of the interface between pMHC pab and ia domains 3

2B: Summary of predicted hydrogen bonds and salt bridges between pMHC pab and ia domains 5

2C: Residues that are part of the interface between pMHC ia and ib domains 7

2D: Summary of predicted hydrogen bonds and salt bridges between pMHC pab and ia domains 9

Legend to Supplementary files 2A, 2B, 2C, and 2D

The pMHC-I structures were analyzed on-line by PDBePISA software (https://www.ebi.ac.uk/msd-srv/prot_int/pistart.html), using the functions “Interfaces” and “Details”. The software provided a list of “Interfacing residues” together with their Accessible Surface Area (ASA) in Å², Buried Surface Area (BSA) in Å², Solvation energy effect $\Delta^{i}G$ in kcal/mol, and predictions of hydrogen bonds (H) and salt bridges (S); for shark pUAA domains these values are indicated in Supplementary files 2A and 2C, except that instead of the ASA value the BSA/ASA percentage value is given. For the pMHC-I structures carp UAA (PDB 5Y91), frog UAA (PDB 6A2B), chicken BF2*0401 (PDB 4E0R), and HLA-A2 (PDB 3PWN), and the pMHC-II structures chicken BL2*01901 (PDB 6KVM), mouse H2-Ag7 (PDB 1F3J), and HLA-DR1 (PDB 1AQD), in the same files only the “Interfacing residues” are shown together with information on hydrogen bond and salt bridge formation. Residues shown in Supplementary files 2A and 2C that are not part of the interdomain interface, unlike some of their counterparts in the other compared pMHC structures, are shown in gray-shaded small Italic font and are followed by a “-” symbol; “nd” in gray shading indicates not determined because that part of the structure was not resolved; non-shark pMHC residues that do form part of the interdomain interface are followed by a “+” symbol. Except for gray, the residue shading colors used in Supplementary files 2A and 2C match with those used in Supplementary file 3 and relate to conservation patterns.

The software also provided a more detailed summary of the predicted hydrogen bonds and salt bridges, which is shown in Supplementary files 2B and 2D; in these tables, interesting conserved pairs of interacting residues or atoms are indicated with colors.

(2A) Residues that are part of the interface between pMHC pab and ia domains. The numbers 8, 9, and 10 in the “pa9-pl.” column refer to the top residues (“9”) and lower ridge residues (“8” and “10”) of the a pa9 pleat and are named after their respective constituent residues pa8, pa9, and pa10. As explained in the main text, in pMHC-I structures the pab domain pa9 pleat forms a major part of the pab domain interaction with the ia domain (β_2-m), and a difference with pMHC-II structures is that only in pMHC-I structures the ia residues interact with the pleat top residues (the “9” positions).

(2B) Summary of predicted hydrogen bonds and salt bridges between pMHC pab and ia domains.

(2C) Residues that are part of the interface between pMHC ia and ib domains. Note that also β_2-m residues C-terminal of the IgSF-exon-encoded stretch (ia95a-to-d) and matching pMHC-II residues are included (below the dashed line).

(2D) Summary of predicted hydrogen bonds and salt bridges between pMHC ia and ib domains.

Supplementary file 2A

Residues that are part of the interface between pMHC pab and ia domains

Residues in the pab domain:

		Shark UAA				*Other pMHC structures:*						
	pa9-		BSA			pMHC-I				pMHC-II		
pab	pl.		% / Å²	H/S	Δ^iG	Carp	Frog	Chick	HLA-A2	Chick	H2-Ag7	HLA-DR1
pa5a		--				--	--	--	--	--	F+	--
pa6		r-				K+	R+	r-	R+	L+	y-	I+
pa8	8	F	79% / 46		0.72	V+	Y+	I+	F	A+	T+	A+
pa9	9	F	21% / 2		-0.02	Y+	Y+	R+	F+	e-	t-	e-
pa10	10	T	97% / 40		0.11	T+	T+(H)	T+	T+	F+	V+	F+
pa12		S	47% / 11		-0.08	V+	V+	M+	V+	Q+(H)	Q+(H)	L+
pa14		--				--	d-	D+(HS)	r-	--	--	--
pa15		a-				R+	r-	P+	p-	S+	p-	p-
pa16		g-				G+	A+	G+	g-	e-	g-	D+
pa17		S	16% / 19		-0.15	I+(H)	F+	P+	R+	p-	--	--
pa18		G	5% / 4		0.07	D+(HS)	G+	g-	g-	d-	--	--
pa19		I	75% / 79		1.27	F+(H)	L+	Q+(H)	e-	k-	--	--
pa21		e-				e-	e-	w-	R+	W+	I+	S+
pa23	10	V	82% / 33		0.49	T+	s-	V+	I+	Q+(H)	Q+(H)	E+(H)
pa25	8	V	97% / 32		0.51	V+	V+	V+	V+	g-	T+	M+
pa27		Y	51% / 29		-0.10	M+	Y+(H)	Y+	Y+(H)	H+	E+	D+
pa30		d-				d-	d-	g-	e-	A+	G+	G+(H)
pa31		q-				g-	t-	e-	t-	D+	D+	D+
pa32		Q	32% / 16	H	-0.25	Q+	Q+	L+	Q+(H)	E+(H)	E+(H)	E+(H)
pa35	8	Q	55% / 9		0.03	Y+	R+(HS)	H+	R+(HS)	H+	Y+	H+
pa37		--				--	--	--	--	E+	--	--
pa46		--				--	--	--	--	V+	--	--
pa47		p-				p-	p-	p-	p-	w-	W+	w-
pa48		R	14% / 18	HS	-0.43	K+(S)	a-	R+(HS)	R+(HS)	R+	R+	R+(H)
pa87		q-				Q+	q-	q-	Q+	r-	f-	y-
pa90a		--				--	--	--	--	Q+	A+	I+
pa90b		--				--	--	--	--	D+	T+(H)	T+(H)
pa90c		--				--	--	--	--	F+	N+(H)	N+
pb1		g-				g-	g-	g-	G+	a-	r-	p-
pb2		I	44% / 69		0.94	V+	T+	S+	S+	F+	H+	R+
pb3		H	3% / 0.5		0.01	H+	h-	H+	H+	f-	f-	f-
pb4		T	92% / 27		-0.13	T+	S+(H)	T+	T+	F+	V+	L+
pb6	10	Q	100% / 62	H	-0.36	Q+(H)	Q+(H)	Q+(H)	Q+(H)	g-	Q+(H)	Q+
pb7	9	L	69% / 2		-0.00	N+	V+	W+	R+	a-	f-	l-
pb8	8	M	81% / 57		1.72	M+	M+	M+	M+	I+	K+(H)	K+(H)
pb10		g-				g-	g-	g-	g-	e-	e-	E+
pb12		e-				e-	e-	d-	d-	h-	y-	H+
pb23		f-				F+	Y+	y-	y-	l-	V	l-
pb25	8	Q	34% / 26		-0.06	Q+	Q+	Q+	Q+	R+	R+	R+
pb26	9	H	39% / 4		-0.00	Y+	Y+	S+	Y+	y-	y-	c-
pb27	10	A	99% / 18		0.29	A+	G+	A+	A+	I+	I+	I+
pb29		D	48% / 22	H	0.07	D+(S)	D+	D+	D+	N+	N+	N+
pb30		S	83% / 70	H	0.15	G+	G+	G+	G+	R+(H)	R+(HS)	Q+(H)
pb31		T	31% / 24		0.39	E+	R+	R+	K+	Q+	e-	e-
pb32	10	D	22% / 23	H	-0.21	D+(H)	E+	D+(H)	D+(H)	Q+	E+	E+

Residues in the ia domain:

	Shark UAA				*Other pMHC structures:*						
		BSA			pMHC-I				pMHC-II		
ia		% / Å²	H/S	$\Delta^{i}G$	Carp	Frog	Chick	HLA-A2	Chick	H2-Ag7	HLA-DR1
ia3	*s-*				*s-*	*s-*	*l-*	R+	V+	E+(HS)	V+(H)
ia4	*s-*				*s-*	*p-*	*t-*	*t-*	T+	*a-*	P+
ia29	*d-*				*g-*	*n-*	*g-*	*g-*	*k-*	N+	*k-*
ia31	S	66% / 14		0.19	H+(S)	H+(H)	H+	H+(H)	W+	F+	T+
ia32	P	70% / 34		0.54	P+	P+	P+	P+	P+	P+	P+
ia33	P	96% / 52		0.48	P+	P+	P+	S+	P+(H)	P+(H)	P+
ia34	N	65% / 52		0.29	D+(H)	R+	K+(HS)	D+	V+	V+	V+
ia35	I	53% / 14		-0.14	I+(H)	L+	I+	*i-*	*a-*	*i-*	*v-*
ia36	*k-*				*s-*	E+	*s-*	*e-*	*t-*	*n-*	*n-*
ia50	*t-*				*q-*	*k-*	*q-*	*e-*	Y+	Y+	*s-*
ia51	Q	17% / 11		-0.13	Q	*q-*	E+	H+	*d-*	*e-*	E+
ia52	S	1% / 0.5		0.01	T+	T+	S+	S+	S+	T+	T+
ia53	D	81% / 105	HS	-0.26	D+(S)	D+(HS)	D+(HS)	D+(HS)	V+	S+(H)	V+(H)
ia54	L	93% / 87		0.65	L+	P+	M+	L+	Y+(H)	F+(H)	F+(H)
ia55	S	91% / 42		0.09	A+	S+	S+	S+	Y+(H)	L+	L+
ia56	F	97% / 103		1.21	F+	F+	F+	F+	G+	V+	P+
ia57	E	4% / 4		0.04	E+	Q+	*n-*	S+	R+	N+(H)	R+(H)
ia58	S	27% / 31		0.18	K+	H+	D+	K+	*p-*	R+	E+
ia59	D	4% / 4		-0.04	G+	N+	D+	D+	D+(H)	D+(HS)	D+(H)
ia60	W	95% / 173	H	2.04	W+(H)	W+(H)	W+(H)	W+(H)	L+	H+(H)	H+
ia61	S	5% / 0.7		0.01	*q-*	K+	*t-*	*s-*	L+	S+	L+
ia62	F	99% / 37		0.60	F+	Y+(H)	F+	F+	F+	F+	F+
ia63	K	15% / 10		-0.35	H+	Y+(H)	*q-*	Y+(H)	R+	H+	R+(H)
ia64	L	84% / 7		0.11	L+	*t-*	R+(H)	*l-*	*k-*	*k-*	*k-*
ia67	*y-*				*s-*	*s-*	*h-*	Y+	Y+	Y+	Y+(H)
ia83	*t-*				R+(HS)	*s-*	*e-*	*n-*	*r-*	*e-*	*e-*
ia84	*h-*				*h-*	*h-*	*h-*	*h-*	H+	H+	H+
ia85	N+	7% / 6		-0.06	M+	*n-*	E+	*v-*	W+	W+(H)	W+
ia86	*g-*				*s-*	*g-*	*t-*	*t-*	G+	G+	G+
ia88	--				--	*p-*	*k-*	*s-*	E+	*e-*	*d-*

Supplementary file 2B

Summary of predicted hydrogen bonds and salt bridges between pMHC pab and ia domains

pMHC-I

```
In Shark UAA:
Hydrogen bonds
paQ32  [NE2]   2.76  A   iaD53  [OD2]
paR48  [NE ]   2.88  A   iaD53  [OD2]
paR48  [NH1]   3.03  A   iaD53  [OD1]
pbQ6   [NE2]   3.02  A   iaW60  [O   ]
pbS30  [OG ]   3.67  A   iaW60  [O   ]
pbD32  [OD1]   2.83  A   iaW60  [NE1]
Salt bridges
paR48  [NE ]   3.81  A   iaD53  [OD1]
paR48  [NE ]   2.88  A   iaD53  [OD2]
paR48  [NH1]   3.03  A   iaD53  [OD1]
paR48  [NH1]   3.59  A   iaD53  [OD2]
```

```
In Carp UAA:
Hydrogen bonds
paI17  [N   ]   3.50  A   iaD34  [OD1]
paD18  [OD1]   2.96  A   iaI35  [N   ]
paD18  [OD1]   3.48  A   iaR83  [NH2]
paF19  [N   ]   3.85  A   iaD34  [OD2]
pbQ6   [NE2]   2.84  A   iaW60  [O   ]
pbD32  [OD1]   2.64  A   iaW60  [NE1]
Salt bridges
paD18  [OD1]   3.48  A   iaR83  [NH2]
paK48  [NZ ]   3.99  A   iaD53  [OD2]
pbD29  [OD1]   4.00  A   iaH31  [NE2]
```

```
In Frog UAA:
Hydrogen bonds
paT10  [OG1]   2.78  A   iaY62  [OH ]
paY27  [OH ]   2.65  A   iaY63  [OH ]
paR35  [NH1]   3.49  A   iaD53  [OD1]
pbS4   [OG ]   3.49  A   iaH31  [NE2]
pbQ6   [OE1]   3.39  A   iaH31  [NE2]
pbQ6   [NE2]   3.09  A   iaW60  [O   ]
Salt bridges
paR35  [NH1]   3.49  A   iaD53  [OD1]
```

```
In Chicken BF2*0401
Hydrogen bonds
paD14  [OD2]   2.85  A   iaK34  [NZ ]
paQ19  [OE1]   2.90  A   iaR64  [NH2]
paR48  [NH1]   3.05  A   iaD53  [OD1]
paR48  [NH2]   2.77  A   iaD53  [OD2]
pbQ6   [NE2]   2.92  A   iaW60  [O   ]
pbD32  [OD1]   2.59  A   iaW60  [NE1]
Salt bridges
paD14  [OD2]   2.85  A   iaK34  [NZ ]
paD14  [OD1]   3.70  A   iaK34  [NZ ]
paR48  [NH1]   3.05  A   iaD53  [OD1]
paR48  [NH2]   3.17  A   iaD53  [OD1]
paR48  [NH2]   2.77  A   iaD53  [OD2]
```

```
In HLA-A2:
Hydrogen bonds
paY27  [OH ]   3.30  A   iaY63  [OH ]
paQ32  [NE2]   2.79  A   iaD53  [OD1]
paR35  [NH1]   2.99  A   iaD53  [OD2]
paR48  [NE ]   2.84  A   iaD53  [OD1]
pbQ6   [OE1]   2.83  A   iaH31  [NE2]
pbQ6   [NE2]   2.83  A   iaW60  [O   ]
pbD32  [OD2]   2.71  A   iaW60  [NE1]
Salt bridges
paR35  [NH1]   3.92  A   iaD53  [OD1]
paR35  [NH1]   2.99  A   iaD53  [OD2]
paR48  [NE ]   2.84  A   iaD53  [OD1]
paR48  [NH1]   3.42  A   iaD53  [OD1]
paR48  [NH1]   3.77  A   iaD53  [OD2]
```

pMHC-II

```
In Chicken BL2-01901:
Hydrogen bonds between pab and ia domains
paQ12  [NE2]   3.00 A   iaP33  [O   ]
paQ23  [NE2]   2.81 A   iaY54  [O   ]
paQ23  [OE1]   3.00 A   iaY54  [N   ]
paE32  [OE1]   2.72 A   iaY55  [OH  ]
pbR30  [NH2]   2.98 A   iaD59  [O   ]
No salt bridges found

In H2-Ag7:
Hydrogen bonds between pab and ia domains
paQ12  [NE2]   3.49 A   iaP33  [O   ]
paQ23  [NE2]   2.73 A   iaF54  [O   ]
paQ23  [OE1]   3.16 A   iaF54  [N   ]
paE32  [OE1]   3.64 A   iaS53  [N   ]
paT90b [O   ]  3.84 A   iaE3   [N   ]
paN90c [ND2]   3.73 A   iaW85  [O   ]
pbK8   [NZ  ]  3.29 A   iaN57  [O   ]
pbQ6   [NE2]   3.19 A   iaH60  [O   ]
pbR30  [NH2]   2.77 A   iaD59  [O   ]
pbR30  [NH2]   2.78 A   iaD59  [OD2]
Salt bridges between pab and ia domains
pbR30  [NH1]   3.63 A   iaE3   [OE2]
pbR30  [NH2]   2.78 A   iaD59  [OD2]

In HLA-DR1:
Hydrogen bonds
paE23  [OE1]   3.02 A   iaF54  [N   ]
paG30  [O   ]  2.73 A   iaR63  [NH2]
paE32  [OE1]   3.80 A   iaV53  [N   ]
paR48  [NH2]   3.38 A   iaY67  [OH  ]
paT90b [O   ]  3.36 A   iaV3   [N   ]
pbK8   [NZ  ]  2.88 A   iaR57  [O   ]
pbQ30  [NZ2]   3.38 A   iaD59  [O   ]
No salt bridges found
```

Supplementary file 2C

Residues that are part of the interface between pMHC ia and ib domains

Residues in the ia domain:

ia	Shark UAA	BSA % / Å²	H/S	$\Delta^i G$	pMHC-I Carp	pMHC-I Frog	pMHC-I Chick	pMHC-I HLA-A2	pMHC-II Chick	pMHC-II H2-Ag7	pMHC-II HLA-DR1
ia6	*n-*				K+(HS)	V+	*k-*	K+	*e-*	*q-*	*e-*
ia8	Q	70% / 64	H	-0.68	Q+(H)	K+(S)	Q+(H)	Q+(H)	A+	*t-*	*t-*
ia9	V	7% / 0.9		-0.01	*v-*	V+	V+	V+	*l-*	*v-*	*v-*
ia10	Y	90% / 77	H	0.76	Y+(H)	T+(H)	Y+(H)	Y+(H)	F+	F+	L+
ia11	T	38% / 9		-0.10	S+(H)	T+	S+(H)	S+(H)	P+(H)	P+(H)	T+
ia12	Y	64% /108	H	0.71	H+(HS)	A+	R+(HS)	R+(H)	A+(H)	K+(HS)	N+
ia13	K	8% / 9		0.15	Y+	E+	F+	H+	E+(H)	S+	S+
ia14	L	21% / 25		0.40	P+	P+	P+	P+	A+	P+	P+
ia15	*i-*				G+(H)	*v-*	A+(H)	*a-*	V+(H)	*v-*	*v-*
ia16	*k-*				E+	*d-*	S+(H)	*e-*	S+(H)	L+	*e-*
ia17	*e-*				Y+(H)	*f-*	*a-*	*n-*	*l-*	*l-*	*l-*
ia22	V	71% / 26		0.41	T+(H)	E+	V+	*f-*	*v-*	*t-*	*v-*
ia24	L	100% / 29		0.47	I+	I+	N+(H)	N+(H)	I+	I+	I+
ia26	H	69% / 26		0.14	Y+	Y+	F+	Y+	Y+	F+	F+
ia28	K	29% / 22		-0.76	*s-*	Y+(H)	*a-*	S+	*d-*	*d-*	*d-*
ia50	*t-*				Q+	K+	*q-*	*e-*	*y-*	*y-*	*s-*
ia52	*s-*				*t-*	T+	*s-*	*s-*	*s-*	T+	T+
ia55	*s-*				*a-*	*s-*	*s-*	*s-*	Y+	*l-*	L+
ia57	*e-*				*e-*	*q-*	*n-*	S+	R+	*n-*	*r-*
ia63	*k-*				*h-*	T+	Q+	Y+	R+(H)	H+	R+(H)
ia65	T	68% / 17		0.08	T+	S+	L+	L+	F+	L+	F+
ia67	Y	48% / 34		0.34	S+	*h-*	H+	Y+	Y+(H)	Y+(H)	Y+(H)
ia69	E	6% / 8		-0.07	*s-*	*h-*	*d-*	*e-*	*p-*	*t-*	*p-*
ia74	*s-*				K+	*k-*	*s-*	*e-*	*r-*	*d-*	*t-*
ia95	*l-*				*w-*	D+(HS)	W+	*w-*	*w-*	*w-*	*w-*
ia95a	D	23% / 27	HS	-0.33	E+(HS)	D+(HS)	D+(HS)	*d-*	*e-*	*e-*	*e-*
ia95b	R	9% / 8	H	-0.10	S+	--	P+(H)	*r-*	*p-*	*p-*	*f-*
ia95c	Y	67% /158	H	1.42	N+(H)	--	E+	D+(H)	*e-*	*e-*	*d-*
ia95d	--				M+(H)	--	--	M+(H)	--	--	R+(HS)

Residues in the ib domain:

	Shark UAA	BSA			*Other pMHC structures:* pMHC-I				pMHC-II		
ib		% / Å²	H/S	ΔⁱG	Carp	Frog	Chick	HLA-A2	Chick	H2-Ag7	HLA-DR1
ib3	*v-*				*v-*	*v-*	E+	*d-*	*v-*	*e-*	*v-*
ib6	*s-*				Q+	*h-*	E+	*k-*	K+	N+	K+
ib7	*v-*				*v-*	*v-*	V+	*t-*	*v-*	*v-*	*v-*
ib8	T	17% / 10		0.16	S+	R+	R+(H)	H+	R+(H)	A+	T+
ib10	T	8% / 4		-0.02	L+	*s-*	W+(H)	T+(H)	*s-*	*s-*	Y+
ib11	*s-*				Q+(H)	*d-*	*g-*	*h-*	*a-*	*l-*	*p-*
ib12	V	68% / 35		0.57	K+(HS)	H+	K+(S)	H+(H)	nd	*s-*	*s-*
ib13	R	51% / 93	HS	-1.37	*d-*	*q-*	*e-*	*a-*	nd	*r-*	*k-*
ib17	--				--	*d-*	*d-*	*d-*	nd	*l-*	L+
ib24	S	92% / 10	H	0.07	T+	R+	S+	R+(H)	*a-*	V	*v-*
ib26	V	29% / 9		0.15	H+(H)	H+	R+	W+(H)	Y+	S+	S+
ib28	T	70% / 34	H	0.32	T+(H)	Y+	H+(H)	L+	T+(H)	T+	S+
ib29	G	43% / 12		0.20	G+	*g-*	G+	*s-*	G+	D+(S)	G+
ib52	T	38% / 27	H	-0.08	*g-*	*k-*	G+(H)	V+	*t-*	*t-*	*t-*
ib53	G	12% / 3		0.05	E+(HS)	E+(HS)	G+	E+(H)	*d-*	*q-*	*g-*
ib54	V	16% / 10		-0.11	L+	I+	I+	T+	*v-*	*l-*	*l-*
ib55	R	82% / 79		-0.66	L+	L+	V+	R+(H)	M+	I+	I+
ib56	P	71% / 72	H	0.70	P+(H)	P+(H)	P+(H)	P+(H)	Q+(H)	R+	Q+(H)
ib57	N	82% / 37		-0.07	N+	N+	N+(H)	A+(H)	N+(H)	N+(H)	N+(H)
ib58	H	70% /122		0.95	E+(HS)	P+	G+	G+	G+	G+	G+
ib59	D	11% / 10		-0.17	D+	D+	D+(HS)	D+	D+(H)	D+(HS)	D+(H)
ib61	S	11% / 1		-0.01	S+	*s-*	T+(H)	*t-*	*t-*	T+(H)	T+
ib63	Q	98% / 31		-0.36	Q+(H)	Q+	H+	Q+(H)	Q+(H)	Q+(H)	Q+
ib65	H	54% / 30	H	-0.83	M+	R+(HS)	W+	W+	*l-*	*l-*	L+
ib67	S	19% / 4		0.02	*t-*	*t-*	*t-*	*a-*	*v-*	*m-*	*m-*
ib72	*p-*				*p-*	*p-*	*p-*	*s-*	*p-*	*p-*	P+
ib73	*n-*				*d-*	*s-*	*g-*	*g-*	*r-*	*h-*	R+
ib74	*s-*				*e-*	*e-*	*d-*	*q-*	*r-*	*q-*	S+
ib77	*k-*				*r-*	*s-*	*k-*	*r-*	*s-*	*v-*	V+
ib94	*f-*				*t-*	*v-*	*s-*	*r-*	*a-*	*e-*	E+(HS)
ib95	*y-*				*e-*	*w-*	*w-*	*w-*	*w-*	*w-*	W+
ib95d	--				I+	--	--	--	--	--	--
ib95e	--				T+(H)	--	--	--	--	--	--
ib95f	--				N+(H)	--	--	--	--	--	--
ib95g	--				F+	--	--	--	--	--	--

Supplementary file 2D

Summary of predicted hydrogen bonds and salt bridges between pMHC ia and ib domains

pMHC-I

```
In Shark UAA:
Hydrogen bonds
iaQ8    [NE2]  3.54 A  ibT52 [O  ]
iaY10   [OH ]  2.66 A  ibP56 [O  ]
iaY12   [O  ]  3.06 A  ibT28 [OG1]
iaD95a [OD1]  2.99 A  ibR13 [NH2]
iaD95a [OD2]  3.35 A  ibR13 [NH1]
iaR95b [O  ]  3.57 A  ibR13 [OG ]
iaY95c [OH ]  3.19 A  ibS24 [OG ]
iaY95c [OH ]  2.78 A  ibH65 [ND1]
Salt bridges
iaD95a [OD1]  2.99 A  ibR13 [NH2]
iaD95a [OD1]  3.91 A  ibR13 [NH1]
iaD95a [OD2]  3.54 A  ibR13 [NH2]
iaD95a [OD2]  3.35 A  ibR13 [NH1]
```

```
In Carp UAA:
Hydrogen bonds
iaK6    [NZ ]  2.93 A  ibE53 [OE1]
iaK6    [NZ ]  3.22 A  ibE53 [OE2]
iaQ8    [NE2]  3.42 A  ibE53 [OE2]
iaY10   [OH ]  2.66 A  ibP56 [O  ]
iaS11   [O  ]  2.97 A  ibQ63 [NE2]
iaH12   [OD1]  2.85 A  ibE58 [OE2]
iaH12   [OD2]  3.62 A  ibH26 [NE2]
iaH12   [O  ]  2.80 A  ibT28 [OG1]
iaH12   [OH ]  3.53 A  ibQ63 [NE2]
iaG15   [OH ]  2.96 A  ibN101[ND2]
iaY17   [OH ]  3.20 A  ibN101[OD1]
iaT22   [OH ]  3.77 A  ibE58 [OE1]
iaE95a [OE2]  2.89 A  ibQ11 [NZ ]
iaN95c [O  ]  3.63 A  ibQ11 [N  ]
iaN95c [O  ]  3.62 A  ibK12 [N  ]
iaM95d [O  ]  2.98 A  ibT100[OG1]
iaM95d [O  ]  2.83 A  ibN101[N  ]
iaM95d [OXT]  2.79 A  ibK12 [NZ ]
Salt bridges
iaK6    [NZ ]  2.93 A  ibE53 [OE1]
iaK6    [NZ ]  3.22 A  ibE53 [OE2]
iaH12   [ND1]  3.40 A  ibE58 [OE1]
iaH12   [ND1]  2.85 A  ibE58 [OE2]
iaE95a [OE2]  2.89 A  ibK12 [NZ ]
```

```
In Frog UAA:
Hydrogen bonds
iaY10   [OH ]  2.65 A  ibP56 [O  ]
iaY28   [OH ]  2.56 A  ibE53 [OE2]
iaD95a [OD1]  3.68 A  ibR64 [NH1]
Salt bridges
iaK8    [NZ ]  3.99 A  ibE53 [OE2]
iaD95a [OD1]  3.68 A  ibR64 [NH1]
```

```
In Chicken BF2*0401:
Hydrogen bonds
iaQ8    [NE2]  2.83 A  ibG52 [O  ]
iaY10   [OH ]  2.66 A  ibP56 [O  ]
iaS11   [O  ]  2.94 A  ibH28 [ND1]
iaR12   [NE ]  2.86 A  ibD59 [OD2]
iaR12   [NE ]  3.51 A  ibT61 [OG1]
iaR12   [O  ]  3.37 A  ibH28 [ND1]
iaA15   [O  ]  2.68 A  ibR8  [NH1]
iaA15   [O  ]  2.78 A  ibR8  [NH2]
iaS16   [OG ]  3.79 A  ibR8  [NH2]
iaN24   [ND2]  3.01 A  ibN57 [OD1]
iaD95a [OD2]  3.90 A  ibW10 [NE1]
iaP95b [O  ]  3.69 A  ibR8  [NH1]
Salt bridges
iaR12   [NE ]  3.51 A  ibD59 [OD1]
iaR12   [NE ]  2.86 A  ibD59 [OD2]
iaR12   [NH2]  3.33 A  ibD59 [OD2]
iaD95a [OD1]  3.97 A  ibK12 [NE ]
```

```
In HLA-A2:
Hydrogen bonds
iaQ8    [NE2]  2.91 A  ibE53 [O  ]
iaQ8    [OE1]  2.91 A  ibR55 [NH1]
iaY10   [OH ]  2.60 A  ibP56 [O  ]
iaS11   [O  ]  3.79 A  ibQ63 [NE2]
iaR12   [NE ]  3.03 A  ibA57 [O  ]
iaR12   [O  ]  3.04 A  ibQ63 [NE2]
iaN24   [ND2]  3.89 A  ibP56 [O  ]
iaN24   [ND2]  3.00 A  ibA57 [O  ]
iaD95c [O  ]  3.20 A  ibH12 [NE2]
iaM95d [O  ]  3.00 A  ibR24 [NH1]
iaM95d [O  ]  2.74 A  ibT10 [OG1]
iaM95d [O  ]  2.98 A  ibW26 [NE1]
No salt bridges found
```

pMHC-II

```
In chicken BL2*01901:
Hydrogen bonds between ia and ib domains
iaP11   [O  ]  3.00 A  ibQ63  [NE2]
iaA12   [O  ]  3.42 A  ibQ63  [NE2]
iaE13   [OE1]  2.74 A  ibT28  [OG1]
iaV15   [O  ]  3.49 A  ibR8   [NH1]
iaV15   [O  ]  3.16 A  ibR8   [NH2]
iaS16   [OG ]  3.14 A  ibR8   [NH1]
iaR63   [NH1]  2.94 A  ibQ56  [OE1]
iaY67   [OH ]  3.69 A  ibF59  [N  ]
iaY67   [OH ]  2.59 A  ibN57  [O  ]

In H2-Ag7:
Hydrogen bonds between ia and ib domains
iaP11   [O  ]  2.82 A  ibQ63  [NE2]
iaK12   [NZ ]  2.62 A  ibD59  [OD2]
iaK12   [NZ ]  3.56 A  ibT61  [OG1]
iaK12   [O  ]  3.17 A  ibQ63  [NE2]
iaY67   [OH ]  3.03 A  ibN57  [O  ]
iaY67   [OH ]  3.73 A  ibD59  [N  ]
Salt bridges between ia and ib domains
iaK12   [NZ ]  3.25 A  ibD29  [OD1]
iaK12   [NZ ]  2.62 A  ibD59  [OD2]

In HLA-DR1:
Hydrogen bonds between ia and ib domains
iaR63   [NH2]  3.03 A  ibQ56  [OE1]
iaY67   [OH ]  3.64 A  ibD59  [N  ]
iaY67   [OH ]  2.46 A  ibN57  [O  ]
iaR95d  [NH1]  3.02 A  ibE94  [O  ]
iaR95d  [NH2]  3.12 A  ibE94  [OE1]
Salt bridges between ia and ib domains
iaR95d  [NH2]  3.12 A  ibE94  [OE1]
```
