# Supplementary material for: Structural Comparison Between MHC Classes I and II; in Evolution, a Class-II-Like Molecule Probably Came First
Source: Front Immunol. 2021 Jun 14;12:621153. doi: 10.3389/fimmu.2021.621153 (PMC8236899; doi:10.3389/fimmu.2021.621153)

# Supplementary file 3

Additional structural analyses of pMHC-I and pMHC-II

**Table of Contents** **Page**


3A: The interface between pMHC ia and pab domains 2

3B: The interface between pMHC ia and ib domains 5

3C: Position of pMHC pab domain cysteine bridges 8

3D: The p33p34 β-bulges and the S2-S3 clefts in the pab β-sheet 21

3E: In pMHC-Is, but not in pMHC-IIs, the ia56 and ia60 residues insert into the pa9 pleat of the pab β-sheet 23

## Supplementary file 3A

### The interface between pMHC ia and pab domains

Comparison of the ia-to-pab interfaces of pMHC-I structures for shark UAA (PDB accession 5KF5), carp UAA (5Y91), frog UAA (6A2B), chicken BF2*0401 (4E0R), and HLA-A2 (3PWN) with each other (a) and with pMHC-II structures for chicken BL2*01901 (6KVM), mouse H2-Ag7 (1F3J), and HLA-DR1(1AQD) (b). The ib domains and peptide ligands are not shown. Presentations are in transparent cartoon format with the sidechains of highlighted residues shown in sticks format. Boxed labels indicate the respective residues in the compared five pMHC-I or three pMHC-II structures. Coloring of highlighted residues is in element color: red for O, blue for N, gold for S, and individual colors for C.

Figure (a) highlights all the residues that substantially contribute to the ia-to-pab interface (see Supplementary files 2A and 2B) by means of their sidechains and have an interesting conservation pattern (Supplementary file 1A). In addition, residue pb(A/S/T)35 is shown because it represents another pMHC-I specific feature of the pa9 pleat close to the ia-to-pab contact region. The superposition of the pMHC-Is was based on superimposing their pab domains. For the individual pMHC-Is different colors of gray are used for most of the pab domain residues while the pa9 pleat top ridge residues are in yellow and the bottom ridge residues are in green. Dashed yellow lines indicate polar contacts.

Figure (b) is similar to (a), except that in addition three pMHC-II structures have been superimposed (based on ia domain superimposition) and only interface residues that appear to have predated MHC-I/MHC-II evolutionary separation are highlighted. The pMHC-II structures are shown in individual pinkish colors.

Comparison between (a) and (b) shows that in evolution the ia domains did not change much in position after the evolutionary separation of MHC-I and MHC-II, with residues such as pbQ6 and ia(F/Y)62 remaining at very similar positions. However, in MHC-I evolution, a number of specific pab domain residues were acquired at the ridges of the pa9 pleat and the MHC-I ia domain ($\beta_2$-m) acquired the specific iaF56 and iaW60 residues that insert into the pab pa9 pleat. Quite interesting is how in pMHC-IIs the pb(E/Q)32 sidechains lay across the pa9 pleat groove whereas in pMHC-Is, with the exception of frog pUAA, the pbD32 sidechain points away from the pab domain to make a hydrogen bond with the iaW60 sidechain. We speculate that the orientation of pMHC-I pbD32 is induced by binding of $\beta_2$-m to the heavy chain and that this, somehow, through unknown long-distance structural changes, may be involved in the synergistic binding between $\beta_2$-m, HC, and peptide ligand. Some residues like for example pbM8 are well conserved among the MHC-I sequences but their sidechains can have different orientations (a).

# Supplementary file 3A(a)

The interface between pMHC ia and pab domains; superposition of representative pMHC-I structures

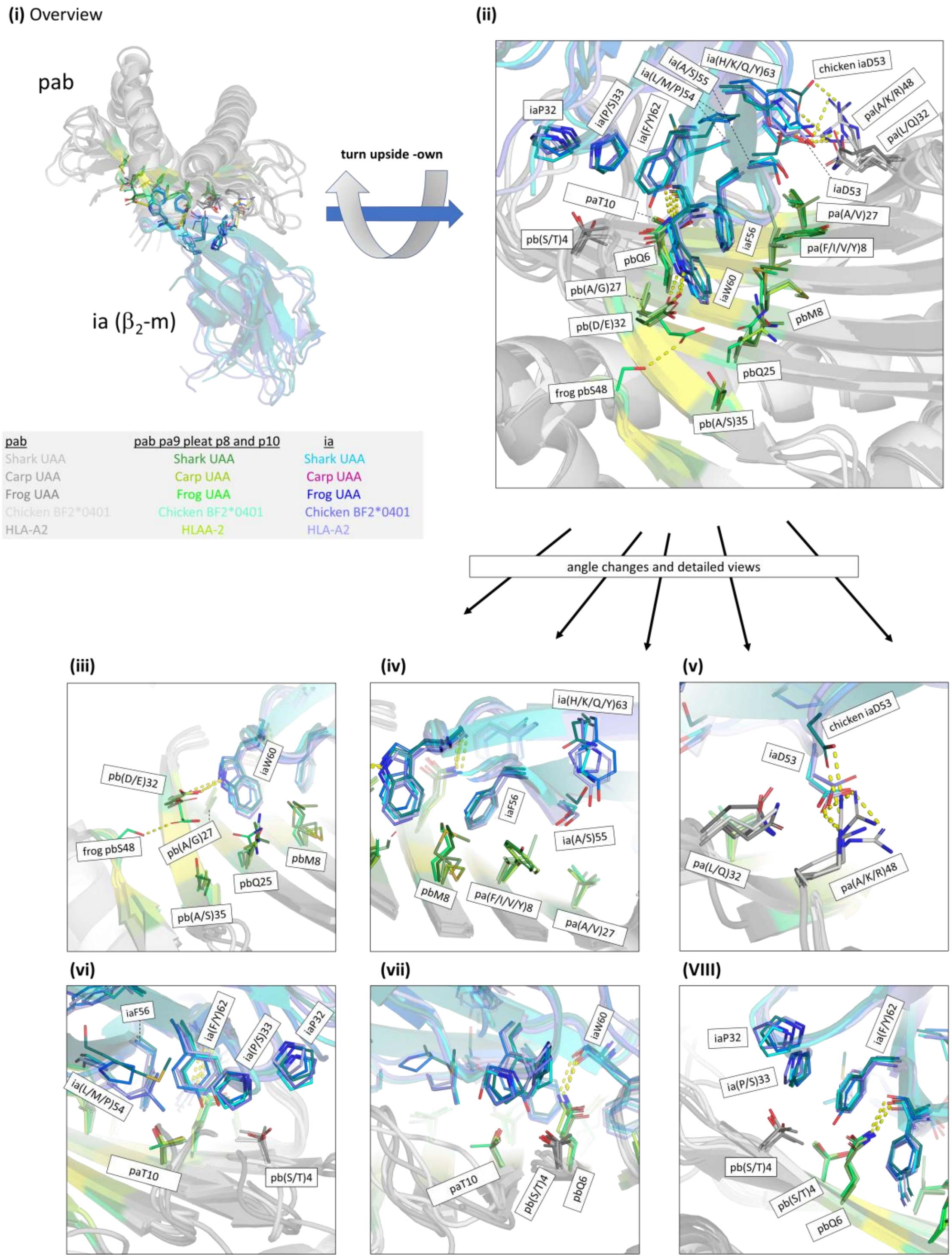

# Supplementary file 3A(b)

The interface between pMHC ia and pab domains; superposition of representative pMHC-I and pMHC-II structures

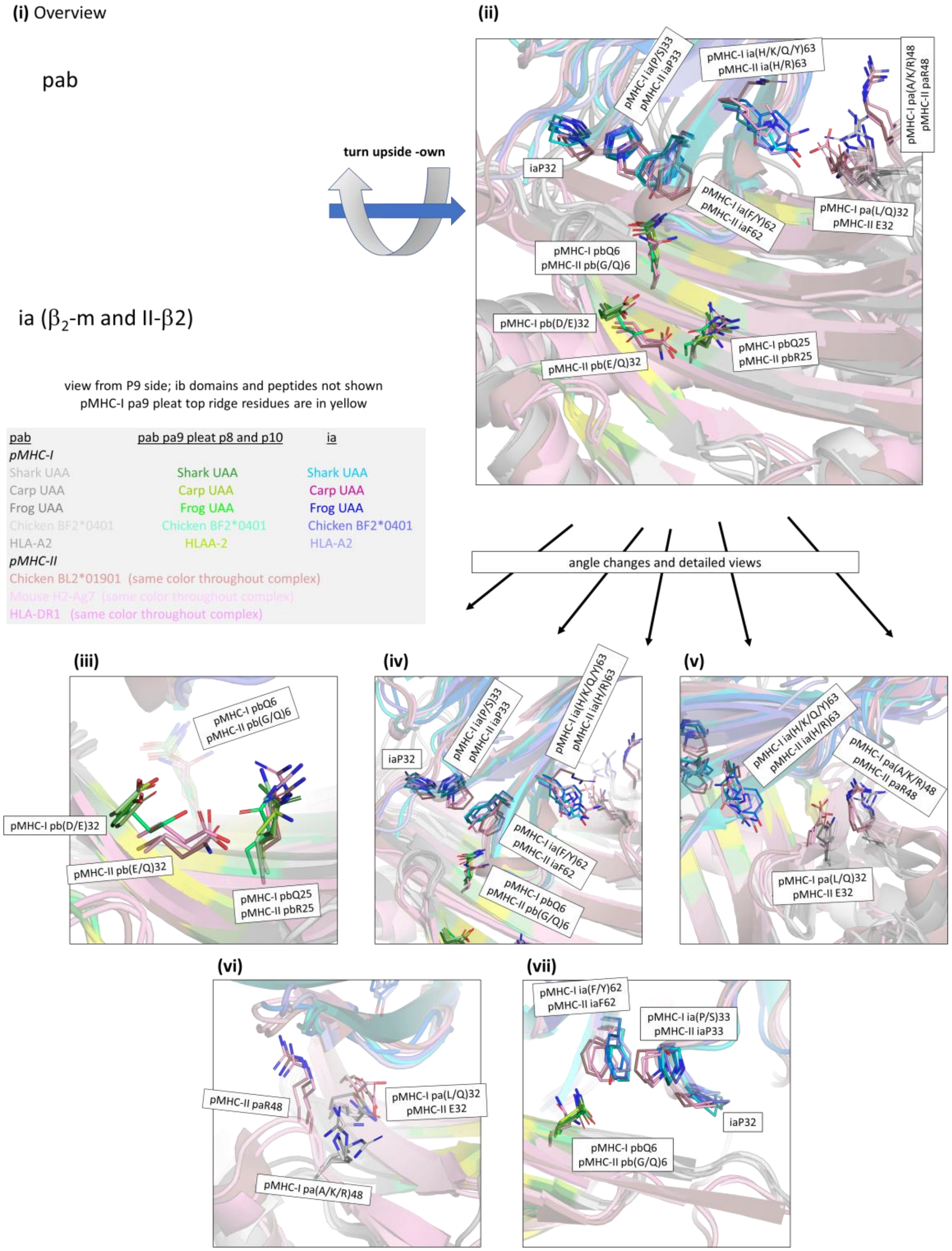

## Supplementary file 3B

The interface between pMHC ia and ib domains

Comparison of the ia-to-ib interfaces of pMHC-I structures for shark UAA (PDB accession 5KF5), carp UAA (5Y91), frog UAA (6A2B), chicken BF2*0401 (4E0R), and HLA-A2 (3PWN) with each other (a-i, a-ii) and together with pMHC-II structures for chicken BL2*01901 (6KVM), mouse H2-Ag7 (1F3J), and HLA-DR1(1AQD) (a-iii, a-iv, b). The peptide ligands are not shown. Presentations are in transparent cartoon format with the sidechains of highlighted residues shown in sticks format. For some highlighted residues also the main chains are shown in sticks format. Coloring of highlighted residues is in element color: red for O, blue for N, gold for S, and individual colors for C.

(a-i) Superposition of five representative pMHC-I structures, based on superimposing of $\beta_2$-m. The IgSF domains are colored per pMHC-I.

(a-ii) Most parts of the pMHC-I complexes as shown in (a-1) were removed to show how the $\alpha$3 domain $\beta$-strands S4 and S5 region interact with the $\beta_2$-m domain $\beta$-strands S1 and S2 region; this forms the major contact between the domains. Considerable differences in the orientation of the $\alpha$3 domain $\beta$-strands S4 and S5 region is observed between the different pMHC-Is.

(a-iii) Same figure as (a-i), with the addition of three pMHC-II structures; superposition based on superimposing of ia domains.

(a-iv) Same type of figure as (a-ii). The orientation of the pMHC-II ib domain $\beta$-strands S4 and S5 region differs somewhat from their organization in pMHC-Is.

(b) Orientations of conserved residues at the ia-ib interfaces. For ib52, ib53, ib56, and ib57 both sidechains and main chains are shown in sticks format, while for the other highlighted residues only the sidechains are shown in sticks format. Numbers indicate as follows. (1) Many, though not all, MHC-II sequences possess iaY67 [Supplementary file 1A(b)] and in mammals and chicken this tyrosine makes a hydrogen bond with the main chain of the ib57 residue. Given that also MHC-II sequences in chondrichthyan fish like shark possess iaY67 [Supplementary file 1A(b)], this interaction may be ancestral to the MHC-II lineage. (2) An ia(I/L)24 residue was probably ancestral to the $\beta$2-m/II-$\alpha$2 lineage although not perfectly conserved in either MHC-I or MHC-II [Supplementary file 1A(b)], and if present forms part of the ia-ib interface. In chicken and mammalian pMHC-I the residue has been replaced by an asparagine [Supplementary file 1A(b)], which in chicken pBF2*0401 and pHLA-A2 make a polar contact with the ib57 sidechain or main chain, respectively. (3) Only among MHC-I sequences the iaY10 residue is well conserved [Supplementary file 1A(b)] and makes a polar

contact with the main chain of the ib56 residue, which in MHC-I is a well conserved proline (better shown in the main text Fig. 9). (4) Residue iaQ8 is rather characteristic for $\beta_2$-m sequences but is not perfectly conserved [Supplementary file 1A(b)] and makes varying contributions to the $\alpha$3 to $\beta_2$-m interface in different pMHC-Is (see also Supplementary file 2D): in shark pUAA and chicken pBF2*0401, it makes a hydrogen bond with the ib52 main chain; in carp pUAA, it makes a hydrogen bond with the ibE53 sidechain; in pHLA-A2, it makes a hydrogen bond with the ib53 main chain and also, not shown here, with the ibR55 sidechain. (5) Among pMHC-Is, a stacked ring contact between I-$\alpha$3 domain ibP56 and $\beta_2$-m domain ia(F/H/Y)26 is well conserved.

## Supplementary file 3B (*continued*)

**(a)** The major contact between the ia and ib domains involves their b-strand regions S1 + S2 and S4 + S5, respectively, with substantial differences in those ib S4 + S5 orientations between pMHCs

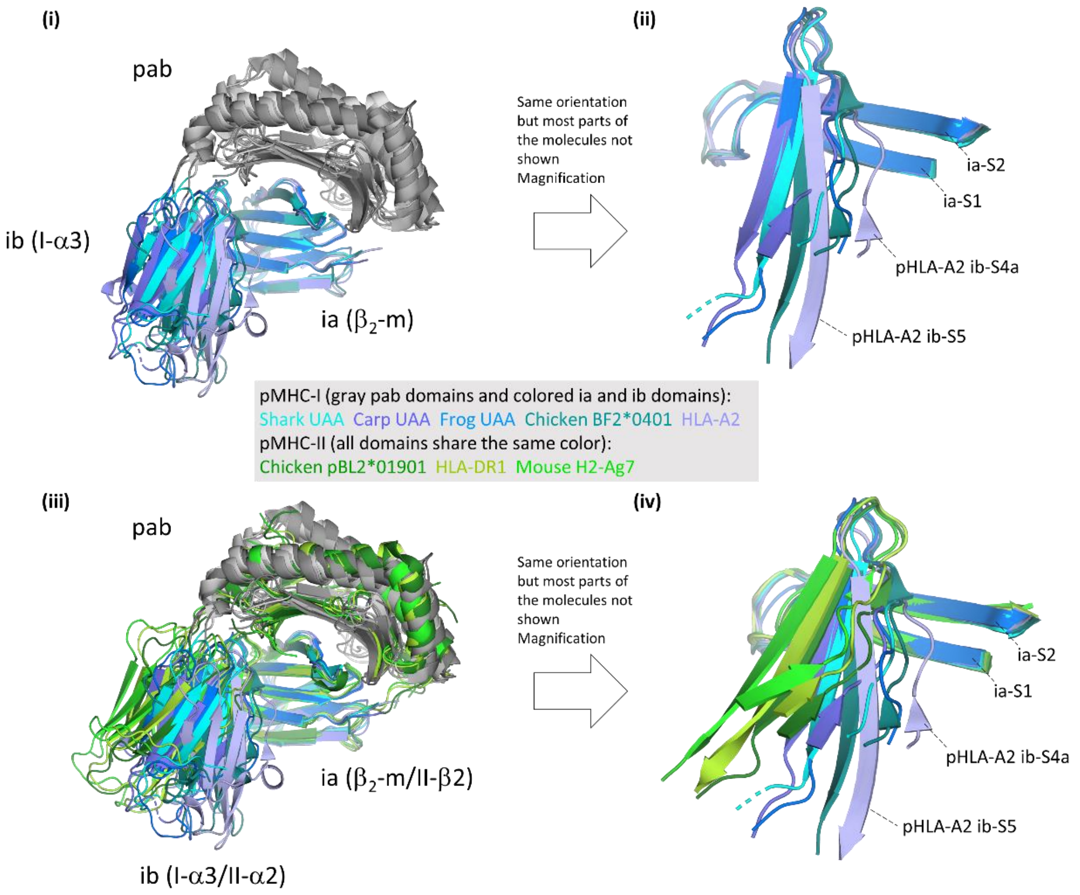


**(b)** Orientations of conserved residues at the ia-ib interfaces

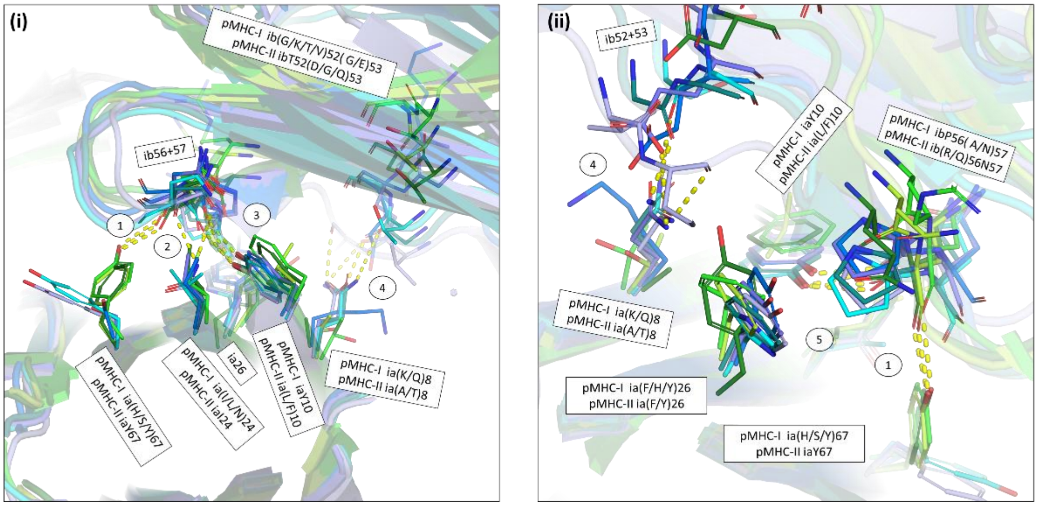

**Supplementary file 3C**

Position of pMHC pab domain cysteine bridges

The pC11-pC74 cysteine bridge inherited from the MHC homodimer ancestor has been well conserved in extant MHC-I $\alpha$2 domains and MHC-II $\beta$1 domains (the pb domains) but was lost in all MHC-I $\alpha$1 domains and most MHC-II $\alpha$1 domains (the pa domains) (see Supplementary file 1A). The reason for the apparent redundancy of the pa domain cysteine bridge, compared to the assumed ancestral MHC homodimer situation, probably has been the stabilization of the pab $\beta$-sheet region including the pa11 position by a reorientation of the ia domain (represented by $\beta_2$-m in class I and II-$\alpha$2 in class II). Figures (a) and (b) show how the ia domains in pMHC-I and pMHC-II, respectively, are in contact with the pa11 residue (the pa11 and pa74 residues are in orange), whereas the pMHC-I and pMHC-II pab b-sheet regions including the pbC11 residue of the pbC11-pbC74 cysteine bridge (cysteines in cyan) are not supported by an IgSF domain. The different domains and peptide ligand are indicated with individual colors.

(a) The positions of the pbC11-pbC74 cysteine bridge (cyan) and paW11 and paG74 (orange) in shark pUAA. (i) and (ii) are views from queer below, (iii) is a view from the side, and (iv) is a view from queer above. Presentation formats are: (i) surface format (paW11 can't be seen in such presentation); (ii) cartoon format with p11 and p74 residues highlighted in spheres format; (iii) and (iv) pab domain in cartoon format with p11 and p74 residues highlighted in spheres format, and IgSF domains in surface format.

(b) The positions of the pbC11-pbC74 cysteine bridge (cyan) and pa11 and pa74 residues (orange) in chicken pMHC-II (BL2*01901) (i) and pHLA-DR1 (ii). Presentation styles are as used in (a-ii). In (i) and (ii), chicken pBL2*01901 residues paY11 and paG74, and pHLA-DR1 residues paY11 paD74, respectively, are shown in orange spheres format. In both pMHC-II structures, the paY11 residue is in direct contact with the II-$\alpha$2 domain.

## Supplementary file 3C (*continued*)

**(a)** The positions of the pvC11-pbC74 cysteine bridge (cyan) and paW11 and paG74 (both orange) in shark pUAA

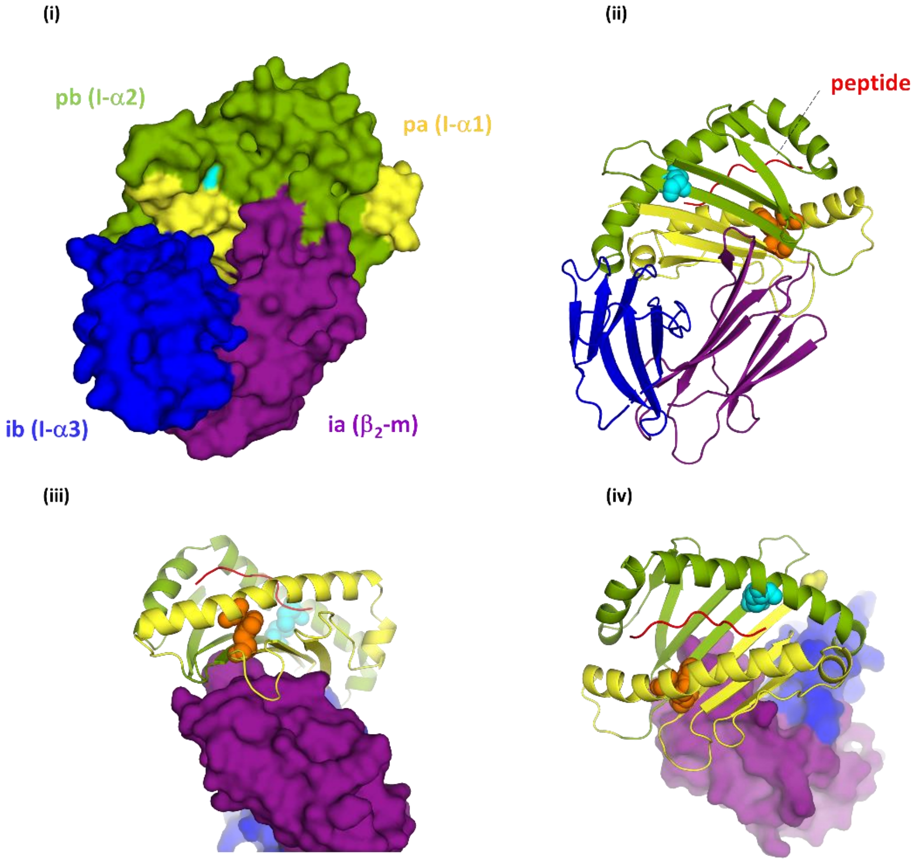


**(b)** The positions of the pvC11-pbC74 cysteine bridge (cyan) and pa11 and pa74 (both orange) in chicken pMHC-II (pBL2*01901) and pHLA-DR1

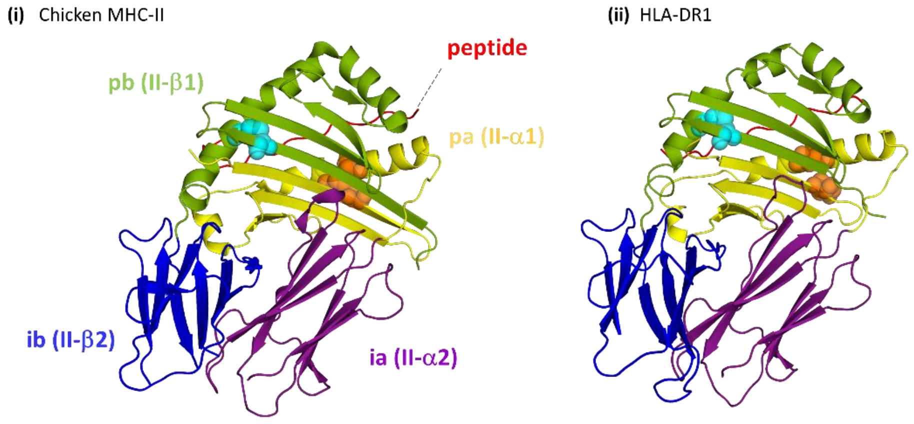

## Supplementary file 3D

The p33p34 β-bulges and the S2-S3 clefts in the pab β-sheet

This figure is similar to main text Fig. 5C (a-iii) and –(b-iii), except that more structures are shown. The main chains of the pab β-sheets are shown from above for pMHC-I structures of shark UAA (PDB accession 5KF5), carp UAA (5Y91), frog UAA (6A2B), chicken BF2*0401 (4E0R), and HLA-A2 (3PWN), and for pMHC-II structures of chicken BL2*01901 (6KVM), mouse H2-Ag7 (1F3J), and HLA-DR1(1AQD). Main chains of residues are shown in sticks format and hydrogen bonds between them are shown by dashed red lines; C residue colors are per domain (yellow for pa, green for pb), unless the residue is individually highlighted (as in main text Fig. 5C), while O is in red and N in blue. In pMHC-I pa and pMHC-II pb domains, the β-sheet connection between β-strands S2 and S3 extends to the p22-p38 pair, whereas pMHC-I pb and pMHC-II pa have an "S2-S3 cleft" meaning that the S2-S3 β-sheet connection only extends to the p24-p36 pair.

Colors: **pa domain**, **pb domain**, **pa7**, **pa26**, **pa33**, **pa34**, **pb33**, **pb34**, **pb45**

**pMHC-Is**

Shark UAA

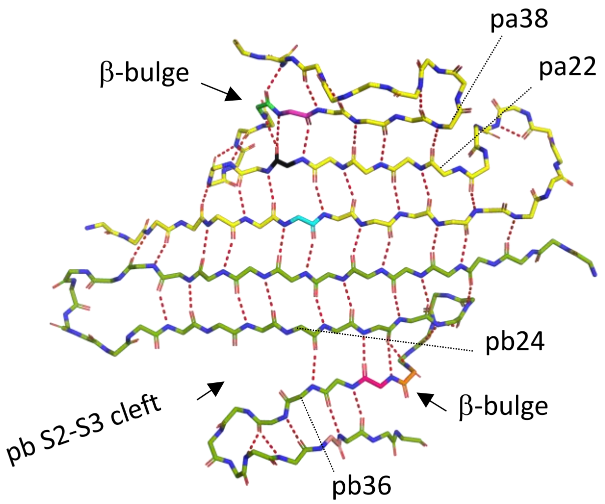


Carp UAA

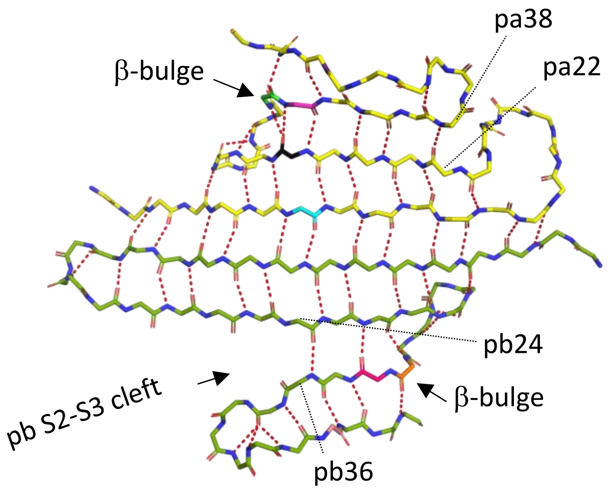

Frog UAA

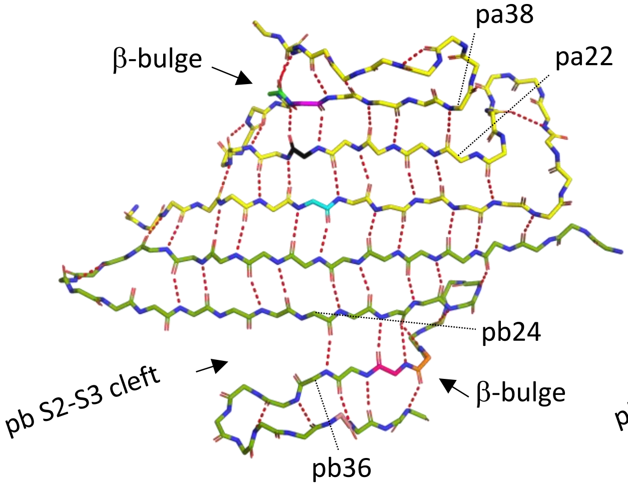


Chicken BF2*0401

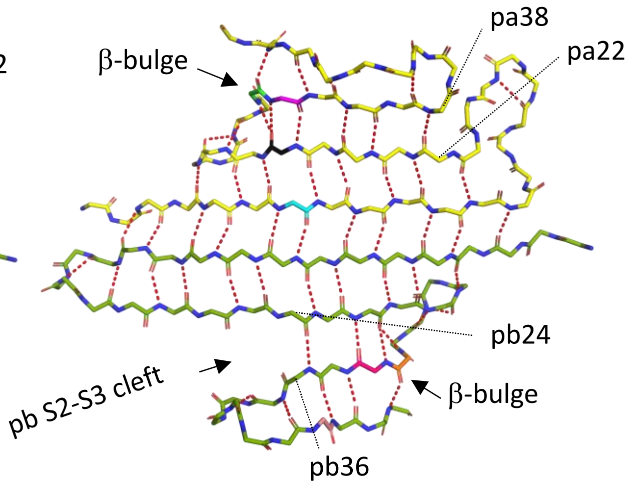


HLA-A2

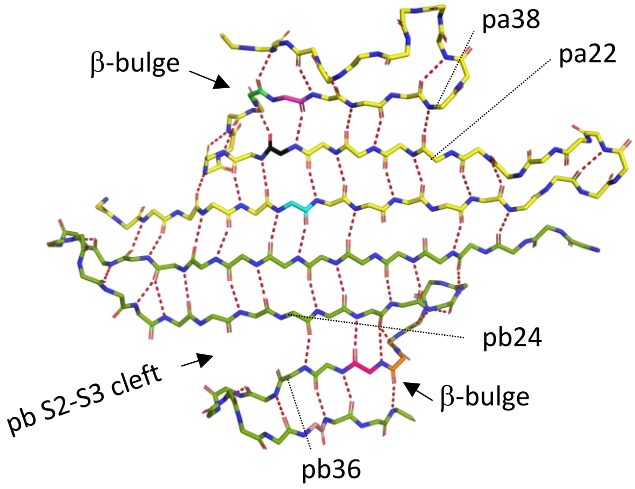

**pMHC-IIs**

Chicken BL2*01901

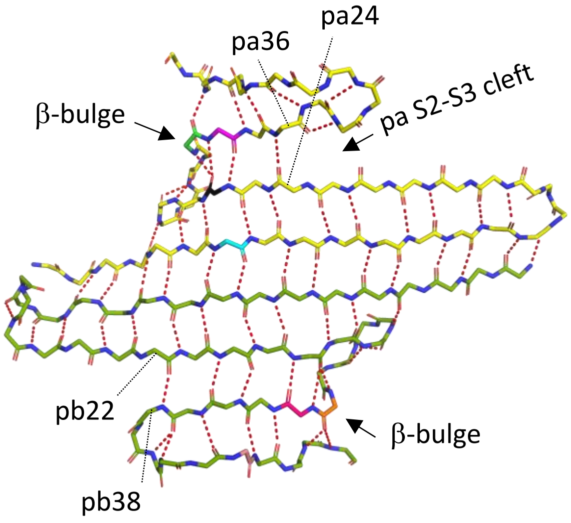


Mouse H2-Ag7

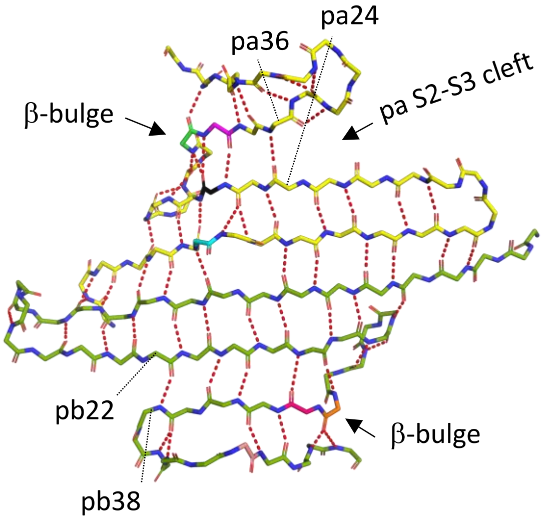


HLA-DR1

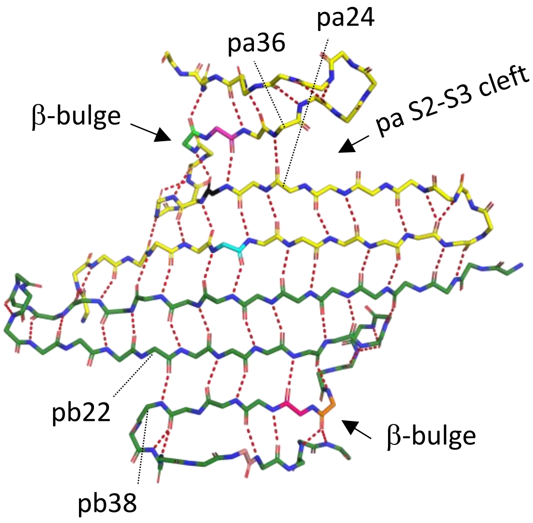

## Supplementary file 3E

In pMHC-Is, but not in pMHC-IIs, the ia56 and ia60 residues insert into the pa9 pleat of the pab β-sheet

Sideviews through the pa9 pleats. This figure is similar to main text Fig. 7 (A-b) and -(B-b), except that more structures are shown. The ia domains, mostly in cyan cartoon format, and the pab domains, mostly in gray ribbon format, are shown for pMHC-I structures of shark UAA (PDB accession 5KF5), carp UAA (5Y91), frog UAA (6A2B), chicken BF2*0401 (4E0R), and HLA-A2 (3PWN), and for pMHC-II structures of chicken BL2*01901 (6KVM), mouse H2-Ag7 (1F3J), and HLA-DR1(1AQD). The ib domains are not shown. The pa9 pleat residues are shown in yellow (top-ridge) and different kinds of green (lower ridges), with the sidechains of the lower ridge residues in element color sticks format. The ia56 and ia60 residues are shown in element color spheres format. Among pMHC-Is, the iaF56 and iaW60 residues are highly conserved, and they consistently insert into the pa9 pleat. Among pMHC-IIs, residues at these positions are not well conserved, and they do not insert into the pa9 pleat.

**pMHC-Is**

Shark UAA

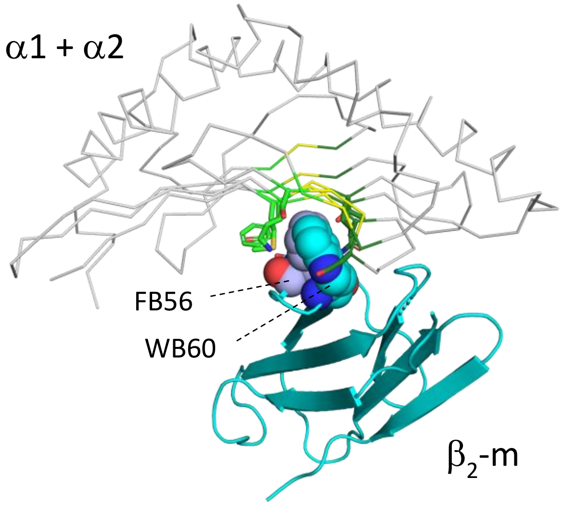


Carp UAA

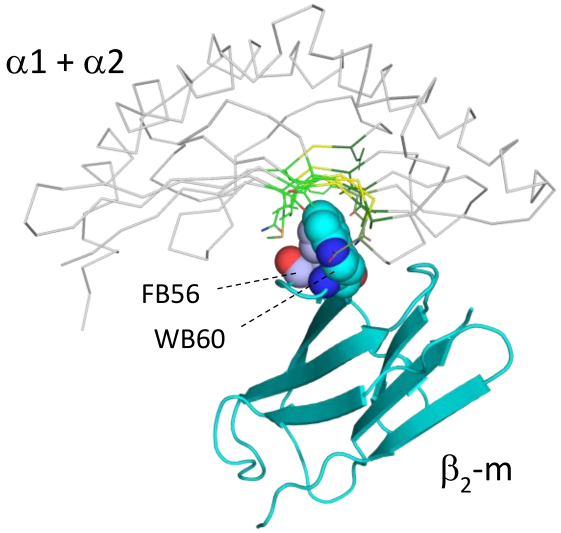

Frog UAA

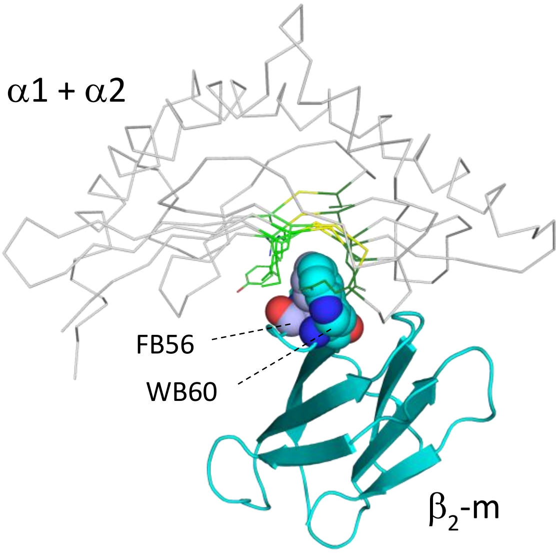


Chicken BF2*0401

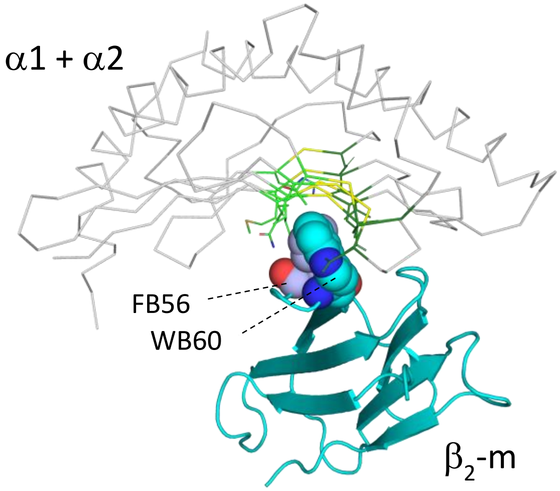


HLA-A2

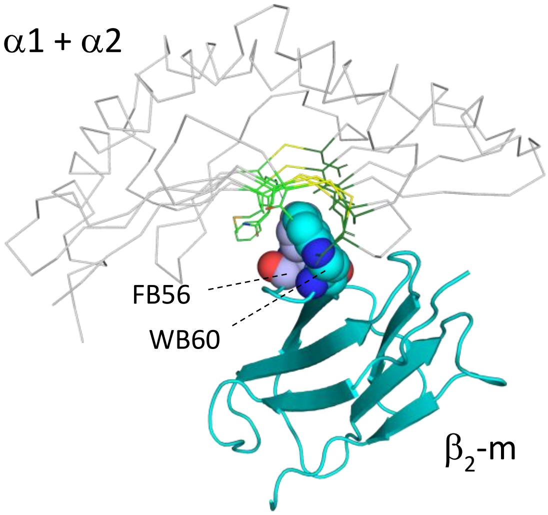

**pMHC-IIs**

Chicken BL2*01901

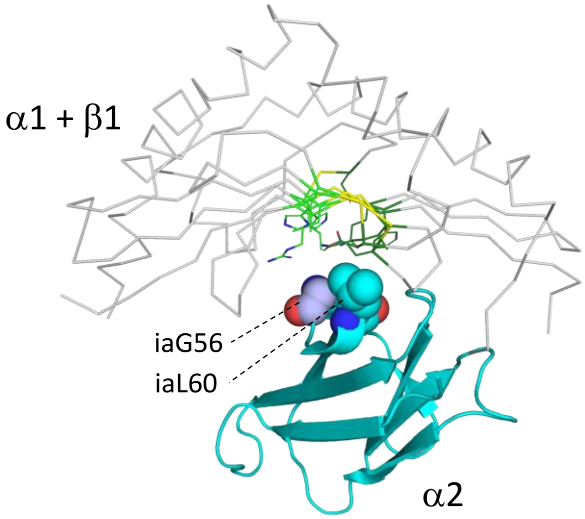


Mouse H2-Ag7

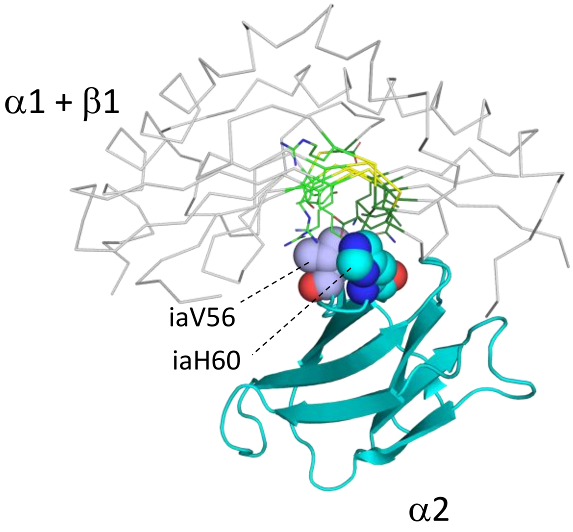


HLA-DR1

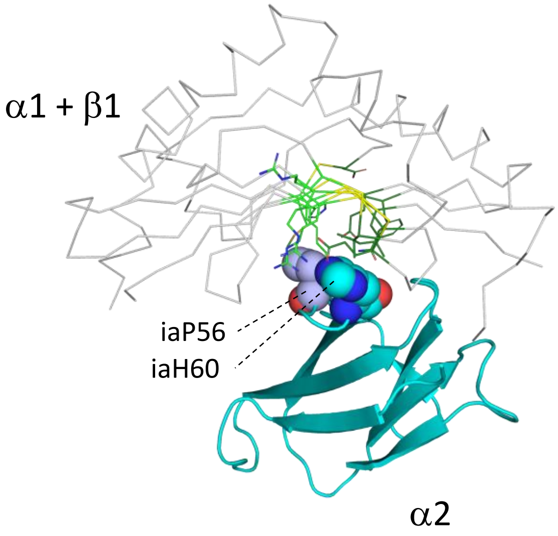

Supplement: Supplementary file 1 [file DataSheet_1.zip › Supplementary File 3.pdf]
