# Supplementary material for: Structural Comparison Between MHC Classes I and II; in Evolution, a Class-II-Like Molecule Probably Came First
Source: Front Immunol. 2021 Jun 14;12:621153. doi: 10.3389/fimmu.2021.621153 (PMC8236899; doi:10.3389/fimmu.2021.621153)

Supplementary file 4

Lungfish MHC-IIabSol sequences

Table of Contents **Page**

Legend to Supplementary files 4A, 4B, and 4C 2

4A: Lungfish MHC-IIabSol sequences, full-length 5

4B: Alignment of deduced MHC-II and lungfish MHC-IIabSol amino acid sequences 6

4C: Phylogenetic trees of the sequences compared in (4B) 8

Legend to Supplementary files 4A, 4B, and 4C

(4A) Lungfish MHC-IIabSol sequences, full-length.

For three different lungfish species we assembled MHC-IIabSol sequences by hand from single read archive (SRA) NCBI databases, namely for slender lungfish (*Protopterus dolloi*; from datasets SRX4717958, SRX8708414, and SRX8647037), West African lungfish (*Protopterus annectens*; from dataset SRX4718670), and Australian lungfish (Neoceratodus forsteri; from dataset SRX1823846). For South American lungfish (Lepidosiren paradoxa) sequence, a similar sequence was retrieved from an NCBI transcriptome shotgun assembly (TSA) database (GenBank accession GEHZ01024954). For slender lungfish we confirmed the MHC-IIabSol sequence experimentally by RT-PCR and sequencing. In the figure, the four sequences are shown as aligned by CLUSTAL 2.1 software (https://www.genome.jp/tools-bin/clustalw). '*' indicates positions which have a single, fully conserved residue, while ':' and '.' indicate full conservation within a 'strong' or 'weaker' group of similar amino acids, respectively. Leader sequences were predicted using SignalP software (http://www.cbs.dtu.dk/services/SignalP/) and are indicated by gray shading. The α1, α2, and α3 domain indications between triangles are based on MHC-I consensus (see Supplementary file 4B). The MHC-IIabSol linker sequence, connecting the IIα and IIβ sequences, in Australian lungfish is quite different from that in the other three lungfishes. Conserved cysteines are shaded in yellow, and MHC-II-characteristic residues for making hydrogen bonds with the peptide ligand main chain are shaded in light blue. The sequences lack putative transmembrane domains and only have a very short extension C-terminal of the α3 domain, which together with the possession of leader sequence predicts that the molecules are secreted.

(4B) Alignment of deduced MHC-II and lungfish MHC-IIabSol amino acid sequences. (a), II-α1 and II-β1 domains; (b), II-α2 and II-β2 domains. The residue numbering used in the present paper for all peptide binding domains and IgSF domains is as indicated above and below the alignments, following the numbering by Saper *et al.* (1991) of HLA-A2 α1 and β_2-m domain residues, respectively. For convenience of the readers, below the alignments also the continued numbering by Saper *et al.* (1991) of HLA-A2 α2 and α3 domain residues is shown as in main text Fig. 3 and Supplementary file 1A. For database accessions of the individual sequences see the end of this figure legend.

Residues in are color-shaded if they display an interesting conservation pattern in MHC-I and/or MHC-II evolution, and are similarly used as in Supplementary file 1A. The color-shading is based on the II-to-I evolution model and our, partially subjective, comparisons of sequences in the present and previous studies (e.g. Dijkstra et al. 2013; Grimholt et al. 2015;

Dijkstra et al. 2018b), and intends to help with the estimation of when in evolution certain residues or "residue-types" were established or fixated. "Residue-types" here refers to the sets of residues indicated above the alignment (unless if characteristic for MHC-I; for that see Supplementary file 1A), which we chose somewhat arbitrarily based on the observed MHC sequence conservation patterns and on similarities between amino acids. Residues or residue-types that, by deduction, were probably present at the respective position in the assumed ancestral homodimer are shaded black. Residues or residue-types that probably were present in early members of, and characteristic for, lineage I-α1+β_2-m/IIα and lineage I-α2+I-α3/IIβ, are shaded dark blue and dark purple, respectively. Residues or residue-types that are characteristic for the I-α1+β_2-m, IIα, I-α2+I-α3, and IIβ lineages are shaded light blue, blue, pink, and purple, respectively. In (1Ab), also some IgSF C1 category sequences of non-MHC sequences are compared, showing that the MHC IgSF residues shaded black are not uncommon among IgSF C1 set sequences. However, the tryptophan at position 95 is characteristic for MHC, and is shaded red. Fonts of residues that are not color-shaded are colored red for basic residues, blue for acidic residues, purple for cysteines, and green residues are more hydrophilic than the orange ones (Hopp and Woods, 1981).

Especially in the α1 domains, around positions pa61 and pa85, the pMHC-IIabSol sequences show features typical for Sarcopterygii (lobe-finned fish plus tetrapods).

(4C) Estimation of evolutionary relationships of lungfish MHC-IIabSol and other MHC-II by Neighbor-Joining method.

(a) The evolutionary history of the II-α1 and II-β1 domain amino acid sequences shown in Supplementary file 4B was inferred using the Neighbor-Joining method (Saitou and Nei 1987). The optimal tree with the sum of branch length = 18.67118712 is shown. The percentage of replicate trees in which the associated taxa clustered together in the bootstrap test (10000 replicates) are shown next to the branches if >50 (Felsenstein 1985). The tree is drawn to scale, with branch lengths in the same units as those of the evolutionary distances used to infer the phylogenetic tree. The evolutionary distances were computed using the Poisson correction method (Zuckerkandl and Pauling 1965) and are in the units of the number of amino acid substitutions per site. The analysis involved 46 amino acid sequences. All ambiguous positions were removed for each sequence pair. There were a total of 100 positions in the final dataset. Evolutionary analyses were conducted in MEGA7 (Kumar *et al.* 2016).

(b) The evolutionary history of the II-α2 and II-β2 domain amino acid sequences shown in Supplementary file 4B was inferred and displayed as in (a). The optimal tree with the sum of branch length = 12.39962943 is shown. The analysis involved 46 amino acid sequences. There were a total of 96 positions in the final dataset.

(c) The evolutionary history of (artificial) MHC-II $\alpha1+\alpha2+\beta1+\beta2$ combinations of MHC-IIa and MHC-IIb amino acid sequences from identical or similar species shown in Supplementary file 4B was inferred and displayed as in (a). The optimal tree with the sum of branch length = 7.20196926 is shown. The analysis involved 22 amino acid sequences. There were a total of 392 positions in the final dataset.

A definite conclusion on the precise time of emergence of the MHC-IIabSol lineage would need a more extensive analysis of classical MHC-II sequences in coelacanths and lungfishes. However, our combined sequence data (Supplementary file 4B) and phylogenetic tree data (Supplementary file 4C) support that MHC-IIabSol originated in early Sarcopterygii (lobe-finned fish plus tetrapods), possibly only in Dipnomorpha (lungfishes) as suggested by Supplementary file 4C(c). Outside of lungfishes we have not identified MHC-IIabSol sequences.

References used in this file

Dijkstra JM, Grimholt U, Leong J, Koop BF, Hashimoto K (2013) Comprehensive analysis of MHC class II genes in teleost fish genomes reveals dispensability of the peptide-loading DM system in a large part of vertebrates. BMC Evol Biol 13:260.

Dijkstra JM, Yamaguchi T, Grimholt U (2018) Conservation of sequence motifs suggests that the nonclassical MHC class I lineages CD1/PROCR and UT were established before the emergence of tetrapod species. Immunogenetics 70(7):459-476.

Felsenstein J (1985) Confidence limits on phylogenies: An approach using the bootstrap. Evolution 39(4):783-791.

Grimholt U, Tsukamoto K, Azuma T, Leong J, Koop BF, Dijkstra JM (2015) A comprehensive analysis of teleost MHC class I sequences. BMC Evol Biol 15:32.

Kumar S, Stecher G, Tamura K (2016) MEGA7: Molecular Evolutionary Genetics Analysis version 7.0 for bigger datasets. Mol Biol Evol 33(7):1870-1874.

Saper MA, Bjorkman PJ, Wiley DC (1991) Refined structure of the human histocompatibility antigen HLA-A2 at 2.6 A resolution. J Mol Biol 219(2):277-319.

Saitou N, Nei M (1987) The neighbor-joining method: A new method for reconstructing phylogenetic trees. Mol Biol Evol 4(4):406-425.

Zuckerkandl E, Pauling L (1965) Evolutionary divergence and convergence in proteins. Edited in Evolving Genes and Proteins by V. Bryson and H.J. Vogel, pp. 97-166. Academic Press, New York.

Supplementary file 4A

Lungfish MHC-IIabSol sequences, full-length

```
                                    Leader                              ► a1 domain
Slender_lungfish_MHC-IIabSol        MKTLGKNSKTNLWNG-SMMLWSLITLLLMKTSAGQQGDHILRQSTTCQKD
W.A._Lungfish_MHC-IIabSol           MKILGKNSKTNLWNG-SMMLWSLITLLLMKTSAGKQGDHILRQSTTCQKD
S.A._Lungfish_MHC-IIabSol           MGTLDKHGTSYTWNGNMVLQWSIIALFLLKLSAGQQGDHILRQSTMCQKD
Australian_lungfish_MHC-IIabSol     -----------------MILWSLSILLLLKRSLGQQGDHLLRQNTLCQKD
                                                     :: **:  *:*:* * *:****:***.* ****

Slender_lungfish_MHC-IIabSol        IPSGDFGFYLDDDELFYVDLDTKEVVYRLPEIESVFSFEAQGGLQNLAVC
W.A._Lungfish_MHC-IIabSol           IPSGDFGFYLDDDELFYVDLDTKEVVYRLPEIESVFSFEAQGGLQNLAVC
S.A._Lungfish_MHC-IIabSol           IPSGDFGFYFDGDEIFYVDLDSKEAVYKLPDFEKIMSFEAQRGLQQLAIC
Australian_lungfish_MHC-IIabSol     TPTGEFGFYFDGDEIFYVDQDSKEVVYRLPEFEKAFSFEAQAGLQSIALC
                                     *:*:****:*.**:**** *:**.**:**::*. :***** ***.:*:*

                                                     ◆ a2 domain
Slender_lungfish_MHC-IIabSol        RHNLDIYIKRTNSTSGPIVPPVVKVYPMQHVKPGKPNTLICATEDFFPCV
W.A._Lungfish_MHC-IIabSol           RHNLDIYIKRTNSTSGPIVPPVVKVYPMQHVKPGKPNTLICATEDFFPCV
S.A._Lungfish_MHC-IIabSol           NFNLDMYIKRSKGISGPKVPPEVKVYPSHPVELGKPNILICHAGGTFPCA
Australian_lungfish_MHC-IIabSol     QYNLDVFMKGTKGKSGPKVPPMVKVYPAQSVDLGKPNTLICSMDGFFPPV
                                    ..***:::* ::. *** *** ***** : *. **** ***   . ** .

Slender_lungfish_MHC-IIabSol        LNISWSKNGIPVKEGITVTDFYPGKEFTFKRFAYLTFIPVQGDIYSCNVE
W.A._Lungfish_MHC-IIabSol           LNISWSKNGIPVKEGITVTDFYPGKEFTFKRFAYLTFIPVQGDIYSCTVE
S.A._Lungfish_MHC-IIabSol           LNISWSKNGSPLEEGIVITDFYPGKEFAFERFSYLTFIPDQGDIYSCTVE
Australian_lungfish_MHC-IIabSol     LNVSWSKNDNPVEEGVQITDFYPGEDSAFERFAYLTFIPDQGDIYSCTVE
                                    **:*****. *::**: :******:: :*:**:****** *******.**

                                               ◆ Linker
Slender_lungfish_MHC-IIabSol        HPGLDRPTTVFWQPDNPSGNENKTVSFSNYYK------------------
W.A._Lungfish_MHC-IIabSol           HPGLDRPTTVFWQPDNPSGNENKTVSFSNYYK------------------
S.A._Lungfish_MHC-IIabSol           HPAFDRPTTVFWQAEKSSSYENRTVSFGIHSK------------------
Australian_lungfish_MHC-IIabSol     HPALDTPDTVFWAPKVPSAEENKTSPFSIRSKPRGHTASLRGTESQVFRA
                                    **.:* * **** .. .*. **:* .*.   *

                                              ◆ b1 domain
Slender_lungfish_MHC-IIabSol        ---------SSGVFLHQITADCLYMREGQKLVFLQTTLYNKERQVYFDSR
W.A._Lungfish_MHC-IIabSol           ---------SSGVFLHQITADCLYMREGQKLVFLQTTLYNKERQVYFDSR
S.A._Lungfish_MHC-IIabSol           ---------FNRVHLYQTIVDCLYVNGGQTVILLQTIVYNRKKQLYFDSR
Australian_lungfish_MHC-IIabSol     LQDTQKASILSGVYLLQQISDCLYKNDGQDVVLLYRVIYNKEEQLYFDSR
                                              . *.* *   **** . ** :::*   :**::.*:*****

Slender_lungfish_MHC-IIabSol        IGKFIGITEFGKIEAEYWNNDTAYLEKMRAAIMIFCKHNFDLLNNTIMDR
W.A._Lungfish_MHC-IIabSol           IGKFIGITEFGKIEAEYWNNDTAYLEKMRAAIMMFCKHNFDLLNNTIMDR
S.A._Lungfish_MHC-IIabSol           VGKFIGVTELGKTDAEYFNNNTAYLEQLMEEVVTVCKYNYDIMKVVIVDR
Australian_lungfish_MHC-IIabSol     VGKFIGITEFGKFQAEYWNNDTAYLAQLRASVDTYCRHNYELMKTSVLDR
                                    :*****:**:** :***:**:**** ::   :   *::*:::::  ::**

                                    ◆ b2 domain
Slender_lungfish_MHC-IIabSol        KVIPVVTMQPLRSVIEKHPNLLMCEASGFYPQSIRVTMLKNGEEVTRDIS
W.A._Lungfish_MHC-IIabSol           KVIPVVTMQPLRSVIEKHPNLLMCEASGFYPQSIRVTMLKNGEEVTRDIS
S.A._Lungfish_MHC-IIabSol           KVIPVITVRSLRKVLEKHPNLLMCAASGFYPQSINVTMFMNGQEVTHDIS
Australian_lungfish_MHC-IIabSol     KVSPTVTVQPFRIVKEKRPNLLICATNGFYPQNINVTLLKNGEEVTHDIS
                                    ** *.:*::.:* * **:****:* :.*****.*.**:: **:***:***

                                                                                ◄
Slender_lungfish_MHC-IIabSol        SSGLISNGDWTYQITLLMEFIPKKEDVYGCKVEHSSLNEPMIVEWRMEAYN---
W.A._Lungfish_MHC-IIabSol           SSGLISNGDWTYQITLLMEFIPKKEDVYGCKVEHSSLNEPMIVEWRMEAYN---
S.A._Lungfish_MHC-IIabSol           SSGVISNGDWTYQITLLMEFLPKKEDVYSCKVEHSSLKEPVIVEWRMEASN---
Australian_lungfish_MHC-IIabSol     SSGVISNGDWTYQIILLMEFIPKRADVYSCKVEHSSLKEPLIVDWKYPAAQPSN
                                    ***:********** *****:**: ***.********:**:**:*:  * :
```

Supplementary file 4B(a)

Alignment of deduced MHC-II and lungfish MHC-IIabSol peptide binding domain amino acid sequences

```
Probable ancestral homodimer residues
                                                               N  Q    N                       N         R
                                   t                           E  E   YE                       E         Q         NL
                                G Hs        C                  D  DΦΦ FD            w         WD         K      C  DI
II-a1/I-a1 lineage ancestral residues
                                                                                                                           t  t
                                                      P        D                 R                                        rsn s
MHC-II alpha 1 domain
                                                                 N
                                                                 E                                         N
                                                         Q       DE               LP F                     D
                                .1        .10         .20E       .30   D  .40     I.50        .60          .70         .80      .90
Nurse shark pSa5-1              YLYDF-TQVYFVQQ-RS-----PEKHFDVMEDGDEIFYMDFN-LKKEVARIPEFAH--LY-MQG----GEAGISANI-AIVKNNLKVVMNLSGGT-PEPK
Little skate FL670178           YRHEE-HLVLYLVQD-GS-----PDKQFDMLVDDDEVFYMDFK-LKKEVARIPAFQH--IT-IEG----GEAGITADI-AFAKQNFNVWKNLSHGS-PEPK
Elephant shark AFM88468         YVHDG-SLVWLCQD-EA-----PDKQFDVEVDWDEVYYMDFQ-QKKEIVRLPEFNS--RT-IQG----GEAGVSAQI-ATCKHNLGGWKKISHGDTPQPN
Elephant shark AFM88844         YVHDG-SMVSLCQD-EA-----PDKQFDIEMDWDEVLYMDFE-QKKEIVRIPHFDG--HG-IQG----GEASVSANI-AVCKHNLGGWKKNSQGVIPEPN
Bichir SRAs                     EPHID-INVITCIS-GS-----TETEDEEQVDGEEMFYTDFI-NNKMVITLPNFADPFDV-VPG----WVQTAQANK-QICLSNLDVAIKAEQNP-PEYE
Reedfish XP_028681425           EPHIE-MRVTMCTS-GS-----TETEDELQLDGDEELYADFI-NNKVVMTIPNFVDQFGA-DPG----WMQVAQADK-QVCFNDLGINIKAEKNP-PESK
White sturgeon DR975335         VTHLF-RALVACQT-NG-----TLPEDDYELDDDELFHIDFD-KKEAVQRIPDFAKYWSP-PEG----APARAETDR-QTCINNVAVAAKCHKYP-PERQ
Zebrafish a4                    VVHED-IVMDGCSD-T-------EKEYISVLDGEEMYHTDFS-GKRGEMTLPDFADP-FT-YPG----TYEQSLADY-ETCKHNLDVAAKAYKSP-LEKL
Trout DAA*0101                  VLHTD-IYINGCSD-S-------DGVDMYGLDGEELWYADFN-KKEGVVALPPFADQ-IS-FPG----YYEQAVGNQ-RTCKGDLGVDIKAYKNP-PETI
Stickleback DAA*01              GPHRD-IAITGCSD-S-------DGEDMYGLDGEELWYADFK-HGKGVKPQPSFVDPIEF-QEG----TYELAVGNQ-QICRINLKNRLKGLKDV-PLEK
Coelacanth XP_006014228         TSLLY-DKTLVCQT-AE-----PKVQFNWEIDEDELVRVDID-KQKMEWRLPEFKG--HK-IDS----LVEWTRQNI-PICEHNLDLLIKRTNGL-LGKT
S.A. Lungfish GEHZ01055957      VEHVMMAGTMFCQK-DVR----MPGQYSFEWDKEDILDVDLQ-KKESVYHAPNIQK--FA-YYE-----AQGGLQNM-AVCKHNLDVLI-RTNST-PGPT
Slender lungfish MHC-IIabSol    GDHIL-RQSTTCQK-DI-----PSGDFGFYLDDDELFYVDLD-TKEVVYRLPEIES--VF-SFE-----AQGGLQNL-AVCRHNLDIYIKRTNST-SGPI
W.A. lungfish MHC-IIabSol       GDHIL-RQSTTCQK-DI-----PSGDFGFYLDDDELFYVDLD-TKEVVYRLPEIES--VF-SFE-----AQGGLQNL-AVCRHNLDIYIKRTNST-SGPI
S.A. lungfish MHC-IIabSol       GDHIL-RQSTMCQK-DI-----PSGDFGFYFDGDEIFYVDLD-SKEAVYKLPDFEK--IM-SFE-----AQRGLQQL-AICNFNLDMYIKRSKGI-SGPK
Australian lungfish MHC-IIabSol GDHLL-RQNTLCQK-DT-----PTGEFGFYFDGDEIFYVDQD-SKEVVYRLPEFEK--AF-SFE-----AQAGLQSI-ALCQYNLDVFMKGTKGK-SGPK
Giant salamander DAA*0109       FDHVE-SQAIFVQT-QK-----PTGEYIFEYDKDELFHVDAD-RKEAEWRNPAFKD--FP-TVD-----IQGALGNF-AVLKTNLEISMKRSNNT-PATN
African clawed frog DAAf1       VDYFD-YGAMFYQS-YG-----PSGEYLFDYEGNEMFHVDLE-SKSVVWTLPGLEK--YT-SYD-----PQGGLQNI-NVAKYNLDGYKKSRNST-AATS
Gekko XP_015278405              AEEVL-SQVAFVQR-SLT-SEKGPGEFMFDFDGDEIFHVDLD-RKETVWRLPQFQE--FT-SFQ-----AEGAQGNI-AVLRSNLDILMRRSNNT-PAKN
Chicken B-LA                    KPHVL-LQAEFYQR-SEG-PDKAWAQFGFHFDADELFHVELD-AAQTVWRLPEFGR--FA-SFE-----AQGALQNM-AVGKQNLEVMISNSNRS-QQDF
Opossum XP_007483702            ENHVI-IQAEFAQT-SN-----PEGEFMFDFDGDEIFHVDMD-KRETVWRLTDFSN--FA-SFE-----AQGALANL-AVDKANLEIMMKRSNNT-PDTN
Mouse H2-Ag7 a1-domain          ADHVGFYGTTVYQS-PG-----DIGQYTHEFDGDELFYVDLD-KKKTVWRLPEFGQ--LI-LFE-----PQGGLQNI-AAEKHNLGILTKRSNFT-PATN
Human HLA-DR1 a1-domain         EEHVI-IQAEFYLN-PD-----QSGEFMFDFDGDEIFHVDMA-KKETVWRLEEFGR--FA-SFE-----AQGALANI-AVDKANLEIMTKRSNYT-PITN
Human HLA-DQ1 a1-domain         ADHVASCGVNLYQF-YG-----PSGQYTHEFDGDEQFYVDLE-RKETAWRWPEFSK--FG-GFD-----PQGALRNM-AVAKHNLNIMIKRYNST-AATN

II-b1/I-a2 lineage ancestral residues
                                                                              Y                              Q
                                                           r  Y               W      g                       E                    R
                                      g                    g  F               F      a                       D                  L K
MHC-II beta 1 domain
                                             Y   T                            Y                                   N
                                             F N S                            F                                   H
Nurse shark clone 11            GAHSE-ISLHRCVF-NST--GDWVFLKQQVYDQELIAYYDYN-QRKFIAVKAWMKS--NVDRWNR--EGAEEQYESGKAYCEHNIPIVY---ESA-LARQ
Ray EW694773                    GAHSL-TKIGGCAF-NSSAPGEWTYFSKGIYDGEVIWYFDFD-QRKFVAVKGWMKG--NMDRWNK--EAAESTYQRGLSMCENNIPLYG---REV-LPRR
Elephant shark  JW875734        EAESY-IFRSGCEFNNGT---SWTYVEEQIYNKQVIAYYDFN-QRKYIANKQWTKP--SVDTWN---QQAEETYQEGIRVCENNAQIFD---RIV-LTRL
Elephant shark JW875394         EADSN-IFRQGCEFNNGT---SWTFVYEEIYNKQLIAYYDFN-QRKYIAVKQWTKP--SVDSWN---QHAEETYQEGIRICENNVDVYN---RVE-LTRL
Bichir SRAs                     DGYMH-HVQRECRY-NSHSLEGMEFIDRYIFNHEEYIRYNST-LNKFIGYTEHGVK--NAEQANSDPSELAGQKSNVDATCKPNAKLYY---DTI-LDKS
Reedfish XP_028681436           EGYTY-HVQRECRF-NSHSLEGTEYIERRIFNKLEITRYNST-LGKFIGYTEIGVY--NADRFNNDSGVMAGMKSNVDAVCKRNAQIEY---DHI-LDKS
Paddlefish DAB*01               EGYLM-QTLVDCEY-SDSSMTDMVYSWNYVFNKQEVVHYDSK-IKKYVGNTACGVK--NAEVWNKDTAQLAGLLGDVDRYCKHNAELYM---LFT-TDRK
Trout DAB*1602                  DGYAS-DVVTRCLY-SSIDMHGAEFIQSYTFNKVEHLRFNST-VGEFVGYTELGLK--NAKRLNRG-QEVVQMRGELERLCEPNADVHY---RAI-LDKT
Zebrafish DAB1*01               DGYYQ-YTMLECIY-STSDYSDMVLLESGSFNKVVDVQYNST-VGKYVGYTEQGVI--FARNFNKNQAYLQQRKAEVESFCRHNAQISD---SAV-RDKA
Stickleback DAB*01              DGFMM-FVTDECVF-NSTELKDIEFIRSSYFNKKEDTRFSSS-VGKFVGFTEQGVK--IAANWNKDASFLSAMKAQKEVYCLNHVPVYY---TAA-LTKS
Coelacanth XP_006014459         ISNVE-QYQWECHYTNGT--QDIDFIHRVIYNGQEESYFDSR-IGKFTGVTEWGKK--DADYWNKDKENLAQWRIQEDRWCRNNYNWMQ---GWA-VGKQ
S.A. Lungfish GEHZ01038869      DGFVL-QLSKVCFFSGQQETKDMEFTYVFTFNKQIIMFYNQT-LGIFLEAGFYPIDD-LVDYMNRQTDLLKQLNTAVDTVCRSNMIKYW---DRT-AGRK
Slender lungfish MHC-IIabSol    GVFLH-QITADCLYMREG--QKLVFLQTTLYNKERQVYFDSR-IGKFIGITEFGKI--EAEYWNNDTAYLEKMRAAIMIFCKHNFDLLN---NTI-MDRK
W.A. lungfish MHC-IIabSol       GVFLH-QITADCLYMREG--QKLVFLQTTLYNKERQVYFDSR-IGKFIGITEFGKI--EAEYWNNDTAYLEKMRAAIMMFCKHNFDLLN---NTI-MDRK
S.A. lungfish MHC-IIabSol       RVHLY-QTIVDCLYVNGG--QTVILLQTIVYNRKKQLYFDSR-VGKFIGVTELGKT--DAEYFNNNTAYLEQLMEEVVTVCKYNYDIMK---VVI-VDRK
Australian lungfish MHC-IIabSol GVYLL-QQISDCLYKNDG--QDVVLLYRVIYNKEEQLYFDSR-VGKFIGITEFGKF--QAEYWNNDTAYLAQLRASVDTYCRHNYELMK---TSV-LDRK
Giant salamander AGY56015       ADFVT-QAKAECHFLNGS--ERVRFLLRYFYNQEELAYFDSD-VGRFVPKTEFGKP--DADYWNSDPAILEDARAAVERYCKHNYDVRK---QDA-RSWK
African clawed frog DAB         EDYVY-QYKAQCYFRNGT--DNVRLLWRHYYNLEETDYFDSD-VGLFIAKTELGKP--SADYWNSQKETLEQKRAAVDTVCRHNYPFDK---PFT-IDRK
Chicken B-LBII                  AFFFC-GAISECHYLNGT--ERVRYLQRYIYNRQQYAHFDSD-VGKFVADSPLGEP--QAEYWNSNAELLENRMNEVDRFCRHNYGGVE---SFT-VQRS
Mouse H2-Ag7 b1 domain          RHFVH-QFKGECYFTNGT--QRIRLVTRYIYNREEYLRFDSD-VGEYRAVTELGRH--SAEYYNKQ--YLERTRAELDTACRHNYEETE--VPTS-LRRL
Human HLA-DR1 b1 domain         PRFLW-QLKFECHFFNGT--ERVRLLERCIYNQEESVRFDSD-VGEYRAVTELGRP--DAEYWNSQKDLLEQRRAAVDTYCRHNYGVGE---SFT-VQRR
Human HLA-DQ1 b1-domain         EDFVY-QFKGLCYFTNGT--ERVRGVTRHIYNREEYVRFDSD-VGVYRAVTPQGRP--VAEYWNSQKEVLEGARASVDRVCRHNYEVAY---RGI-LQRR
res. number HLA-A2 a1           .1        .10         .20        .30      .40      .50        .60          .70         .80      .90
res. number HLA-A2 (a2)*                  .100        .110      .120       .130      .140         .150      .160      .170       .180
```

Supplementary file 4B(b)

Alignment of deduced MHC-II and lungfish MHC-IIabSol IgSF amino acid sequences

```
Probable homodimer residues
(red for an MHC characteristic residue)
                                                                                        N                  N      Q  S
                                                         V   Y         RN          T    E N TY             E Y    E  G
                                  P  V v        g    L C A GFFP  I   W KDG         S    D DgSF     L   P  GD F C VDH AL   P    W
                                        .10       .20       .30       .40       .50       .60       .70       .80       .90
II-a2/b2-m residues
                                                        Y
                                        Y             L H
                                       vF       g  N  I F      P                                     F
MHC-II alpha 2 domain
                                                                             T       Y
                                             V                               S G     F          F
Nurse shark pSa5-1              VPPE-VSVYSEDLVEWGQLNTLICFADGFYPPHITMKWRRNNEPMTDGDNITEFYIKDDFTYRRFSYLSIVPSPGDMYSCHVEHSSLQ-DPVTVFW
Little skate FL670178           VPPQ-TAMYPEDPVEWGESNTLICLADGFYPPRINMKWKRNNEPVTEGIDTTEYYVKRDYTFQRFTYLNFIPSPGDMYSCHVEHEGLE-RPTTVFW
Elephant shark AFM88468         IQPD-VAVFPEDNVEWGVMNTLICHAAGFFPAALEMQWLRNNQKVTEGVNITEYYMEEDYSFQRFTYLSFVPRPGDEYTSRVQHRSLD-QPAVYFW
Elephant shark AFM88844         IQPD-VAVFPEDNVEWGVMNTLICHAAGFQPAALEMQWLRNNQKVTEGVNITEYYMEEDFSFQRFTYLSFVPRPGDEYTCRVQHRSLD-QPAVYFW
Bichir SRAs                     ARPR-VAVYPAYDLELGISNTLVCFVTGFYPIPIKLSWYKNNQLISDGVELSRYYPNEDLTFQIFSEINFMPKVGDIYSCMVKHSSDS-DLITAIW
Reedfish XP_028681425           AIPR-VVMYPAYDLEMGISNTLVCFVTGFYPVPIKLSWYKNSQPISDGVEVSRYYPNDDFTFQIFSEISFMPKVGDIYSCMVEHSSVT-DLITTIW
White sturgeon DR975335         VSPR-VTLYPEKDLELGKPNTLICFITDFHPAAIKVTWTKNTLPVTEGVSLTQYYSNKDFTLQMFSYLSFTPELGDVYSCQVQHSALP-ETLTTFW
Zebrafish a4                    DPPQ-TSIYSRDDVQPDIENKLICHVTGFFPPPVRVSWTKNNEIVTEGMSVSQYRPNNDGTYNIFSTLRFTPVEGDIYSCSVNHKTLE-QPQTKAW
Trout DAA*0101                  DPPH-SSIYPRDDVELGVENTLICHVSGFFPSPVRVRWTRNDQNVTEGGRISTPYPNTDVTFNQFSSLSFTPEEGDIYGCTVEHKALT-EPLTRIW
Stickleback DAA*01              DPPSSHMIYPKDGVELGEKNSLICHVTGFYPAPVTFSWTKNQENVNEGSSRNVPFPNNDGTFNQFSTLEFTPKLGDIYSCMVEHLALD-HPLVKFY
Coelacanth XP_006014228         VPPE-VTMYPENQVEFGKANILICFMDNFYPPVLNFTWYKNGNQVSEGFYNTDFYSKVNYRFRKFSYLNFTPEVGDIYSCQVEHWGLE-EPINKFW
S.A. Lungfish GEHZ01055957      VKPEFIVVYPEEPLEPETPNKLICSVDRLYPPVLNITWYRNGNPVYEGVEMTSFYPRDDAYFRIFSFLNFIPTDNDIYSCKVEHPGLQ-SPAEKFW
Slender lungfish MHC-IIabSol    VPPV-VKVYPMQHVKPGKPNTLICATEDFFPCVLNISWSKNGIPVKEGITVTDFYPGKEFTFKRFAYLTFIPVQGDIYSCNVEHPGLD-RPTTVFW
W.A. lungfish MHC-IIabSol       VPPV-VKVYPMQHVKPGKPNTLICATEDFFPCVLNISWSKNGIPVKEGITVTDFYPGKEFTFKRFAYLTFIPVQGDIYSCTVEHPGLD-RPTTVFW
S.A. lungfish MHC-IIabSol       VLPV-VKVYPMQHVDPGKPNTLICSTEDFFPCVLNISWSKNGIPVKEGITVTDFYPGKEFTFKRFAYLTFIPVPGDIYSCTVEHPGLD-RPTTVFW
Australian lungfish MHC-IIabSol VPPM-VKVYPAQSVDLGKPNTLICSMDGFFPPVLNVSWSKNDNPVEEGVQITDFYPGEDSAFERFAYLTFIPDQGDIYSCTVEHPALD-TPDTVFW
Giant salamander DAA*0109       VPPA-VTVFPKEPVELGQPNILVCFMKDFFPPAINVTWLKNTQPVTAGVGEADFFPVQDGSFCKFHYLTFVADPDDVYACSVEHWGLT-EPMITMW
African clawed frog DAAf1       IPPL-ITLYSAKPVVQGEPNILICCVNNIFPPVMNTTWFKNGQKISDGFSETSFLPAKDYSFGRLHYLAFLPNEHDIYTCEVEHWGLD-RPTRRVW
Gekko XP_015278405              VPPK-VLVYPEKPVVLGEPNVLICLANEFSPPVISLTWVKNGQAV-KATEETDFYPNTDNSFRKFSYLIFVPNAEDIYYCRVEHWGLA-QPLTKEW
Chicken B-LA                    VTPE-LALFPAEAVSLEEPNVLICYADKFWPPVATMEWRRNGAVVSEGVYDSVYYGRPDLLFRKFSYLPFVPQRGDVYSCAVRHWGAE-GPVQRMW
Opossum  XP_007483702           VPPE-VTVFPKGPVELGHPNVLVCFIDKFSPPVLNVTWLQNGKPITDGVFETVFLPRADHSFRKFHYLTFIPSATDFYDCEVDHWGLE-QPTIKHW
Mouse H2-Ag7 a2-domain          EAPQ-ATVFPKSPVLLGQPNTLICFVDNIFPPVINITWLRNSKSVTDGVYETSFLVNRDHSFHKLSYLTFIPSDDDIYDCKVEHWGLE-EPVLKHW
Human HLA-DR1 a2-domain         VPPE-VTVLTNSPVELREPNVLICFIDKFTPPVVNVTWLRNGKPVTTGVSETVFLPREDHLFRKFHYLPFLPSTEDVYDCRVEHWGLD-EPLLKHW
Human HLA-DQ1 a2-domain         EVPE-VTVFSKSPVTLGQPNTLICLVDNIFPPVVNITWLSNGQSVTEGVSETSFLSKSDHSFFKISYLTFLPSADEIYDCKVEHWGLD-QPLLKHW

II-b2/I-a3 residues
                                                                                                    D
                                                                                  T                 E
                                                                                  S                 Q
MHC-II beta 2 domain
                                                                                         G W                         s
Nurse shark clone 11            VEPK-VTIRTKESTYPGPSAILICYAVGFYPAKISVTWLKNGQKVSDADVTVELLSNGDWTYQVRQYLQYEPVYGDKYTCHVEHSSLT-SPMSVDW
Ray EW694773                    VEPE-ITVRPKVSSHSGPSALLTCHVTGFYPPEIEVKWLKNGAPVPTGAINTVLLSDGDWTYQVEELLQYHPVSGDVYTCHVEHVSLT-EPMTVDW
Elephant shark JW875734         EKPT-VTIRPKQSAHTG--HLLTCYVTDFYPKEIEVTWRRNGHVVNGDQVSTSLMANGDWTYQIHKVLQYSPESGDHYSCHVHHKSLD-SDIDHPW
Elephant shark JW875394         VKPR-VTIRPKQSAHTG--HLLTCYVRGFYPKEIEVTWRRNGHVVNGDQVSTSLMANGDWTYQIHKVLQYSPESGDHYSCHVHHKSLD-SDIDHPW
Bichir SRAs                     VKPT-VTVKAVKPEGSSHEYMLVCSALGFYPRKIKISWLKNGVEVKSGVTSTEEVSNGNWYYQIHSHWEMTPQSGETYACKVQHRSVSDQPIIVQW
Reedfish XP_028681436           VKPT-VTVKATKPDGSSHEYMLVCSALGFFPRKIKVTWLRNGVEVKSGVTSTEEMSNGNWYYQIHSHWEMTPQSGETYACKVQHKSVSDQPIIVQW
Paddlefish DAB*01               IPPS-VRVRSMKPFSSQHQTMLVCNAFGFYPREIQMTWLRNGVKVTADVSSSELLSDGDWTYQIHSYLELTPQSGDSYSCRVEHSSLA-EAIEVKW
Trout DAB*1602                  VEPH-VRLSSVTPPSGRHPAMLMCSAYDFYPKPIRVTWLRDGREVKSDVTSTEELANGDWYYQIHSHLEYTPKSGEKISCMVEHISLT-EPMMYHW
Zebrafish DAB1*01               VKPK-VTIQSVMQAEGKHPAMLLCDAYEFYPKKIKMSWLRDDKVVTSDVTSTIEMANGNWYYQIHSHLEYTPKSGEKIQCVVEHASST-QPITKEW
Stickleback DAB*01              AEPY-VRLHSETPPGGGPLSMLVCSVYDFYPKKIIVRWTRDGRPETTGVTSTDELADGDWYYQTHSHLEYTPRSGEKISCVVEHISLS-KPLVTDW
Coelacanth XP_006014459         ISPA-AVITPTKGMSSSHPNMLVCYVTGFFPSKITVTWFRNGKEVDSHVTSSELLQNGDWTYQIHVFLEMTPKSGDVYTCRVEHSSLE-DPMELTW
S.A. Lungfish GEHZ01038869      AKPS-VTIESLVPQVSTYSTRLVCHVHGFYPEEIIVKWYKNGEEY-PQSTTTAVWPNGDWTYQVKSYMEFTPHMRDEYMCVVKHNSLD-GSLIVKW
Slender lungfish MHC-IIabSol    VIPV-VTMQPLRSVIEKHPNLLMCEASGFYPQSIRVTMLKNGEEVTRDISSSGLISNGDWTYQITLLMEFIPKKEDVYGCKVEHSSLN-EPMIVEW
W.A. lungfish MHC-IIabSol       VIPV-VTMQPLRSVIEKHPNLLMCEASGFYPQSIRVTMLKNGEEVTRDISSSGLISNGDWTYQITLLMEFIPKKEDVYGCKVEHSSLN-EPMIVEW
S.A. lungfish MHC-IIabSol       VIPV-ITVRSLRKVLEKHPNLLMCAASGFYPQSINVTMFMNGQEVTHDISSSGVISNGDWTYQITLLMEFLPKKEDVYSCKVEHSSLK-EPVIVEW
Australian lungfish MHC-IIabSol VSPT-VTVQPFRIVKEKRPNLLICATNGFYPQNINVTLLKNGEEVTHDISSSGVISNGDWTYQIILLMEFIPKRADVYSCKVEHSSLK-EPLIVDW
Giant salamander AGY56015       VIPK-VRITPTKTMSLDHQTMLVCYVEDFYPPAINISWIKNGKEETGSVMSTEMLQNGDWTYQIHLFLETTPERGDTYVCQVEHSSLK-SPITVEW
African clawed frog DAB         SQPN-VKIVNTKTLDLEHENLITCFVGGFFPPLIKVTWLKNGIEEGEQVTSSELLQNGDWTFEIHVMLETTIKHGDTFTCRVEHSSLQ-QPVYLNW
Chicken B-LBII                  VEPK-VRVSALQSGSLPETDRLACYVTGFYPPEIEVKWFLNGREETERVVSTDVMQNGDWTYQVLVVLETVPRRGDSYVCRVEHASLR-QPISQAW
Mouse H2-Ag7 b2 domain          EQPN-VAISLSRTEALNHHNTLVCSVTDFYPAKIKVRWFRNGQEETVGVSSTQLIRNGDWTFQVLVMLEMTPHQGEVYTCHVEHPSLK-SPITVEW
Human HLA-DR1 b2 domain         VEPK-VTVYPSKTQPLQHHNLLVCSVSGFYPGSIEVRWFRNGQEEKAGVVSTGLIQNGDWTFQTLVMLETVPRSGEVYTCQVEHPSVT-SPLTVEW
Human HLA-DQ1 b2-domain         VEPT-VTISPSRTEALNHHNLLICSVTDFYPSQIKVRWFRNDQEETAGVVSTPLIRNGDWTFQILVMLEMTPQRGDVYTCHVEHPSLQ-SPITVEW
res. number HLA-A2 b2m                  .10       .20       .30       .40       .50       .60       .70       .80       .90
res. number HLA-A2 a3*                  .190      .200      .210      .220      .230      .240      .250      .260      .270
```

Supplementary file 4C(a)

NJ phylogenetic tree of α1 and β1 domains of lungfish MHC-IIabSol and classical MHC-II

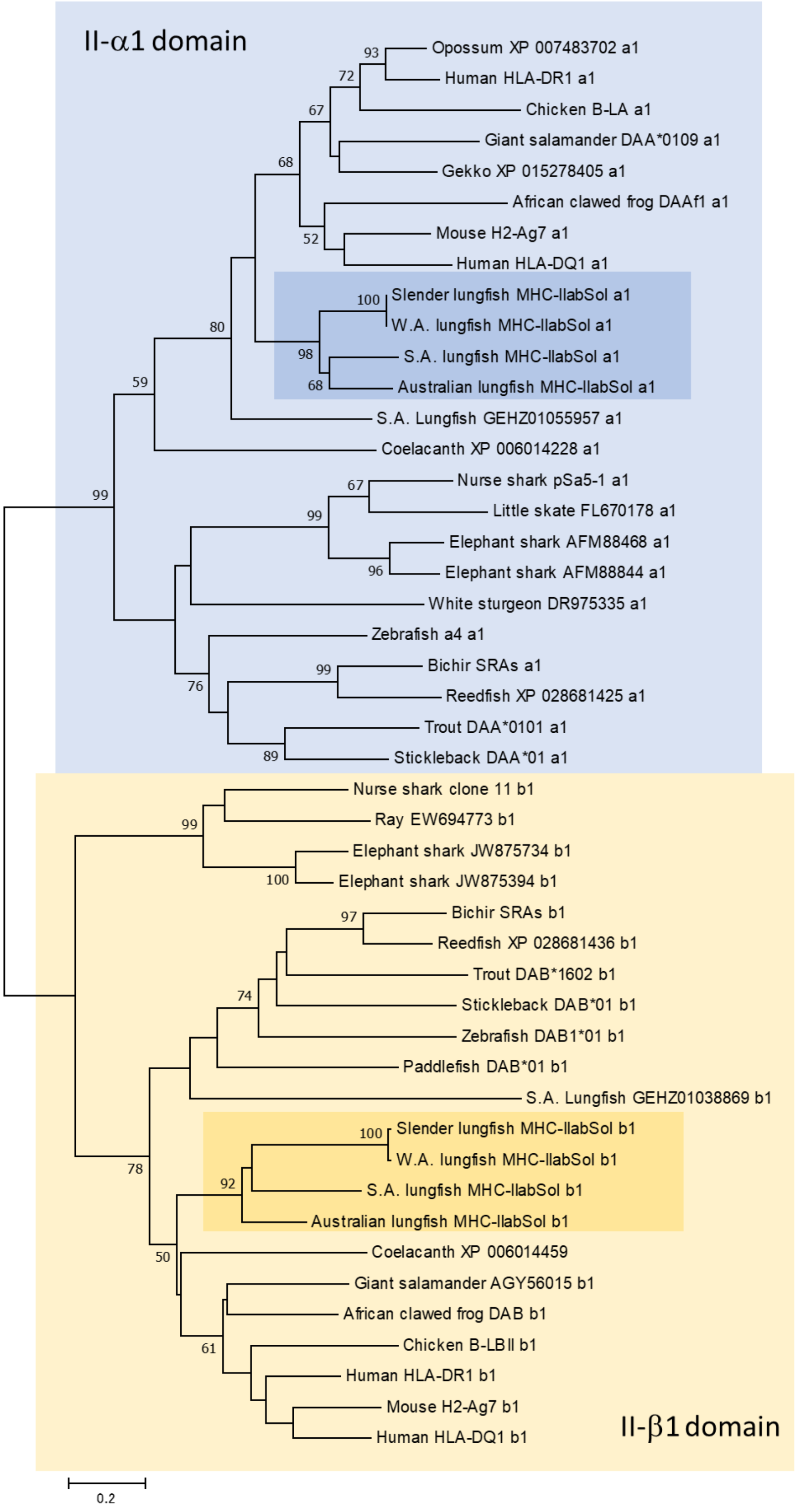

Supplementary file 4C(b)

NJ phylogenetic tree of α2 and β2 domains of lungfish MHC-IIabSol and classical MHC-II

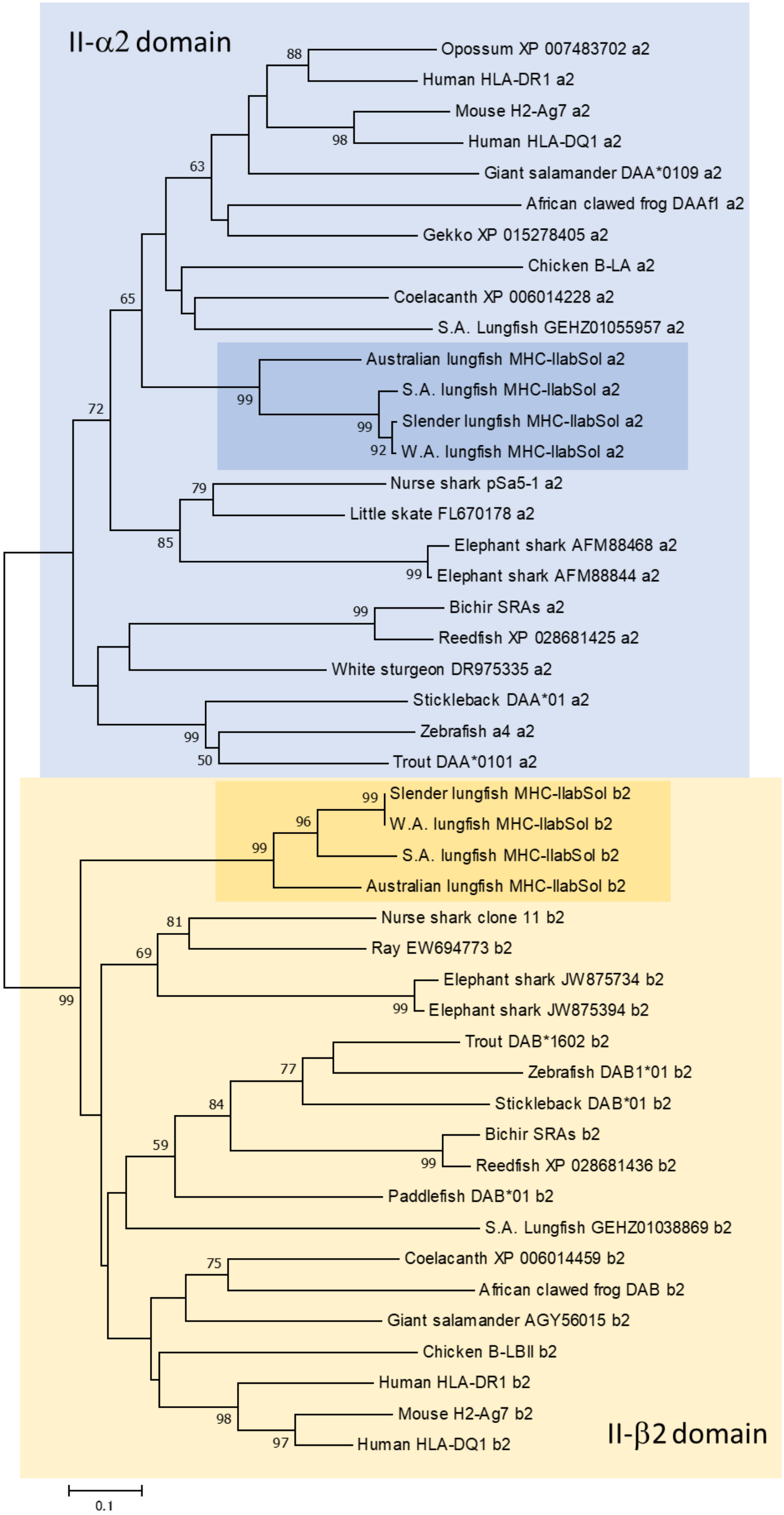

Supplementary file 4C(c)

NJ phylogenetic tree of (artificial) MHC-II $\alpha 1+\alpha 2+\beta 1+\beta 2$ combinations of MHC-IIα and MHC-IIβ sequences from identical or similar species

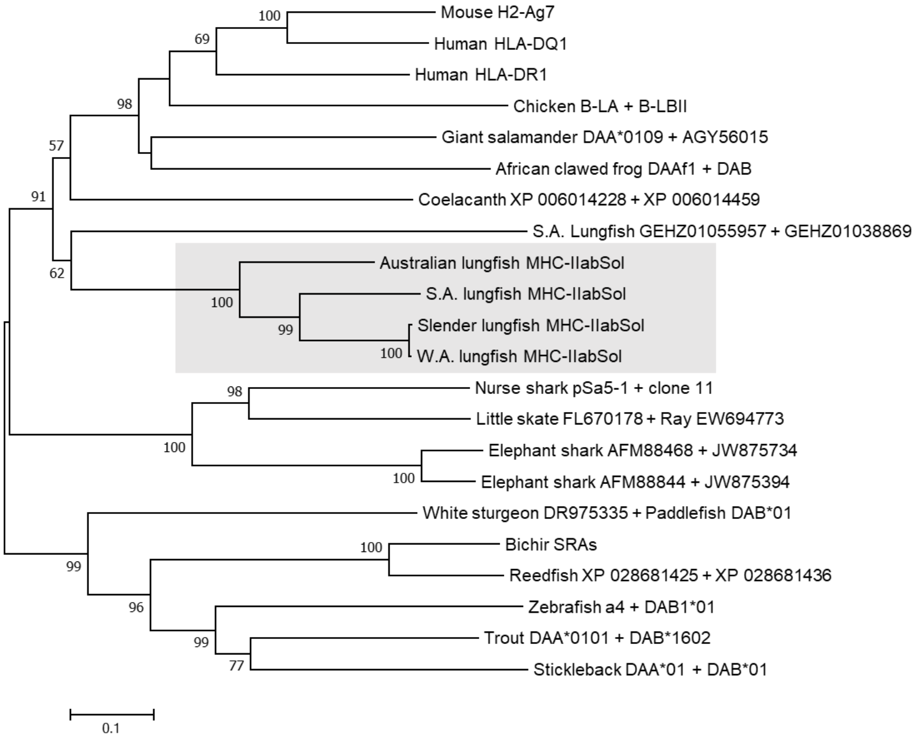

Supplement: Supplementary file 1 [file DataSheet_1.zip › Supplementary File 4.pdf]
