# Supplementary material for: Structural Comparison Between MHC Classes I and II; in Evolution, a Class-II-Like Molecule Probably Came First
Source: Front Immunol. 2021 Jun 14;12:621153. doi: 10.3389/fimmu.2021.621153 (PMC8236899; doi:10.3389/fimmu.2021.621153)

Supplementary file 5

Model for pMHC evolution from the ancestral MHC homodimer to extant MHC-II and MHC-I

Table of Contents **Page**

Figure legend 2

Model stage 1: The structure of the ancestral MHC homodimer ectodomain complex 3

Model stage 2: The immediate (last common) ancestor of both MHC-II and MHC-I 4

Model stage 3A: Extant MHC-II 4

Model stage 3B: Extant MHC-I 5

Figure legend

This file presents a model for the structural evolution of the ectodomain part of a presumed ancestral MHC homodimer to extant MHC-II and MHC-I structures. The model is based on the "II-to-I" evolution model that was postulated based on exon organizations and overall sequence similarities (Kaufman et al. 1984; 1988; Hughes and Nei 1993) (see the main text and Supplementary file 1). To our knowledge, at the detailed structural level, other than the covalent organization of domains, such model has not been discussed before.

The model is divided into: Stage 1, the presumed ancestral homodimer; Stage 2, the immediate ancestor of both MHC-I and MHC-II; Stage 3A, extant MHC-II; and Stage 3B, extant MHC-I.

The figures are in gray shading to stress that these do not represent actual structures but estimations or generalizations, and the details of the structures other than the highlighted features should not be overinterpreted. In cases where a single amino acid represents a larger set of amino acids, this is indicated by underlining the name of the amino acid that is actually shown.

References used in this file

Kaufman JF, Auffray C, Korman AJ, Shackelford DA, Strominger J (1984) The class II molecules of the human and murine major histocompatibility complex. Cell 36(1):1-13.

Kaufman, J (1988) Vertebrates and the evolution of the Major Histocompatibility Complex (MHC) class I and class II molecules. Verh Dtsch Zool Ges 81:131-144.

Hughes AL, Nei M (1993) Evolutionary relationships of the classes of major histocompatibility complex genes. Immunogenetics 37(5):337-346.

Model

Stage 1: The structure of the ancestral MHC homodimer ectodomain complex

(The ancestral MHC homodimer complex probably included two identical transmembrane molecules with p-i-CP/TM/CY organization and a p+p peptide-binding domain (PBD) with a groove for binding peptides that could extend beyond the groove ends).

(a) overall structure

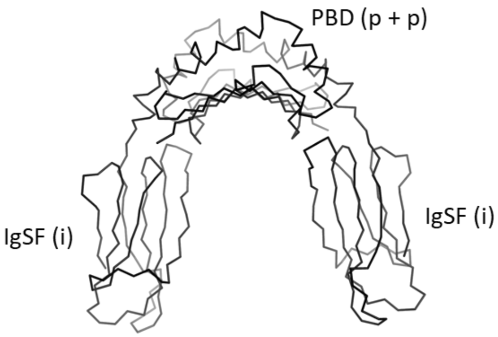

Probably a symmetric structure. As in extant MHC molecules, the PDB domain consisted of two — but in this case identical — p domains and formed an 8-strand curved β-sheet topped by two α-helical structures that left a groove between them. The orientations of the IgSF domains are not known.

(b) peptide orientation

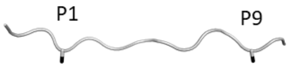

The peptide extended beyond the groove and the P1 and P9 position residues had their sidechains orientated downwards into the groove, similar as in extant pMHC-II. *Peptide main chain in cartoon format, P1 and P9 sidechains until Cβ (black) in sticks format.*

(c) p-domain helices

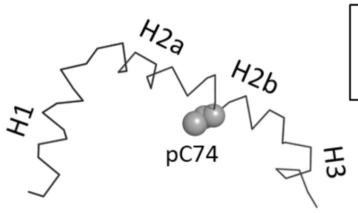

The organization of helices of each p domain probably was similar as in pb domains of extant pMHC-I and pMHC-II. Helices H1, H2a, H2b and H3 were separated by kinks and folded over the curved β-sheet.

(d) peptide binding residues

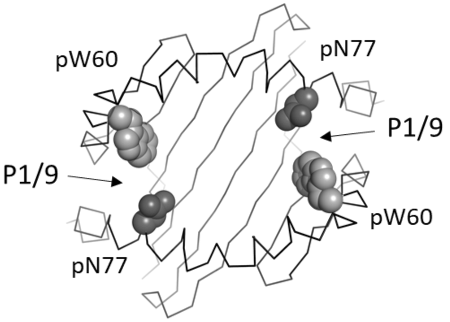

Groove with pW60 and pN77 residues that could form hydrogen bonds with the peptide ligand main chain in either orientation. The groove bound peptide fragments of 9 aa and pockets P1/9 bound the side-chains of peptide ligand residues P1 and P9. The groove ends were open and most of the bound peptides were longer than 9 aa and extended beyond the P1/9 pockets as in extant pMHC-II. We speculate that the H1 helices ran above and parallel to the β-strand helices S4 as is the case in pb domains of extant pMHC-I and pMHC-II.

(e) p-domain β-sheet

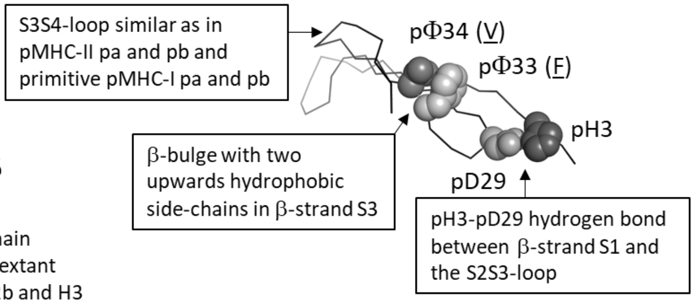

This figure shows the β-sheet part contributed by a single p domain, and the highlighted features can be concluded for the homodimer ancestor with relative certainty. At positions p33 and p34 probably hydrophobic residues were found, and the p33F and p34V residues shown are just possible examples

(f) IgSF domain

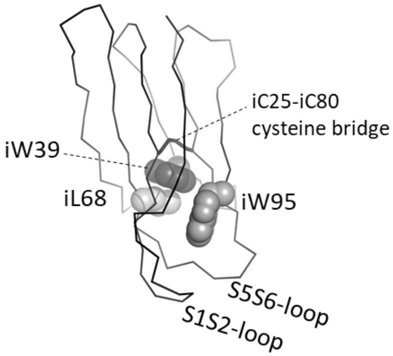

The homodimer IgSF domain presumably had a structure similar as in extant pMHC-II α2 domain, with an IgSF typical core including the iC25-iC80 cysteine bridge and the hydrophobic interacting residues iW39 and iL68. The orientation of the S1S2 plus S5S6 loops was unusual compared to other C1 category IgSF domains and included a unique iW95 residue.

Stage 2. The immediate (last common) ancestor of both MHC-I and MHC-II

(A complex including two different but similar transmembrane molecules with pa-ia-CP/TM/CY and pb-ib-CP/TM/CY organization and a pa+pb peptide-binding domain (PBD) with a groove for binding peptides that could extend beyond the groove end. Most peptides were bound with their N-to-C direction in P1-to-P9 pockets direction because such is found in both extant pMHC-II and extant pMHC-I.)

After duplication and differentiation of the original MHC gene into an α-chain and a β-chain gene, the MHC complex now comprised a heterodimer.
The major structural changes compared to the homodimer ancestor probably were:

(a) A large reorientation of the ia domain and the establishment of new interdomain contact regions.

(b) We speculate that the kink between pa domain helices H2a and H2b was lost, and that pa domain helix H1 started to run more perpendicular to the β-sheet instead of parallel to β-strand S4 as assumed for the homodimer ancestor. However, because of differences between the organization in the pa helices in extant pMHC-II and pMHC-I, it is difficult to be certain about this. Therefore, we did not attempt to show the pa helical organization expected at this evolutionary stage in a figure.

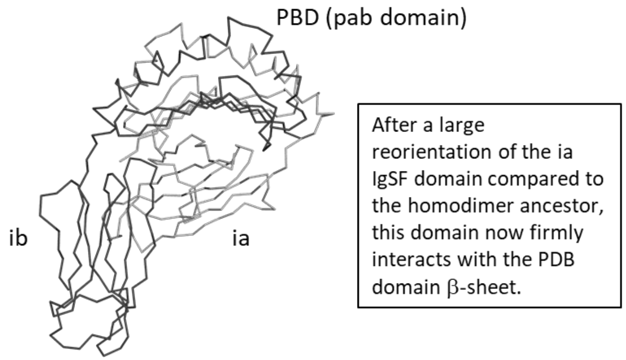

After a large reorientation of the ia IgSF domain compared to the homodimer ancestor, this domain now firmly interacts with the PDB domain β-sheet.

Stage 3A. Extant MHC-II

(A complex including two different but similar transmembrane molecules with pa-ia-CP/TM/CY and pb-ib-CP/TM/CY organization and a pa+pb peptide-binding domain (PBD) with a groove for binding peptides that could extend beyond the groove end. Most peptides are bound with their N-to-C direction in P1-to-P9 pockets direction.)

Compared to the MHC-I/II immediate ancestor, the major changes for establishing the extant pMHC-II structure were:

(a) Sequence deletion accompanied by partial melting of pa domain helices H1 and H2, and loss of ancestral paW60. In many but not all MHC-II molecules the pa domain cysteine pair was lost.

(b) Acquisition of residues pa(D/N)70 and probably also pb(H/N)76 for forming hydrogen bonds with the peptide ligand main chain, so that the core set of residues for binding the peptide ligand main chain became pa(D/N7)0, paN77, pbW60, pb(H/N)76, and pbN77.

(c) The connection between the ib domain and the pab domain was strengthened under the P1 side of the pab domain by the change of the ancestral ia60 residue into iaW60 which inserts into the pab domain and interacts with the newly acquired/fixated residues pa(D/E/N)31, paE32, pa(I/L)49 and paF52.

figure for (a) and (b)
top-view of binding groove

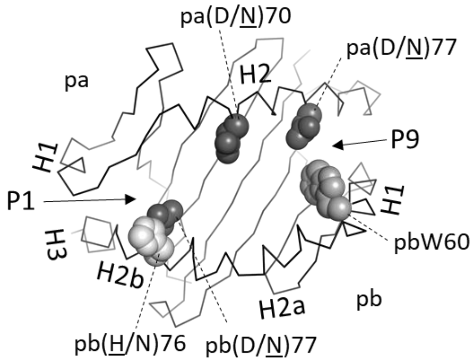

figure for (c)
ibW60 inserts into the pab domain

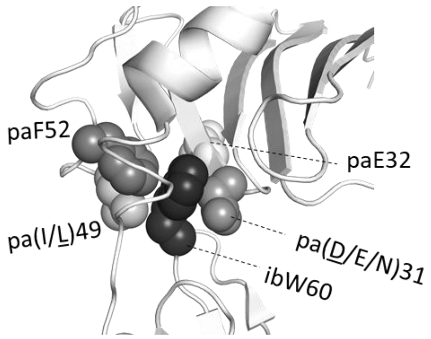

Stage 3B. Extant MHC-I

(A complex of a heavy chain transmembrane molecule with pa-pb-ib-CP/TM/CY organization, and a single domain soluble ia domain [β_2-m]. The pa+pb peptide-binding domain [PBD] forms a groove for binding peptides of ~9 aa that cannot extend beyond the groove end. Peptides are bound with their N-to-C direction in P1-to-P9 pockets direction.)

Besides the necessary exon shuffling event (see Supplementary file 1), compared to the MHC-I/II immediate ancestor, the major changes for establishing the extant pMHC-I structure were (see also the next page):

(a) Pa helices: It is not clear when all fundamentals of the MHC-I type pa helix organization were established (see the discussion at Stage 2 of this model), but relative to the deduced homodimer ancestor in pMHC-I the kink between pa helices H1 and H2 is at a more N-terminal position and the kink between helices H2a and H2b was lost. Through the reorientation of helices the ancestral pW60 residue no longer forms a direct part of the groove and the pa59 side-chain points to the bottom of the groove. At the C-terminal end of the pa helical region the strand now loops backwards to continue as β-strand S1 of the pb domain, and the bulkiness of the loop structure, including the newly acquired paQ87, seems to give a slight upwards lift of the end of the pa helix H2 compared to pMHC-II.
The paC11-paC74 cysteine bridge was lost.

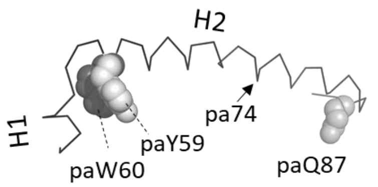

(b) Pb helices: Compared to the pb helix organization in the hypothesized homodimer ancestor and MHC-I/II immediate ancestor, the MHC-I pb helices did not change much. However, involving the acquisition of the pbG10 residue, the pbC11-pbC74 cysteine bridge was pushed slightly upwards causing a slight turn in the H2b helix so that the newly acquired pbW77 is situated a bit higher and its side-chain can block the groove. The H2b and H3 helices are at a sharp angle with a newly acquired pbG85 at their border.

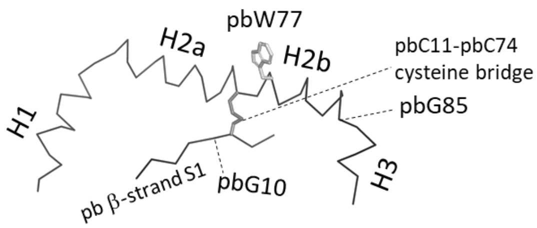

(c) Groove: At the P9 side, the binding remained relatively similar as in the homodimer ancestor, with pa(D/N/S)77 and pbW60 still able to bind the peptide ligand main chain by hydrogen bonds, although in addition such bonds now also can be formed by the newly acquired/fixated pa(Y/R)84, pbT56, and pbK59. Because the pa H2 helical region ends a bit higher than in pMHC-II, the pa(Y/R)84 residues are now involved in blocking the extensions of peptide ligands beyond the P9 pocket, something which similar amino acids if at position pa84 in pMHC-II don't do.
At the P1 side, in contrast to the P9 side, very big changes occurred compared to the ancestral stages. A slight rotation of pb helix H2b (see above) combined with the exchange of ancestral pbN77 for pbW77 blocks the groove, and the newly acquired/fixated paY59 residue points downward for participating in a hydrogen bond network with the peptide ligand P1 main chain at the bottom of the groove. Other newly acquired/fixated residues participating in that hydrogen bond network are paY7, pa(E/N/Q)63, pbY70, and pbY81 (the peptide ligand and network are not shown here). The reason that paY7 — which resides in pa β-strand S1 — can reach towards the P1 pocket is because of absence of a side-chain in the newly acquired paG26 over which it bends. Among pMHC-I structures the P1 pocket is very similar in the precise orientation of the key residues, whereas in the P9 pocket more plasticity is observed.
The gray arrow in the figure points at the position where in the pb b-sheet the S3 parts from the S2 strand at an earlier position than in pMHC-I pa or in pMHC-II pb (the "S2-S3-cleft"), something which may give extra flexibility to the part of the groove supported by pb β-strands S3 and S4.

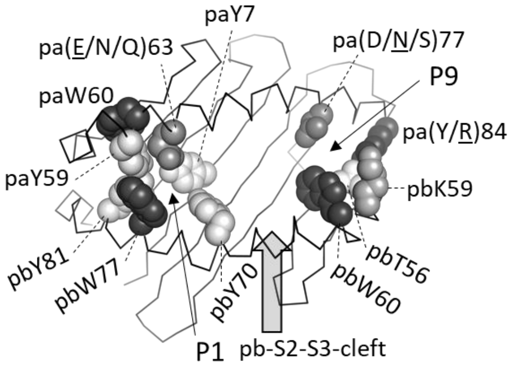

Stage 3B. Extant MHC-I (continued)

(d) Peptide orientation: The bound peptide ligand is ~9 aa and usually does not extend beyond the P1 and P9 pockets. Compared to the ancestral MHC situation, the P1 pocket has slightly shifted towards the center of the pab domain (indicated by the arrow), and the P1 residue main chain is now bound at the bottom of the groove instead of at the top of the groove (see stage 1). This results in the P1 side-chain pointing upwards, and in difficulty for the groove to bind the peptide in a relaxed configuration which commonly results in bulging of the middle part of the peptide. In the figure, the peptide main chain is shown in cartoon format, and the P1 and P9 side-chains until Cβ (in black color) in sticks format.

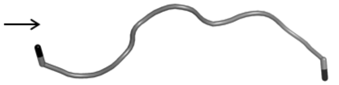

(e) The interdomain contacts: There are three unique pMHC-I interdomain contacts, the positions of which can be understood from the whole structure figure (e1): (1) The hydrogen bond between paR48 and iaD53, although this bond is not perfectly conserved; (2) An impressively large and conserved interaction with as central point the insertion of the iaF56+iaW60 hydrophobic knob into the "pa9" pleat of the pab β-sheet and involving hydrogen bonds between iaW60 and the pb residues pbQ6 and pbD32; (3) A contact between the ia and ib domains with as central and well-conserved point the hydrogen-bond between iaY10 and ibP56.

In Fig. e2 the above interactions numbers 1 and 2 are shown in more detail, showing β_2-m and the β-sheet of the pab domain from above. The residues at the ridges of the pa9 pleat are in black. Residues involved in the interactions which show conserved features are highlighted by showing their side-chains and Cα atoms in spheres format for the ia domain, and by showing their side-chains in sticks format for the pab domain. Polar contacts between paR48 and iaD53, between pbQ6 and iaW60, and between pbD32 and iaW60, are shown with dashed black lines. With exception of paR48 and ia(F/Y)62, all the contact region residues that are highlighted in the figure by showing their side-chains are unique to - or better conserved in - MHC-I as compared to MHC-II. With exception of paR48, pbQ6, pb(Q/R)25, and ia(F/Y)62, all the highlighted residues may have been acquired early in MHC-I evolution. Compared to the MHC-I/II immediate ancestor, the biggest change in the interdomain contacts was probably the insertion of the iaF56+iaW60 knob into the pab pa9 pleat, since in pMHC-II neither such ia residues nor such insertion is observed.

In Fig. e3, the ia-ib interaction is shown with the highly conserved and pMHC-I-specific hydrogen bond between iaY10 and ibP56 (dashed black line). The ibP56 residue also makes ring-ring contact with the ancestral ia(F/H/Y)26 residue. The acquisition of this proline and the precise position of ib56 are unique to pMHC-I, but some other residues at this interdomain contact area such as the ones highlighted here besides ibP56 can also be found in pMHC-II.

(e1)

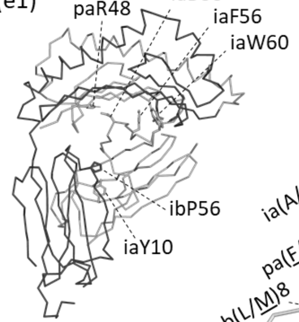

(e2)

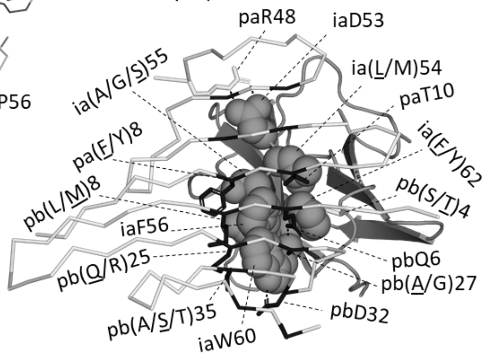

(e3)

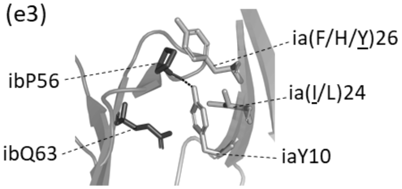

(f) The IgSF ia domain internal structures:

The overall shape of the MHC-I ia domain (β_2-m) is well-conserved and similar as hypothesized for the i-domain of the homodimer ancestor. However, the rigidity may have decreased, since (1) of the internal hydrophobic core the IgSF typical iW39 and iL68 were lost and replaced by smaller residues and (2) in many ia sequences the MHC i-domain typical iW95 residue was replaced by a smaller leucine (e.g. in shark β_2-m) (Fig. f1).

Compared to the hypothesized homodimer ancestor, most of the MHC-I ib domain (I-α3) structure is well conserved, except for the S1S2-loop in which a lot of variation is found. However, as in the MHC-I ia domains, also in the MHC-I ib domains the rigidity may have decreased, since (1) in many MHC-I molecules ibL68 was replaced by a smaller residue and in some (including in shark UAA) also ibW39 was replaced, and (2) in many ib sequences the MHC i-domain typical iW95 residue was replaced (including in shark UAA) or it lost its original orientation (see example in Fig. f2).

(f1)

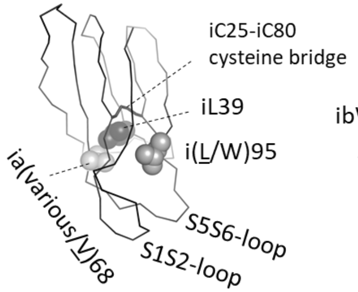

(f2)

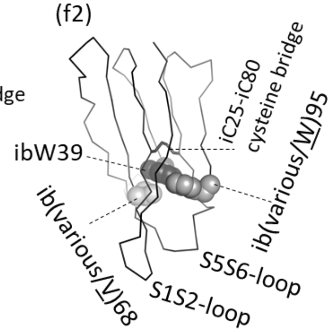

Supplement: Supplementary file 1 [file DataSheet_1.zip › Supplementary File 5.pdf]
